# Evolution-Based Functional Decomposition of Proteins

**DOI:** 10.1371/journal.pcbi.1004817

**Published:** 2016-06-02

**Authors:** Olivier Rivoire, Kimberly A. Reynolds, Rama Ranganathan

**Affiliations:** 1 Laboratoire Interdisciplinaire de Physique, CNRS and Université Grenoble Alpes, Grenoble, France; 2 The Green Center for Systems Biology, and Department of Biophysics, University of Texas Southwestern Medical Center, Dallas, Texas, United States of America; 3 The Green Center for Systems Biology, and Departments of Biophysics and Pharmacology, University of Texas Southwestern Medical Center, Dallas, Texas, United States of America; University of North Texas System College of Pharmacy, UNITED STATES

## Abstract

The essential biological properties of proteins—folding, biochemical activities, and the capacity to adapt—arise from the global pattern of interactions between amino acid residues. The statistical coupling analysis (SCA) is an approach to defining this pattern that involves the study of amino acid coevolution in an ensemble of sequences comprising a protein family. This approach indicates a functional architecture within proteins in which the basic units are coupled networks of amino acids termed sectors. This evolution-based decomposition has potential for new understandings of the structural basis for protein function. To facilitate its usage, we present here the principles and practice of the SCA and introduce new methods for sector analysis in a python-based software package (pySCA). We show that the pattern of amino acid interactions within sectors is linked to the divergence of functional lineages in a multiple sequence alignment—a model for how sector properties might be differentially tuned in members of a protein family. This work provides new tools for studying proteins and for generally testing the concept of sectors as the principal units of function and adaptive variation.

## Author Summary

Proteins display the ability to fold, to carry out complex biochemical reactions, and to be adaptive to changing conditions of selection—the essential characteristics contributing to organismal fitness. A major goal is to understand how these properties emerge from the global pattern of interactions between amino acid residues. Here, we describe the principles and implementation of the statistical coupling analysis (SCA), a method to reveal this pattern through analysis of coevolution between amino acids in an ensemble of homologous sequences. The basic result is a decomposition of protein structures into groups of contiguous amino acids called “sectors” which have been linked to conserved functional properties. This work provides conceptual and practical tools for sector analysis in any sufficiently well-represented protein family, and represents a necessary basis for broadly testing the concept of protein sectors.

## Introduction

The amino acid sequence of a protein reflects the selective constraints underlying its fitness and, more generally, the evolutionary history that led to its formation [1]. A central problem is to decode this information from the sequence, and thus understand both the “architecture” of natural proteins, and the process by which they evolve. With the dramatic expansion of the sequence databases, a powerful strategy is to carry out statistical analyses of the evolutionary record of a protein family [2–6]. With the assumption that the principal constraints underlying folding, function, and other aspects of fitness are conserved during evolution, the idea is to start with an ensemble of homologous sequences, make a multiple sequence alignment, and compute a matrix of correlations between sequence positions—the expected statistical signature of couplings between amino acids. Using mathematical analyses that explore different aspects of this matrix [7, 8], studies have exposed tertiary structural contacts in protein structures (Direct Coupling Analysis, or DCA, [4, 9]), determinants of binding specificity in paralogous protein complexes [5], and larger, collectively evolving functional networks of amino acids termed “protein sectors” (Statistical Coupling Analysis, or SCA [10]. These different approaches suggest a hierarchy of information contained in protein sequences that ranges from local constraints that come from direct contacts between amino acids in protein structures to global constraints that come from the cooperative action of many amino acids distributed through the protein structure. Sectors are interesting since they may represent the structural basis for functional properties such as signal transmission within [3, 6, 11–14] and between [15–17] proteins, allosteric regulation [6, 15, 18–20], the collective dynamics associated with catalytic reactions [16], and the capacity of proteins to adapt [21]. In addition, experiments show that reconstituting sectors is sufficient to build artificial proteins that fold and function in a manner similar to their natural counterparts [22–24]. Thus, the quantitative analysis of coevolution provides a powerful approach for generating new hypotheses about the physics and evolution of protein folding and function.

These results imply that together with structure determination and functional measurements, the evolution-based decomposition of proteins should be a routine process in our study of proteins. However, the analysis of coevolution poses non-trivial challenges, both conceptually and technically. Conceptually, coevolution is the statistical consequence of the cooperative contribution of amino acid positions to organismal fitness, a property whose relationship to known structural or biochemical properties of proteins remains open for study. Indeed, there is no pre-existing model of physical couplings of amino acids with which to validate patterns of coevolution. Thus, the goal of coevolution based methods is to produce models for the pattern of constraints between amino acids that can then be experimentally tested for structural, biochemical, and evolutionary meaning. Technically, the analysis of coevolution is complicated by both the limited and biased sampling of sequences comprising a protein family. Thus, empirical correlations deduced from multiple sequence alignments do not always reflect coevolution. Interestingly, the complexities in sequence sampling can represent both sources of noise and useful signal in decomposing protein structures, and it is essential to understand these issues in effectively using methods of coevolution.

The DCA approach for mapping amino acid contacts has been well-described by analogy with established theory in statistical physics [4]. Here, we present the principles and implementation of the SCA method for identifying sectors and introduce new tools for understanding the global patterns of coevolution between amino acid positions. The methods are implemented in an open-source python-based software package that is available to the scientific community, and illustrated in the main text using the small G protein family of nucleotide-dependent switches [25, 26] and the S1A family of serine proteases [27, 28]. Technical modifications from previous implementations of SCA are indicated in the main text and summarized in the the S1 Table. In prior work, we examined just the broadest level of coevolution to define sectors—quasi-independent groups of coevolving amino acids [10]. We now go beyond this top-level decomposition to reveal a more elaborate internal architecture for sectors in which subgroups of amino acids diverge along functional, and sometimes phylogenetic, subfamilies within the sequence alignment. Overall, this work provides a necessary foundation for broad testing of the concept of protein sectors.

## Results

**Fig 1:**
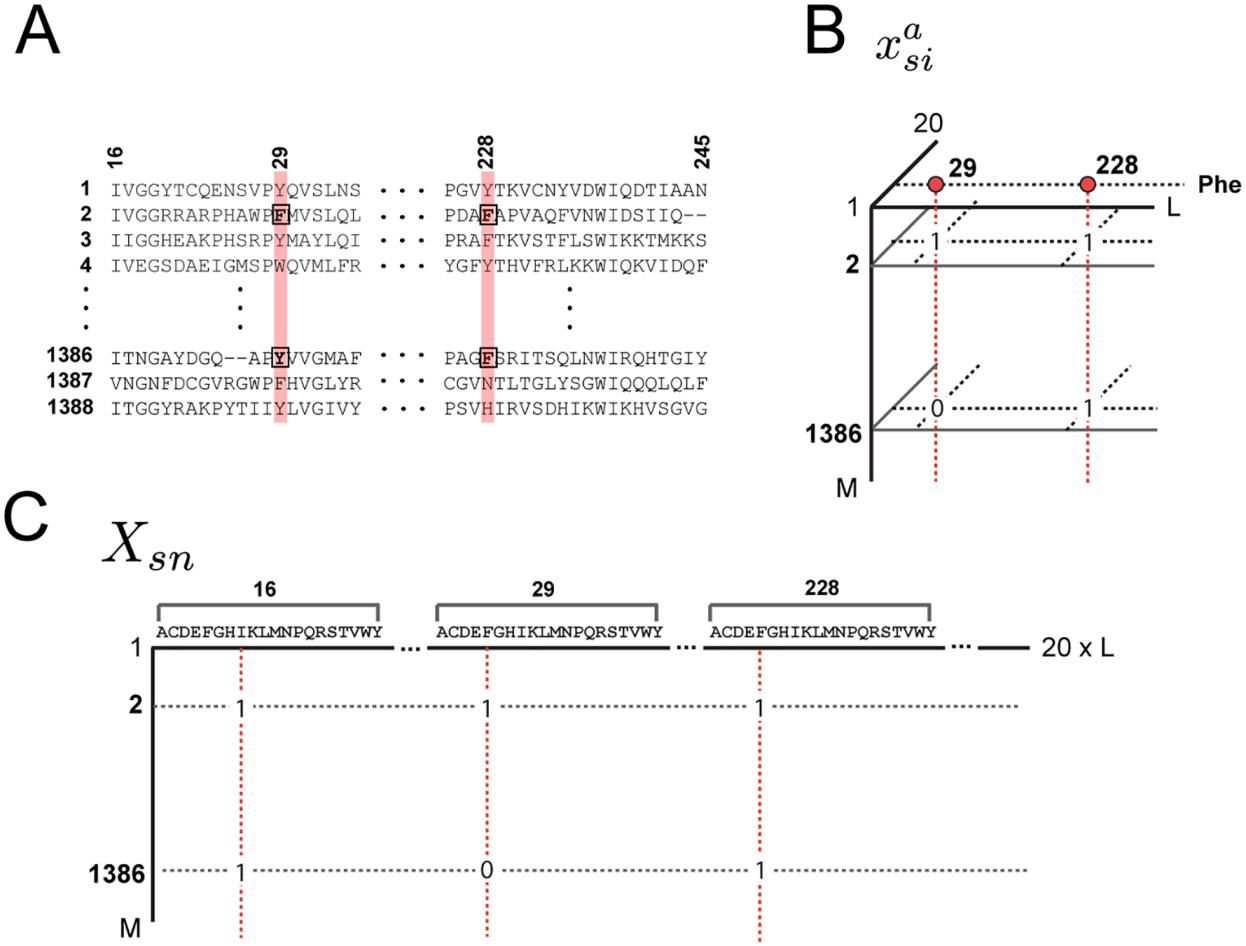
Three representations of a multiple sequence alignment comprised of *M* sequences and *L* positions. **A**, ascii text. **B**, a three-dimensional binary array $x_{si}^{a}$, in which $x_{si}^{a}=1$ if sequence *s* has amino acid *a* at position *i*, and 0 otherwise; gaps are always set to 0. In this representation, the frequencies of amino acids at individual positions are $f_{i}^{a}={\langle x_{si}^{a}\rangle }_{s}\equiv \sum_{s}w_{s}x_{si}^{a}/M^{\prime }$, where *w*_*s*_ is the weight for each sequence *s* and *M*′ = ∑_*s*_ *w*_*s*_ represents the effective number of sequences in the alignment. Joint frequencies of amino acids between pairs of positions are defined by $f_{ij}^{ab}={\langle x_{si}^{a}x_{sj}^{b}\rangle }_{s}\equiv \sum_{s}w_{s}x_{si}^{a}x_{sj}^{b}/M^{\prime }$. **C**, a two-dimensional alignment matrix *X*_*sn*_, in which the index *s* (along rows) represents sequences and the index *n* (along columns) represents the combination of amino acid and position dimensions in one, such that *n* = 20(*i* − 1) + *a*. This representation is useful in explaining the relationship between patterns of coevolution between amino acids and patterns of sequence divergence in the protein family (see Eq (12)).

The SCA begins with an alignment (*M* sequences by *L* positions) representing a sampling of homologous sequences expected to share common selective pressures (Fig 1 and S1 Text, section A). Standard sequence database searching algorithms (BLAST, PSI-BLAST, etc.) [29] together with automated alignment tools (e.g. PROMALS [30]), or available databases of multiple sequence alignments such as PFAM [31] seem to provide suitable sequence alignments. Since SCA concentrates on conserved features of protein sequences (see below), it is relatively robust to variations in alignment quality, but will depend on the depth and diversity of sampling of homologous sequences. While an alignment of a protein family is in principle sufficient for an analysis of coevolution, taxonomic and functional annotations and atomic structures are valuable for interpretation. In this work, we will assume that an atomic structure is known for at least one sequence in the alignment. We also assume that the alignment has been subject to a number of pre-processing steps in which positions and sequences with too many gaps are removed, and a simple sequence-weighting scheme [4] is applied to correct for the trivial over-representation of high-identity sequences. Each sequence *s* is given a weight $w_{s}=\frac{1}{N_{s}}$, where *N*_*s*_ is the number of sequences with an identity to *s* above a specified threshold (80% by default, Box 1). With sequence weights, we can compute an effective number of sequences in the alignment *M*′ = ∑_*s*_ *w*_*s*_ where *w*_*s*_ is the weight for sequence *s*. For computational efficiency, the alignment is then down-sampled to a limit that preserves *M*′. More advanced methods for treating sequence relationships are possible [32] and will require further study.

**BOX 1: A SUMMARY OF CALCULATIONS**

**Alignment preprocessing:** An alignment is represented by a *M* × *L* × 20 binary array $x_{si}^{a}$ where *s* = 1, …, *M* labels the sequences, *i* = 1, …, *L* the positions, *a* = 1, …, 20 the amino acids, with $x_{si}^{a}=1$ if sequence *s* has amino acid *a* at position *i* and 0 otherwise. Preprocessing steps:

1. Truncate excessively gapped positions based on a reference sequence or by a specified gap fraction cutoff (default, 0.4);
2. Remove sequences with a fraction of gaps greater than a specified value *γ*_seq_ (default, *γ*_seq_ = 0.2);
3. Remove sequences *r* with *S*_*r*_ < Δ, where *S*_*r*_ if the fractional identity between *r* and a specified reference sequence (default, Δ = 0.2);
4. Compute sequence weights *w*_*s*_ = 1/|{*r*: *S*_*rs*_ > *δ*}| where *S*_*rs*_ if the fractional identity between *r* and *s* (default, *δ* = 0.8), and truncate positions *i* with a frequency of gaps $f_{i}^{0}=1-\sum_{s,a}w_{s}x_{si}^{a}/\sum_{s}w_{s}$ greater than a specified value *γ*_pos_ (default, *γ*_pos_ = 0.2);
5. Recompute the sequence weights *w*_*s*_ for the truncated alignment, and compute the frequencies of amino acid at individual positions *i* as $f_{i}^{a}=\left(1-\lambda \right)\sum_{s}w_{s}x_{si}^{a}/M^{\prime }+\lambda /21$, and at pairs of positions *ij* as $f_{ij}^{ab}=\left(1-\lambda \right)\sum_{s}w_{s}x_{si}^{a}x_{sj}^{b}/M^{\prime }+\lambda /{\left(21\right)}^{2}$, where *M*′ = ∑_*s*_ *w*_*s*_ represents the effective number of sequences in the alignment and where *λ* is a small regularization parameter (default, *λ* = 0.03).
  When dealing with large alignments, a sixth step may be added to speed up the subsequent calculations:
6. Resample *M*′′ sequences, with *M*′ < *M*′′ < *M*, by drawing them randomly from the original alignment with weights *w*_*s*_ so as to form an alignment with a smaller number of sequences but an equivalent effective number of sequences (which may slightly exceed *M*′, see SI; default, *M*′′ = 1.5 × *M*′).

**Structure of evolutionary conservation:** For a large and diverse alignment (*M*′ > 100, minimally), the evolutionary conservation of each amino acid *a* at position *i* taken independently of other positions is measured by the statistical quantity $D_{i}^{a}$, the Kullback-Leibler relative entropy of $f_{i}^{a}$ given *q*^*a*^, the background distribution of amino acids:

$$D_{i}^{a}=f_{i}^{a}\text{ln}\frac{f_{i}^{a}}{q^{a}}+\left(1-f_{i}^{a}\right)\text{ln}\frac{1-f_{i}^{a}}{1-q^{a}}.$$ (1)

*q* is computed over the non-redundant database of protein sequences. If gaps are considered, and ${\bar{q}}^{0}$ represents the fraction of gaps in the alignment, a background frequency for gaps can be taken as ${\bar{q}}^{0}$, and then ${\bar{q}}^{a}=\left(1-{\bar{q}}^{0}\right)q^{a}$ for the 20 amino acids. Also, $D_{i}=\sum_{a=0}^{20}f_{i}^{a}\text{ln}(f_{i}^{a}/{\bar{q}}^{a})$ defines the overall conservation of position *i* taking all amino acids into account. To examine the co-evolution of pairs of amino acids, we introduce a measure that reports the significance of the raw correlations, $C_{ij}^{ab}=f_{ij}^{ab}-f_{i}^{a}f_{j}^{b}$, as judged by the degree of conservation of the underlying amino acids:

$${\tilde{C}}_{ij}^{ab}=\phi_{i}^{a}\phi_{j}^{b}C_{ij}^{ab},\;\text{in}\;\text{which}\;\phi_{i}^{a}=\frac{\partial D_{i}^{a}}{\partial f_{i}^{a}}=\text{ln}\left[\frac{f_{i}^{a}\left(1-q^{a}\right)}{\left(1-f_{i}^{a}\right)q^{a}}\right].$$ (2)

The information in the amino acid correlation matrix for each pair of positions is compressed into one number by computing the “Frobenius norm” of the 20 × 20 matrix ${\tilde{C}}_{ij}^{ab}$ for each (*ij*):

$${\tilde{C}}_{ij}=\sqrt{\sum_{a,b}{\left({\tilde{C}}_{ij}^{ab}\right)}^{2}}.$$ (3)

Analysis of ${\tilde{C}}_{ij}$ involves (a) spectral (or eigenvalue) decomposition of ${\tilde{C}}_{ij}$, given by $\tilde{C}=\tilde{V}\tilde{\Delta }{\tilde{V}}^{⊤}$, (b) determination of k* significant eigenvalues (by comparison with vertically randomized alignments), (c) a transformation of the top k* eigenvectors by independent components analysis (ICA), and (d) study of the pattern of residue contributions along independent components (ICs) 1…k*. Distinct groups of positions can emerge along the ICs for two reasons: (1) the existence of multiple independent sectors, or (2) the hierarchical breakdown of one sector into subgroups that arise from heterogeneities in the alignment.

**Mapping and sector interpretation:** The singular value decomposition (SVD) of the 20 × 20 matrix ${\tilde{C}}_{ij}^{ab}$ for each (*ij*), ${\tilde{C}}_{ij}^{ab}=\sum_{c=1}^{20}P_{ij}^{ac}\lambda_{ij}^{c}Q_{ij}^{cb}$, has the property that $\lambda_{ij}^{1}\gg \lambda_{ij}^{c}$ for *c* ≠ 1 (S1 Fig). That is, the information in the amino acid correlation matrix for each pair of positions can be compressed into one number, the top singular value (also known as the “spectral norm”). Besides compressibility, another empirical property of the SVD of ${\tilde{C}}_{ij}^{ab}$ is that for a given position *i*, the top singular vector $P_{ij}^{1a}$ is (up to the sign) nearly independent of *j* (S3 Fig). That is, the amino acids by which a position *i* makes correlations with other positions *j* is nearly the same, and therefore is essentially a property of just position *i* taken independently. This defines a projection matrix ${\bar{P}}_{i}^{a}=\phi_{i}^{a}f_{i}^{a}/{\left(\sum_{b}{\left(\phi_{i}^{b}f_{i}^{b}\right)}^{2}\right)}^{1/2}$ with which we can reduce the *M* × *L* × 20 array $x_{si}^{a}$ to an *M* × *L* alignment matrix $x_{si}=\sum_{a}{\bar{P}}_{i}^{a}x_{si}^{a}$. The matrix *x*_*si*_ gives a mapping between the space of positional correlations and the space of sequence correlations. Specifically, if $\tilde{\Delta }$ and $\tilde{V}$ are the eigenvalues and eigenvectors of ${\tilde{C}}_{ij}$, then

$$\tilde{U}=x\tilde{V}{\tilde{\Delta }}^{-\frac{1}{2}}$$ (4)

represents the structure of sequence space corresponding to the positional correlations in $\tilde{V}$. Also, if *W* is the transformation matrix derived from the ICA of $\tilde{V}$, then ${\tilde{U}}^{p}=W\tilde{U}$ represents the sequence space corresponding to the ICs of ${\tilde{C}}_{ij}$. This mapping between position and sequence space provides a method to study the origin of the hierarchical pattern of coevolution that underlies sectors.

In this work, we use a PFAM-based alignment of the G protein family (PFAM, PF00071, version 27.0), and an alignment of S1A serine proteases modified from Halabi et al. [10]. After the pre-processing steps with default values for thresholds, we obtain a final G protein sequence alignment of 4978 sequences by 158 positions (3364 effective sequences) and a final S1A alignment of 1344 sequences by 205 positions (928 effective sequences). In what follows, we will assume that sequence weights are applied and for simplicity we will simply denote *M*′ by *M*. No calculations below explicitly depend on its value; we shall only assume that *M*′ is large enough to give good estimates of amino acid frequencies (*M*′ > 100).

An interesting point is that for nearly all alignments, the number of “variables” (*L* × 20 possible amino acids) is typically on the order of or greater than the number of “samples” (*M*). Thus, it would seem impossible to reliably estimate the correlations between every pair of amino acids given such limited sampling. However, the sparsity of the constraints between amino acids observed both statistically [6, 10] and experimentally [33–35] effectively reduces the dimensionality of the solution, enabling practical approaches. The key issue is to propose a general approach for recognizing the “basis”—or groups of relevant amino acid positions—in which this solution largely exists. In SCA, the approach is to weight correlations by the degree of conservation of amino acids with the intuition that this fundamentally defines the relevance of features emerging from an evolutionary process. We develop this approach by first defining the first-order conservation of positions taken independently and then extending to correlations between positions.

### First-order statistics: Position-specific conservation

**Fig 2:**
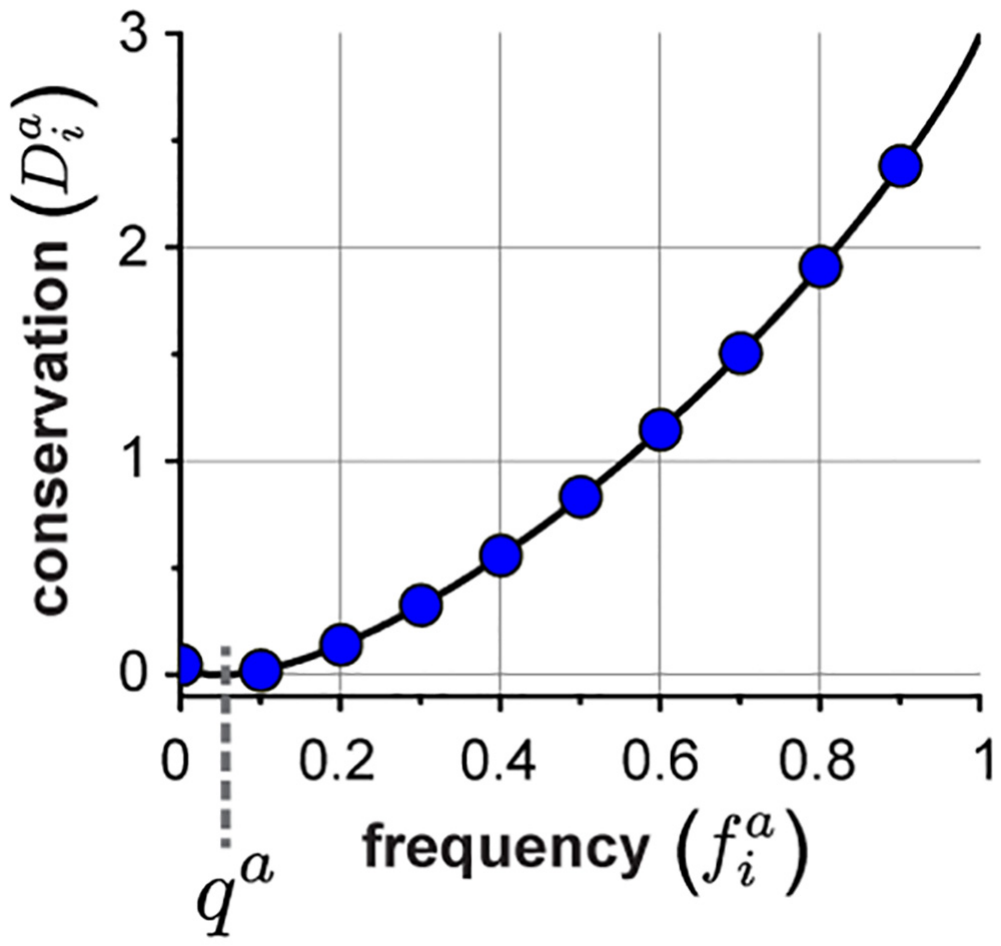
$D_{i}^{a}$, the measure of amino acid conservation. **A**, A plot of $D_{i}^{a}$ as a function of $f_{i}^{a}$, the amino acid frequency, and *q*^*a*^, the background frequency here for illustration set to 0.05. See the Supplementary Information for actual values of *q*.

**Fig 3:**
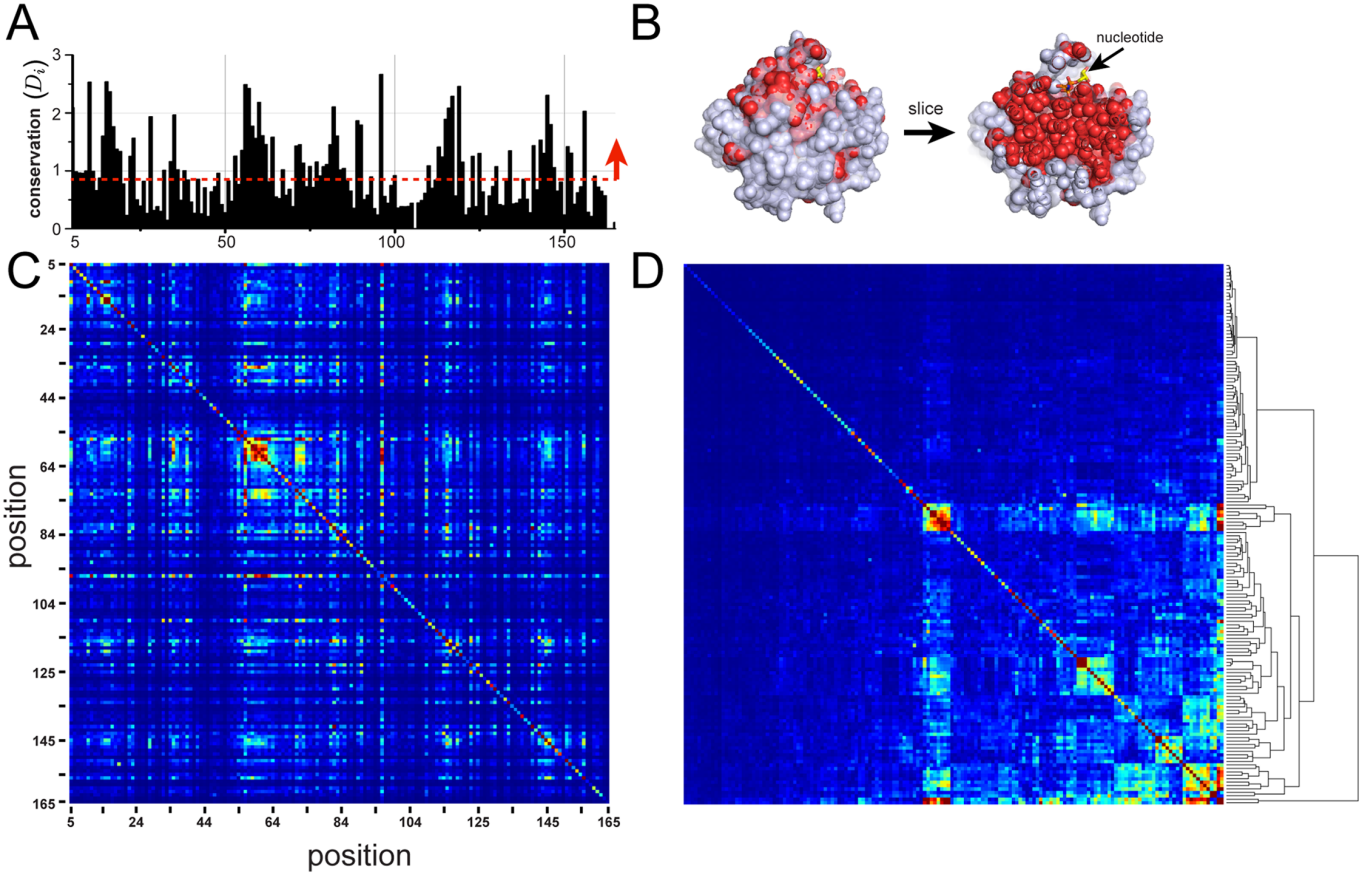
Positional conservation (*D*_*i*_) and the SCA weighted correlation matrix ${\tilde{C}}_{ij}$ for the G protein family. **A-B**, The overall positional conservation *D*_*i*_ for the G protein alignment, and a corresponding mapping on a slice through the core of the atomic structure of a representative member of the family (human Ras, PDB 5P21). The data show that the top 50% of conserved positions (in red) lie at functional surfaces and within the solvent inaccessible core. Thus, positional conservation maps to an intuitive and a well-known decomposition of protein structures. **C-D**, ${\tilde{C}}_{ij}$ ordered by primary structure (**C**), and after hierarchical clustering (**D**). The data describe a sparse and seemingly hierarchical organization of correlations—a general result for most protein families.

The evolutionary conservation of a sequence position is estimated from the deviation of the observed distribution of amino acids at this position from a background distribution expected by neutral drift. A simple mathematical quantity that captures this concept is

$$D_{i}^{a}=f_{i}^{a}\text{ln}\frac{f_{i}^{a}}{q^{a}}+\left(1-f_{i}^{a}\right)\text{ln}\frac{1-f_{i}^{a}}{1-q^{a}},$$ (5)

where $f_{i}^{a}$ is the observed frequency of amino acid *a* at position *i* in the alignment and *q*^*a*^ is the background expectation (see S1 Text, section B for derivation). $D_{i}^{a}$ is known as the Kullback-Leibler relative entropy [36] and indicates how unlikely the observed frequency of amino acid *a* at position *i* would be if *a* occurred randomly with probability *q*^*a*^—a quantitative measure of position-specific conservation. Note that $D_{i}^{a}=0$ only when $f_{i}^{a}=q^{a}$ and increases more and more steeply as $f_{i}^{a}$ deviates from *q*^*a*^ (Fig 2), consistent with intuition that a measure of conservation should non-linearly describe the divergence of the observed distribution of amino acids from their expected values. An underlying assumption in the derivation of the relative entropy is that the sampling of sequences in the alignment is unbiased, a condition that, to varying extent, is violated by the tree-like phylogenetic structure of real alignments. But without validated models for protein evolution that can provide a basis for more accurate measures of conservation, this choice reflects the simplest definition that satisfies the general principle of conservation. Finally, Eq (5) gives the conservation of each amino acid *a* at each position *i*, but an overall positional conservation *D*_*i*_ can be defined following the same principles (Fig 3A, and see S1 Text, section C).

Analysis of the spatial pattern of positional conservation generally leads to a simple conclusion: the solvent inaccessible core of proteins and functional surfaces tend to be more conserved and the remainder of the surface is less conserved (Fig 3A and 3B) [10, 37, 38]. Thus, positional conservation in sequence alignments reflects well-known properties of protein three-dimensional structures.

### Second-order statistics: Conserved correlations

The cooperativity of amino acids in specifying protein folding and function implies that the concept of positional conservation of individual positions should at least be extended to a concept of pairwise conservation, reporting coevolution between positions in a protein family. Given the alignment, a measure of correlation of the pair of amino acids (*a*, *b*) at positions (*i*, *j*) is given by the difference of their joint frequency $f_{ij}^{ab}$ and that expected in absence of correlation, $f_{i}^{a}f_{j}^{b}$. Computed for all pairs of amino acids in the alignment, this defines a covariance matrix

$$C_{ij}^{ab}=f_{ij}^{ab}-f_{i}^{a}f_{j}^{b}.$$ (6)

Alternatively, statistical dependency can be quantified by the mutual information, whose origin is similar to the relative entropy [5, 36]. However, both the covariance matrix and the mutual information report deviations from independence given the frequencies $f_{i}^{a}$, and do not take into account the evolutionary relevance of observing those frequencies. In the current implementation of SCA, the approach is to perform a first-order perturbation analysis on the multiple sequence alignment in which we compute the correlated conservation of pairs of amino acids. To explain, consider that many alignments *A* are available for the same protein family. We can then define relative entropies $D_{i,A}^{a}$—our measure of positional conservation—for each alignment *A*, and compute their correlations over the ensemble of alignments by

$${\tilde{C}}_{ij}^{ab}={\left\langle D_{i,A}^{a}D_{j,A}^{b}\right\rangle }_{A}-{\left\langle D_{i,A}^{a}\right\rangle }_{A}{\left\langle D_{j,A}^{b}\right\rangle }_{A},$$ (7)

where the angled brackets indicate averages over the *A* alignments. In practice, many such alignments can be obtained by bootstrap resampling the original alignment [39]; for instance, a procedure known as “jackknife resampling” consists of successively removing each sequence *s* from the original alignment to create a collection of *M* sub-alignments. A perturbative expansion of $D_{i}^{a}$ as a function of $f_{i}^{a}$ for the jackknife resampling process shows that Eq (7) yields a covariance matrix that has the form

$${\tilde{C}}_{ij}^{ab}=\phi_{i}^{a}\phi_{j}^{b}C_{ij}^{ab},$$ (8)

in which $\phi_{i}^{a}=\frac{\partial D_{i}^{a}}{\partial f_{i}^{a}}$ is a function of the conservation of each amino acid at each position (see S1 Text, section D for derivation) [10]. That is, SCA produces a weighted covariance matrix, with the weighting function *ϕ* controlling the degree of emphasis on conservation. This definition of *ϕ* has the property of rising steeply as the frequencies of amino acids $f_{i}^{a}$ approach one. As a consequence, these weights damp correlations in $C_{ij}^{ab}$ arising from weakly conserved amino acids (the gradient of $D_{i}^{a}$ approaches zero as $f_{i}^{a}\to q^{a}$), and emphasize conserved correlations.

Another way to understand these weights comes from considering their role in determining similarities between sequences comprising the alignment. The mathematical principles are described below, but in essence positional weights $\phi_{i}^{a}$ redefine the distance between sequences in a manner that emphasizes variation at more conserved positions in the alignment (see S1 Text, section I). It is logical that such a “conservation-biased” distance metric between sequences will provide a better representation of the functional differences (as opposed to historical differences) between sequences. The weighting by $\phi_{i}^{a}$ in Eq (8) implements the same principle applied to the correlations between positions instead of the correlations between sequences.

In principle, the specific form of *ϕ* should vary depending on the evolutionary history of the protein properties that are under consideration; the more conserved the properties of interest are, the more the weights should emphasize conservation [40]. Indeed, different weighting functions are possible if mathematical formalisms other than the KL entropy are proposed for defining positional conservation, or if other approaches than the first-order perturbation analysis described here are developed. For example, early versions of the SCA method [3, 6] involved slightly different weights whose technical origins are given in S1 Text, section E.

${\tilde{C}}_{ij}^{ab}$ is a four-dimensional array of *L* positions × *L* positions × 20 amino acids × 20 amino acids, but we can compress it into a *L* × *L* matrix of positional correlations by taking a magnitude (the Frobenius norm) of each 20 × 20 amino acid coevolution matrix for each pair of positions (*i*, *j*):

$${\tilde{C}}_{ij}=\sqrt{\sum_{a,b}{\left({\tilde{C}}_{ij}^{ab}\right)}^{2}}$$ (9)

See S1 Text, section F and S1 Fig for additional arguments about compressibility of ${\tilde{C}}_{ij}^{ab}$.

Fig 3C and 3D shows the ${\tilde{C}}_{ij}$ matrix for the G protein family. As previously reported, the matrix is heterogeneous, with a small number of positions engaged in significantly higher correlations than most positions (Fig 3C, [6, 10]). Hierarchical clustering makes this heterogeneity more apparent, and reveals the existence of nested clusters of correlated positions (Fig 3D). These findings are qualitatively consistent with a sparse, hierarchical, and cooperative pattern of evolutionary constraints. As we show below, there is also modularity [10], with quasi-independent groups of positions emerging from the correlations (the sectors and their subdivisions). Unlike the interpretation of first-order conservation (Fig 3A and 3B), none of these properties is obvious in either current analyses of protein structures.

### Decomposition of the SCA positional correlation matrix

How can we understand the pattern of coevolution in the ${\tilde{C}}_{ij}$ matrix? The existence of correlations(Fig 3C) indicates that treating the amino acid positions as the basic units of proteins is not the most relevant representation. Instead, we should seek a transformation that re-parameterizes the protein into groups of correlated positions that are maximally independent from each other—a more natural representation that defines the units of evolutionary selection. The first step in this process is spectral (or eigenvalue) decomposition. Per this decomposition, the ${\tilde{C}}_{ij}$ matrix is written as a product of three matrices:

$$\tilde{C}=\tilde{V}\tilde{\Delta }\tilde{V^{⊤}},$$ (10)

where $\tilde{\Delta }$ is an *L* × *L* diagonal matrix of eigenvalues (ranked by magnitude) and $\tilde{V}$ is an *L* × *L* matrix whose columns contain the associated eigenvectors. Each eigenvalue gives the quantity of information (variance) in ${\tilde{C}}_{ij}$ captured, and each associated eigenvector in $\tilde{V}$ gives the weights for combining sequence positions into transformed variables (or eigenmodes).

**Fig 4:**
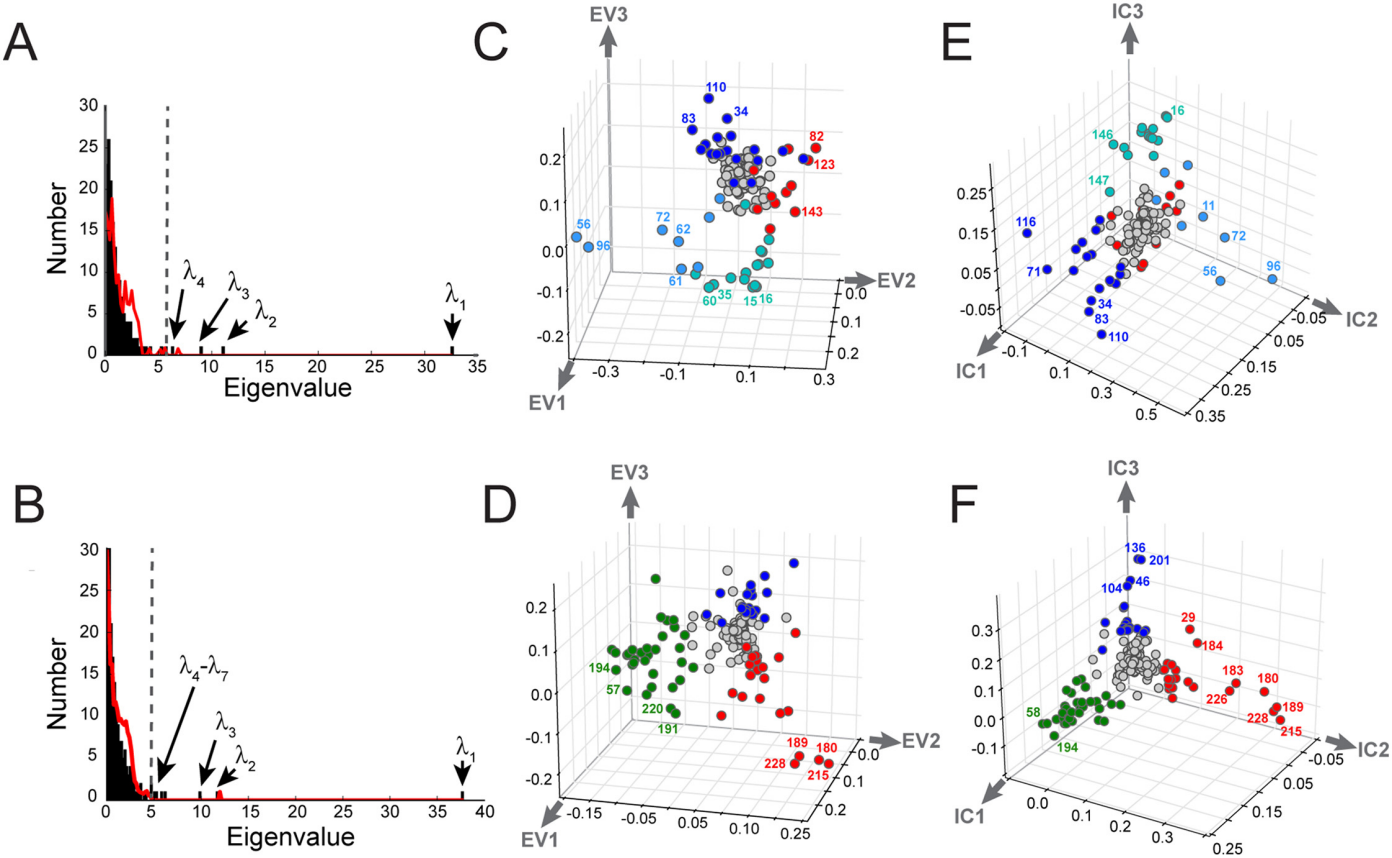
Spectral decomposition and ICA. **A-B**, The eigenspectrum of ${\tilde{C}}_{ij}$ (in black bars) for the G protein (**A**) and S1A (**B**) protein families. The eigenvalue distribution expected randomly is shown in red and provides a statistical basis for defining the k* top eigenmodes for further analysis—conservatively, those greater than the second random eigenvalue. The first random eigenvalue is ignored since it is a trivial consequence of retaining the independent conservation of sites in the randomization process [10]. This analysis suggests k* = 4 and k* = 7 for the G and S1A families, respectively. **C-D**, The top three eigenvectors for the G (**C**) and S1A (**D**) families suggest the possibility of distinct groups of coevolving positions, but illustrates the property that these groups emerge along combinations of eigenmodes. **E-F**, Independent components analysis (ICA) optimizes the independence of groups emerging along the different directions, putting the top three groups of amino acids on nearly orthogonal axes. The group of positions contributing to each IC groups is defined by fitting an empirical statistical distribution to the ICs and choosing positions above a defined cutoff (default, > 95% of the CDF). Groups of positions in panels **C-F** are defined and colored accordingly.

For both G protein and S1A alignments, the histogram of eigenvalues—the spectrum of ${\tilde{C}}_{ij}$—reveals a few large eigenvalues extending from a majority of small values (Fig 4A and 4B, black). To estimate the number of significant eigenvalues, we compare the actual spectrum with that for many trials of randomized alignments in which the amino acids at each position are scrambled independently [10] (Fig 4A and 4B, red line). This randomization removes true positional correlations, leaving behind the spurious correlations expected due to finite sampling in the alignment. As is the case for all practical alignments in which the number of effective sequences is not large compared to the number of amino acids, these spurious correlations account for the bulk of the spectrum. Indeed, for both alignments this analysis indicates that just the top few eigenmodes are statistically significant (k* = 4, G protein, and k* = 7, S1A; see S2 Fig for an analysis of robustness). Thus, the k* associated eigenvectors define a low dimensional space in which patterns of positional coevolution can be studied (e.g. Fig 4C and 4D). It is important to note that the precise value of k* is not a fundamental property of a protein family; it depends on protein size and the number of effective sequences. Nevertheless, with adequate sampling (*M*′ > 100) the analysis of sectors seems largely robust to its precise value (see DHFR tutorial, S3 Text).

Fig 4C and 4D show structure of the space spanned by the top three eigenvectors for the G protein and S1A families, respectively. In these graphs, the (Euclidean) distance of a position from the origin reports its overall contribution to the correlations, and the distance between two positions indicate their degree of correlation: strongly correlated positions appear near-by, while weakly correlated positions are far apart, or, for the majority that do not make any substantial contributions to the correlations, clustered near the origin. As a consequence, independent sets of correlated positions are expected to cluster into groups of positions at distance from the origin. When the correlations within these groups are organized hierarchically, these clusters extend radially with positions at extremity representing the core of the hierarchy, and successive layers at decreasing distance from the origin representing progressively weaker levels of the hierarchy. For both protein families, this analysis suggests a few distinct groups of positions that seem to emerge radially from the origin (Fig 4C and 4D, different colors).

The spectral decomposition is effective for dimension reduction, but the eigenmodes generally do not provide an optimal representation of groups of coevolving positions. For example, distinct groups of positions emerge along combinations of the k* top eigenvectors [10]. The reason is that just decorrelation of the positions by diagonalizing the ${\tilde{C}}_{ij}$ matrix—the essence of eigenvalue decomposition—is a weaker criterion than achieving statistical independence, which demands absence of not only pairwise correlations, but lack of any higher order statistical couplings. In prior work, we managed this problem heuristically, finding combinations of eigenmodes, excluding the first, that happen to represent quasi-independent groups [10]. Here, we introduce the use of independent components analysis (ICA [41, 42])—an extension of spectral decomposition—that computationally addresses this problem. ICA uses numerical optimization to deduce a matrix *W* that transforms the k* top eigenmodes of a correlation matrix into k* maximally independent components (ICs, S1 Text, section G),

$${\tilde{V}}_{1\cdots k^{*}}^{p}=W{\tilde{V}}_{1\cdots k^{*}}.$$ (11)

The bottom line is that the k* ICs (in columns of ${\tilde{V}}^{p}$) should now represent a more appropriate organization of positional coevolution.

In both G proteins (Fig 4E) and S1A proteases (Fig 4F), ICA produces a representation in which the majority of positions are weakly correlated and cluster near the origin and a relatively small subset of positions comprise quasi-independent groups of amino acids emerging along separate orthonormal axes (the ICs). The ICs need not be strictly independent, a key issue in defining sectors that we discuss in detail below. Nevertheless, spectral decomposition with ICA provides the sort of transformation of protein sequences that we seek—based on their evolutionary correlations, amino acid positions are regrouped and transformed into new effective variables (the ICs) that represent collectively evolving modes of the protein under study.

### Sequence-position mapping: An interpretation of the decomposition

How can we examine the relevance of the IC-based decomposition of proteins? A approach comes from understanding a fundamental mathematical relationship between the pattern of positional correlations (which defines ICs) and the structure of the sequence space spanned by the alignment (which defines sequence subfamilies) [43, 44]. The concepts underlying this mapping between positions and sequences were presented either heuristically [10] or partially [17] in prior work; here, we provide a full explanation with new mathematical methods.

**Fig 5:**
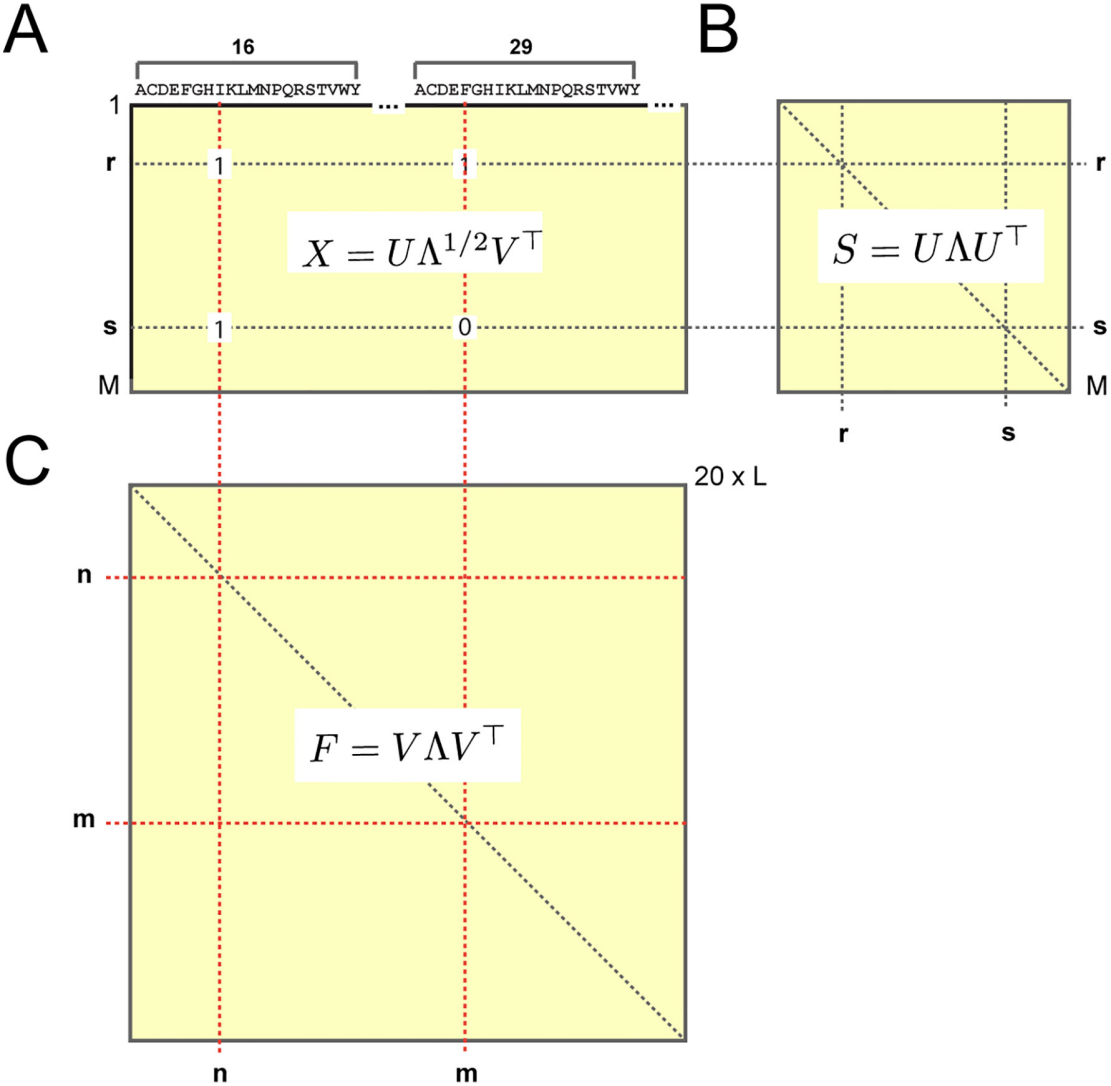
The mathematical relationship between sequence and positional correlations. **A**, A binary matrix representation of the alignment *X*_*sn*_, comprised of *M* sequences by 20 × *L* amino acids (Fig 1C); the equation shows the singular value decomposition (SVD) of *X* (Eq (12)). From the alignment matrix, two correlation matrices can be computed: *S*, a correlation matrix over rows (**B**) describing relationships between sequences, and *F*, a correlation matrix over columns (**C**) describing relationships between amino acids; equations show the eigenvalue decompositions of these matrices. By the SVD, *X* provides a mapping between the two such that the eigenvectors of *F* (in *V*) correspond to the eigenvectors of *S* (in *U*). Thus, it is possible to associate coevolving groups of amino acids to patterns of sequence divergence in the alignment. As described in the text, a similar mapping is possible for positional (rather than amino acid specific) coevolution (Eq (14)).

Consider the two-dimensional binary matrix representation of an alignment *X*_*sn*_ comprised of *M* sequences by 20*L* amino acids (Figs 1C and 5A). From *X*_*sn*_, we can compute two kinds of correlations: (1) a correlation matrix over rows $S_{rs}=\frac{1}{L}\sum_{n}X_{rn}X_{sn}$, which represents the similarity (fraction identity) of each pair of sequences *r* and *s* (Fig 5B) and (2) a correlation matrix over columns $F_{nm}=\frac{1}{M}\sum_{s}X_{sn}X_{sm}$, which represents the joint frequency of amino acids at each pair of positions (Fig 5C). *F* and *S* are intimately related to each other by a mathematical property known as the singular value decomposition (SVD). Specifically, if *U* represents the eigenvectors of the sequence correlation matrix *S* and *V* represents the eigenvectors of the amino acid correlation matrix *F*, then

$$X=U\Lambda^{1/2}V^{⊤},$$ (12)

where Λ is a diagonal matrix whose entries are (up to a scaling factor) eigenvalues of both *S* and *F*. The key conceptual point is that by the SVD, the eigenvectors of *S* are a mapping from the eigenvectors of *F*, where the “map” is the alignment *X* itself,

$$U=XV\Lambda^{-1/2}.$$ (13)

This introduces the principle of sequence-position mapping, using the full alignment matrix *X* to relate patterns of amino acid correlations (in *V*) to patterns of sequence divergence (in *U*). But, to study the pattern of sequence divergence associated with sectors, we need to make a similar mapping using the conservation-weighted dimension-reduced coevolution matrix ${\tilde{C}}_{ij}$ (rather than the unweighted amino acid correlation matrix *F*). Since ${\tilde{C}}_{ij}$ is a *L* × *L* positional correlation matrix, a sequence-space mapping analogous to Eq (13) requires a dimension-reduced alignment matrix in which the 20 amino acids at each position are compressed into a single value. The Supplementary Information describes a new approach for this step, effectively reducing the alignment $x_{si}^{a}$ from a *M* × *L* × 20 array to an *M* × *L* matrix *x*_*si*_ by projecting the amino acid dimension down to a single scalar value (S1 Text, section H and S4 Fig). By analogy with Eq (13), the reduced alignment matrix *x*_*si*_ then defines a mapping between the space of positional coevolution (in the top ICs of the ${\tilde{C}}_{ij}$ matrix) and the corresponding sequence space. Specifically, if $\tilde{\Delta }$ and $\tilde{V}$ are the eigenvalues and eigenvectors, respectively, of the SCA positional coevolution matrix ${\tilde{C}}_{ij}$, then

$$\tilde{U}=x\tilde{V}{\tilde{\Delta }}^{-\frac{1}{2}}$$ (14)

represents the structure of the sequence space corresponding to the patterns of positional coevolution in $\tilde{V}$. Furthermore, if *W* is the transformation matrix derived from ICA of ${\tilde{V}}_{1\ldots k^{*}}$, Eq (11), then

$${\tilde{U}}^{p}=W\tilde{U}$$ (15)

represents the sequence space corresponding to ${\tilde{V}}^{p}$, the ICs of the ${\tilde{C}}_{ij}$ matrix.

**Fig 6:**
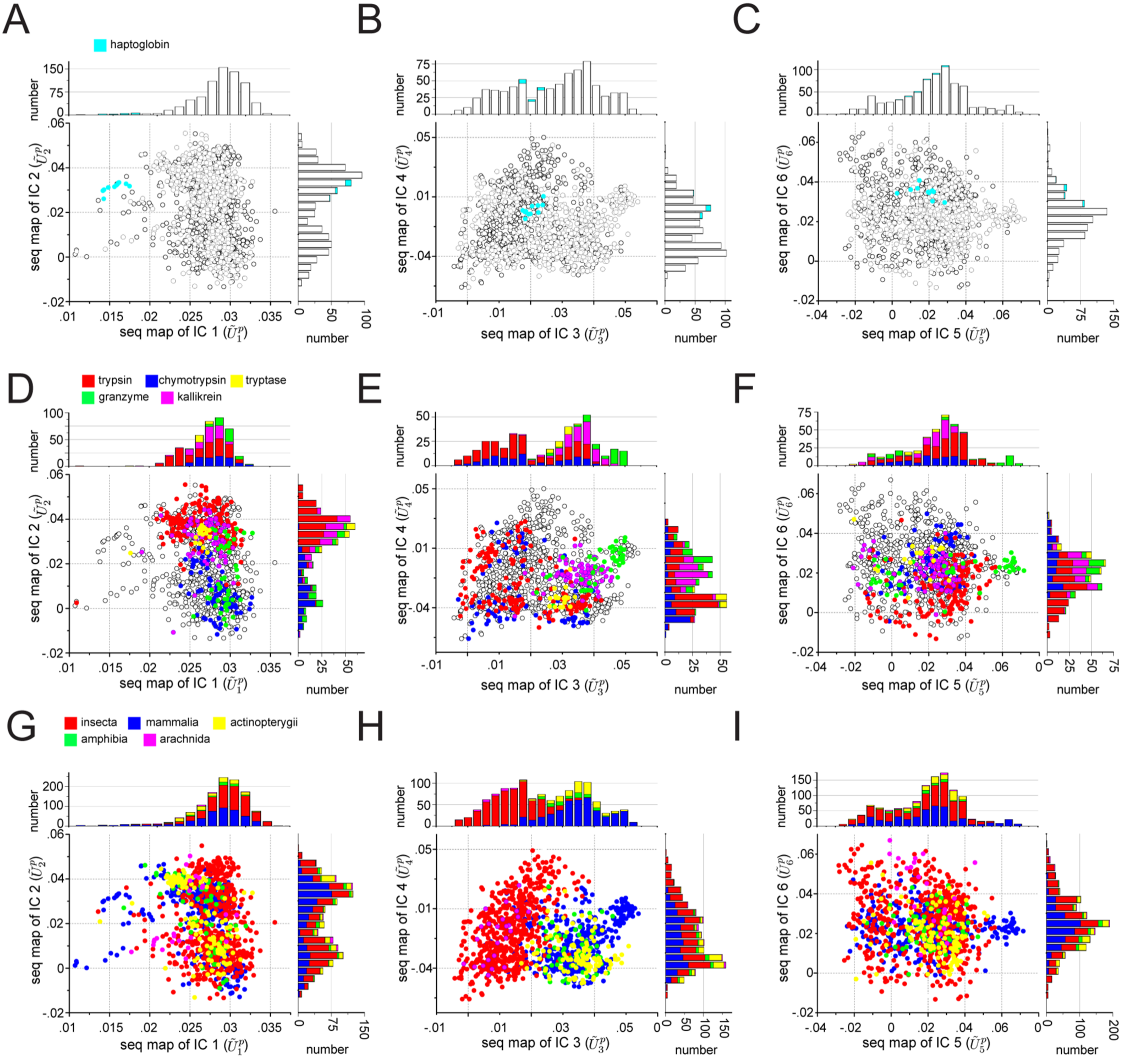
IC-based sequence divergences in the S1A protein family. The panels show scatterplots of sequences in the G protein alignment along dimensions (${\tilde{U}}_{1\ldots 6}^{p}$) that correspond to sequence variation in positions contributing to each of the top six ICs of the SCA coevolution matrix. The mapping between positional coevolution to sequence relationships is achieved using the reduced alignment matrix *x*, as per Eqs (14) and (15). Sequences are colored either by enzymatic activity (**A-C**, the haptoglobins are non-catalytic members of the S1A family), annotated catalytic specificity (**D-F**), or taxonomic origin (**G-I**). For each graph, the stacked histograms show the distributions of these classifications for each dimension. Note that trypsin, tryptase, kallikreins, and certain granzymes have tryptic specificity, and chymotrypsin and most granzymes have chymotryptic specificity. The data show that IC1 specifically separates sequences by enzymatic activity (**A**), IC2 separates sequences by catalytic specificity (**D**), IC3 separates sequences by invertebrate/vertebrate origin (**H**), and ICs 4–6 show more minor variations by catalytic specificity (**E-F**). These data (1) recapitulate and extend previous observations [10], and (2) demonstrate the functional relevance of the IC-based decomposition.

Eqs (14) and (15) give us the necessary tools for interpreting the IC-based decomposition of proteins. For the S1A family, Fig 6 shows a mapping between the top six ICs and the corresponding sequence space. Sequences are colored by enzymatic function (Fig 6A–6C, the haptoglobins are non-catalytic homologs of the S1A family), by catalytic specificity (Fig 6D–6F), or by phylogenetic origin (Fig 6G–6I). The data show that ICs 1–3 correspond to essentially orthogonal divergences in the S1A protein family. IC1 (but not any of the other ICs) separates the catalytic from non-catalytic S1A proteins (Fig 6A), IC2 uniquely separates S1A proteins by their annotated primary (P1 site) catalytic specificity [28] (Fig 6D), and IC3 uniquely separates vertebrate and invertebrate sequences (Fig 6H). ICs4–6 show more subtle inhomogeneities with regard to catalytic specificity (Fig 6E and 6F), indicating finer subdivisions of the annotated sequences—well-defined predictions for further study. Thus, the ICs of the ${\tilde{C}}_{ij}$ matrix contain independently evolving functional units within the S1A protease [10].

**Fig 7:**
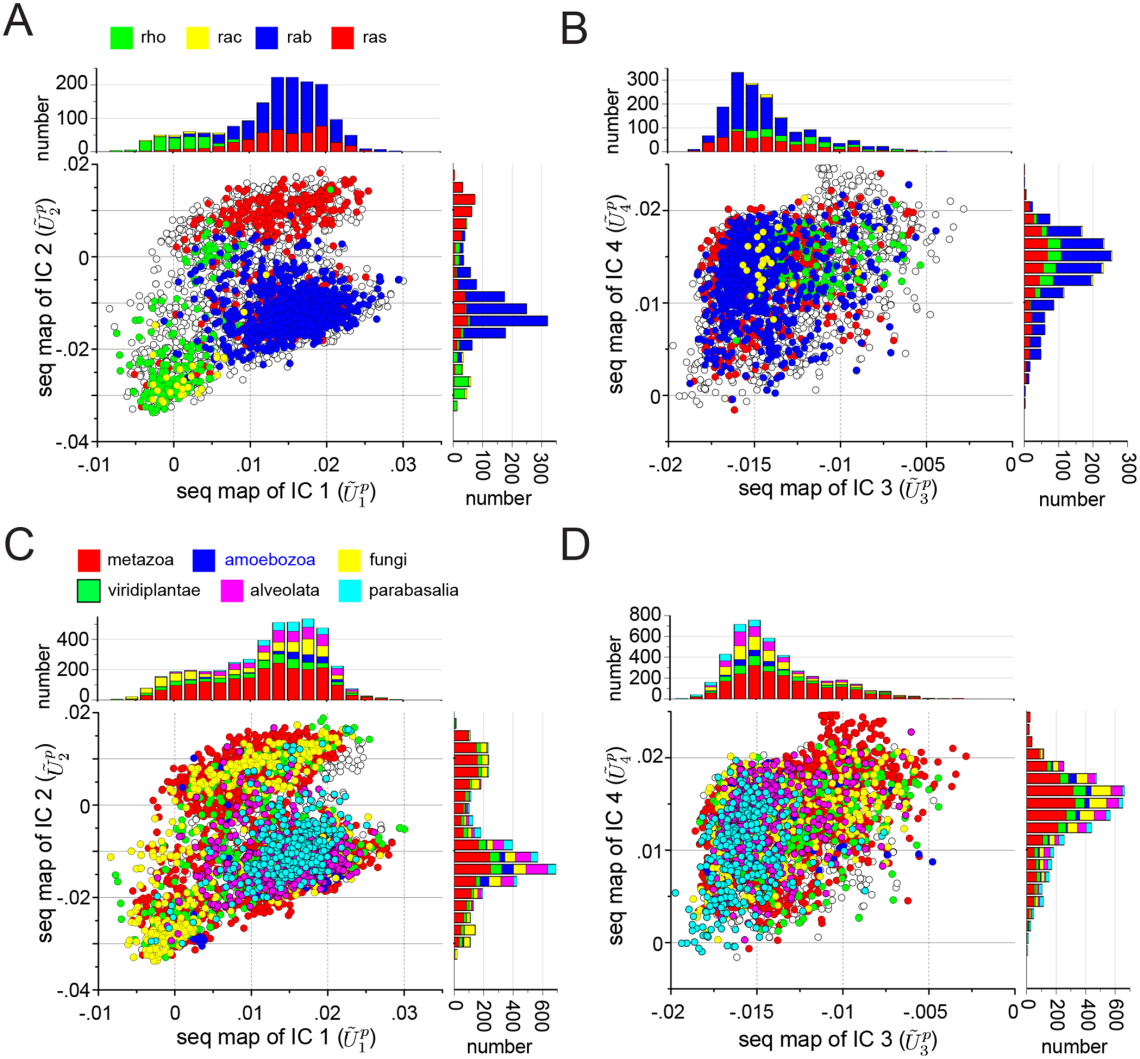
IC-based sequence divergences in the G protein family. The panels show scatterplots of sequences in the G protein alignment along dimensions (${\tilde{U}}_{1\ldots 4}^{p}$) that correspond to sequence variation in positions contributing to each of the four ICs of the SCA coevolution matrix. The mapping between positional coevolution to sequence relationships is achieved using the reduced alignment matrix *x*, as per Eqs (14) and (15). Sequences are colored either by annotated functional sub-type of G protein (**A-B**) or by taxonomic origin (**C-D**), and the stacked histograms show the distributions of these classifications for each dimension. The data show that ICs 1 and 2 (**A**) correspond to distinct sequence divergences of functional subtypes of G protein; for example, IC1 separates the Rho proteins (green) along ${\tilde{U}}_{1}^{p}$, and IC2 separates the Rho proteins (green) and a subset of Ras proteins (red) along ${\tilde{U}}_{2}^{p}$. In contrast, IC3 and IC4 are homogenous with regard to G protein subtype (**B**), and all ICs are essentially homogeneous with regard to phylogenetic divergence (**C-D**). These data suggest that IC3 and IC4 are nearly homogeneous features of the G protein family, while IC1 and IC2 are differentially selected for more specialized properties of G protein subtypes.

Fig 7A–7D shows the mapping between the top ICs of the G-protein family and the corresponding sequence space, colored either by functional sub-type (Fig 7A and 7B) or by taxonomic origin (Fig 7C and 7D). The data show that IC1 and IC2 separate different sub-classes of the G protein family, suggesting that like in S1A proteases, amino acid motifs in different ICs can control different functional properties (Fig 7A). In contrast, IC3 and IC4 are associated with a near homogeneous distribution of functional subtypes, suggesting either neutral or more fine variations with regard to the broad functional annotations available in this protein family (Fig 7B).

With the exception of IC3 in the S1A family (Fig 6H), none of the ICs are obviously associated with the divergence of the main taxonomic groups in the alignment; indeed, all taxa seem nearly homogeneously distributed over the sequence modes (*U*^*p*^) corresponding to most of the ICs. Many paralogs of the different functional classes of G proteins and S1A proteases are found in each type of organism and thus functional divergence might therefore not be expected to follow the divergence of species. In contrast, ICs are more associated with taxonomic classification for the DHFR protein family (S4 Fig and S3 Text), consistent with the fact that this core metabolic enzyme is encoded by a single ortholog in each genome.

**Fig 8:**
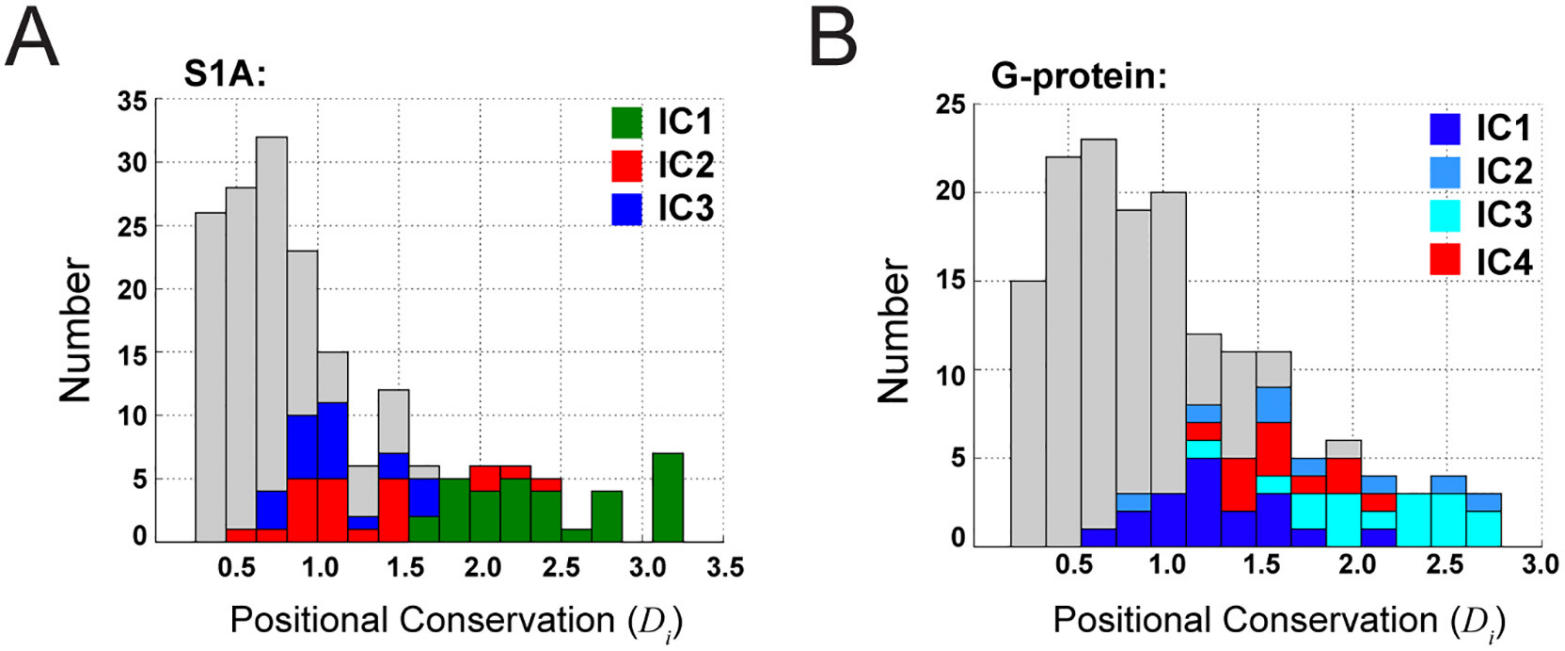
IC-based decomposition and positional conservation. Panels **A-B** show stacked histograms of positional conservation (*D*_*i*_) for the S1A and G protein families, respectively, with positions corresponding to different ICs marked in color as indicated. The data show that consistent with conservation-based weighting, positions contributing to the top ICs tend to be more conserved than average, but that the distinction between ICs cannot be made by just magnitude of positional conservation. Thus the IC-based decomposition of sequences is uniquely a property of analyzing correlations.

In summary, the sequence-position mapping provides evidence that the ICs of the ${\tilde{C}}_{ij}$ matrix represent conserved, differentially evolving functional units in proteins. The ICs are not distinguished by the magnitude of positional conservation (Fig 8), showing that this decomposition of proteins is fundamentally a property of correlations—the second order terms in conservation. This finding makes an important statement about the “value added” by studying coevolution, as opposed to just the first-order conservation of positions. Indeed, it is difficult to experimentally test the unique value of statistical coevolution by conventional single mutation experiments, even when conducted on a massive scale [21, 45, 46]. Coevolution implies the need for higher-order mutational studies, which are difficult to perform quantitatively and only now starting to become feasible [47]. In this regard, the functionally meaningful, quasi-independent divergence of proteins along ICs demonstrates the necessity of coevolution in providing a proper decomposition of protein structure.

### Sectors

But, does the existence of k* significant ICs imply k* independent functional units (and therefore k* sectors [10])? Not necessarily. Sectors typically have an organization in which the constituent positions can be further broken up into subsets of coevolving positions. One generative mechanism for this architecture comes from the tree-like structure of the alignment in which sequences are partitioned into functional subfamilies along which portions of one sector can diverge [43, 44]. Thus, each IC could have one of two interpretations: (1) a truly independent sector associated with a distinct function, or (2) the decomposition of a single sector (representing one functional property) into separately diverging sub-parts. In this sense, the term “independent component” is something of a misnomer, but we retain the language here for consistency with the ICA method.

**Fig 9:**
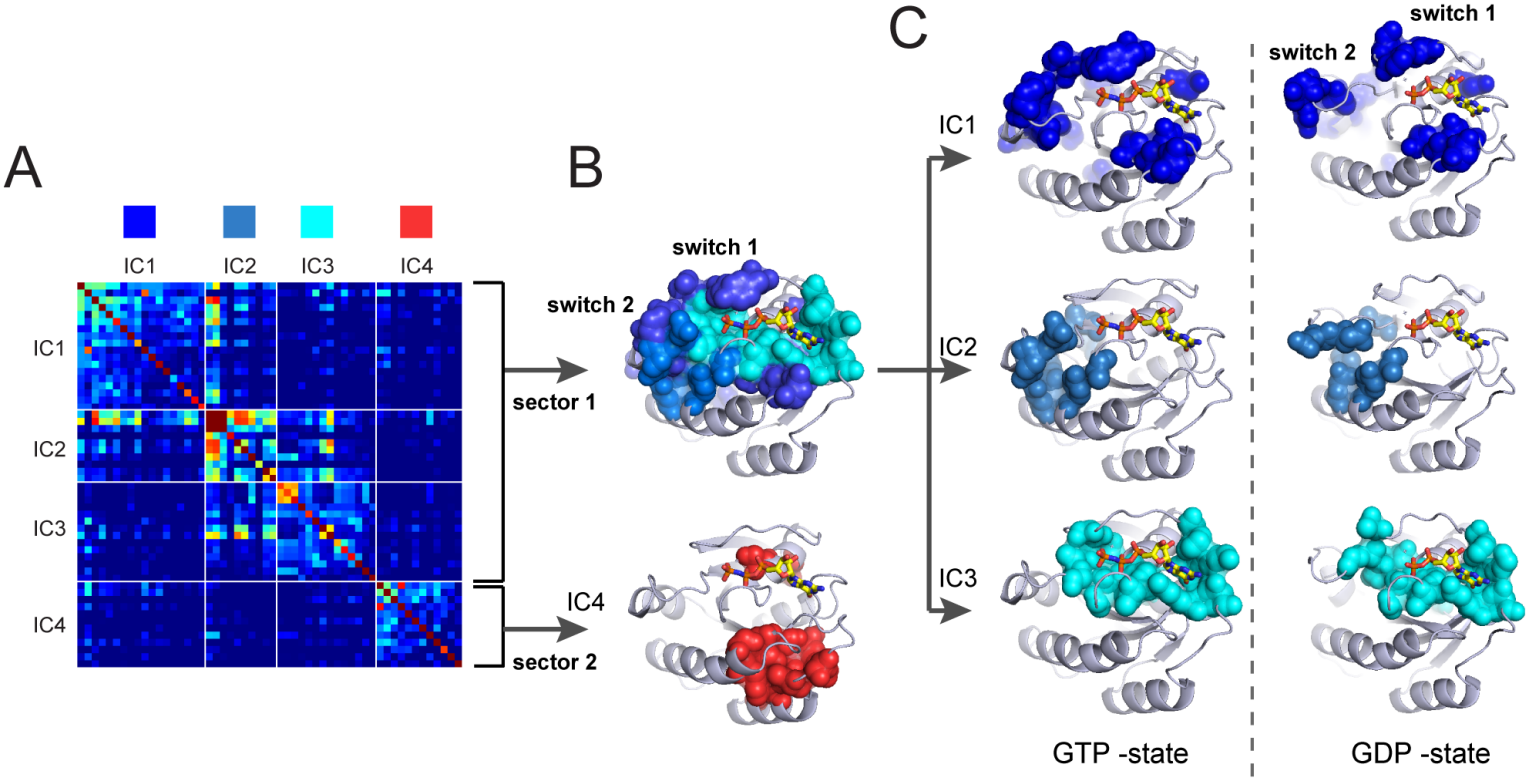
Sector identification for the G protein family. **A** shows the IC-based sub-matrix of the ${\tilde{C}}_{ij}$ matrix for the G protein family and and **B-C** shows the structural interpretations on a representative member of the family (H-Ras, PDB 5P21 [48]). IC4 represents a nearly independent group of coevolving positions (sector 2, red), while ICs 1, 2, and 3 show strong inter-IC correlations that suggest classification as a single hierarchically-organized sector (sector 1, different shades of blue). **B**, Structurally, sector 1 comprises the nucleotide binding pocket (IC1) and the connection to so-called switch domains 1 and 2 which interact with downstream target proteins (ICs 2 and 3). Together, these regions correspond to the known allosteric mechanism in the G protein family. Sector 2 corresponds to a distinct, largely contiguous group of amino acids with yet unclear functional role. **C**, The three ICs comprising sector 1 mapped on the atomic structures of the active GTP*γ*S bound state (PDB 5P21 [48], left panels) and inactive GDP-bound state (PDB 4Q21 [49], right panels) of H-ras. The data show that ICs 1 and 2 show substantial state-dependent conformational change. These same ICs also show distinct patterns of variation along different G protein sub-types (Fig 7A), suggesting that variations in these ICs tunes allosteric or substrate binding properties.

How can we systematically distinguish these possibilities to deduce the number and composition of sectors? We follow a simple procedure (see tutorials in S3 Text). First, we fit each IC to an empirical statistical distribution and identify the positions contributing to the top five percent of the corresponding cumulative density function (CDF, Figs 4E, 4F and S2). The t-distribution appears to generally fit the ICs well in all cases studied to date (S5 Fig), and IC composition is robust to alignment size when diversity is maintained (S6 Fig). The CDF cutoff is an adjustable parameter, but 5% seems to agree well with experimental significance in the model systems studied [16, 21]. We then construct a sub-matrix of ${\tilde{C}}_{ij}$ that contains only the selected top-scoring positions for the k* ICs, ordered by their degree of contribution to each IC. For the G protein family, this corresponds to a matrix of 54 positions that contribute to the top four significant ICs (Fig 9A). This sub-matrix describes both the pattern of “internal” correlations between positions that make up each IC (the diagonal blocks), and the pattern of “external” correlations between ICs (the off-diagonal blocks). This representation shows that ICs 1, 2, and 3 display a set of transitive inter-IC correlations, with IC1 correlated to IC2 and IC2 correlated to IC3, indicating that IC1–3 together comprise the hierarchically decomposed parts of a single sector (sector 1, Fig 9A and 9B). In contrast, IC4 shows near-independence from the other ICs, suggesting that it defines a distinct sector (sector 2, Fig 9A and 9B).

These sector definitions are made exclusively from analysis of the IC-based submatrix of ${\tilde{C}}_{ij}$, but correspond to a meaningful spatial architecture in the G protein. These proteins are binary switches that display different conformations depending on the identity of their bound guanine nucleotide [25, 26]. The exchange of GTP for GDP triggers two specific conformational changes: clamping of the so-called switch I loop closer to the nucleotide binding pocket, and transit of a disordered and weakly interacting surface loop (switch II) to an ordered helix that is well-packed against the core domain (Fig 9C) [25]. Sector 1 comprises a physically contiguous group of amino acid residues that shows excellent agreement with the nucleotide-dependent allosteric mechanism [50]. The sector is compact in the GTP-bound state but partially disrupted in the GDP-state (Fig 9B and 9C), a finding consistent with the state-dependent connectivity between the nucleotide-binding pocket and the switch loops. Furthermore, the hierarchical breakdown of sector 1 into its constituent ICs 1, 2 and 3 reveals a meaningful structural organization: IC3 (cyan) defines a physically contiguous network that comprises the nucleotide binding pocket, IC1 (light blue) defines the packing interactions between switch II and the core domain, and IC2 (dark blue) represents a set of surface accessible positions (including switch I) that link to the buried core of sector 1 (Fig 9C). Nucleotide exchange substantially reorganizes the structure and connectivity of IC1 and 2, but is largely inconsequential for IC3 (Fig 9C).

Consistent with assignment as an independent sector, sector 2 (IC4, red) also comprises a mostly physically contiguous group within the core of the G protein (Fig 9B); like IC3 of sector 1 (cyan) it shows no nucleotide-dependent conformational plasticity. These results are interesting since IC1 and IC2 (but not IC3 or IC4) are associated with the divergence of functional sub-classes of G protein (Fig 7A). The data suggest that IC3 (cyan subset, sector 1) and IC4 (sector 2) are global functional modes shared by all members of the G protein family, while ICs 1 and 2 correspond to subsets of sector 1 that are specialized for tuning allosteric or effector-binding properties within sub-classes of G proteins. These observations represent new hypotheses for further study.

For the S1A family, the IC-based submatrix shows little evidence of inter-IC correlations, and thus we conservatively treat all ICs as separate sectors (S7 Fig). Each sector corresponds to a largely contiguous network of amino acids in the protease tertiary structure, a decomposition consistent with the orthogonal sequence divergences and with previous reports (S7 Fig, and [10]). Examples of sector analysis for two other protein families—the dihydrofolate reductases [51] and the class A beta-lactamases [52]—are provided in S8 Fig and in tutorials (S3 Text).

The process of sector identification presented here is heuristic, requiring the judgement of the practicing scientist to determine the grouping of ICs to form sectors. This reflects that fact that various degrees of independence between ICs are possible depending on the statistical nature of selective pressures operating on a protein family. Thus, an automated approach to interpreting the ${\tilde{C}}_{ij}$ matrix awaits more broad experience with sector analysis in many protein families. Given the importance of interpreting hierarchical correlation matrices in general (e.g [53–55]), it seems reasonable that such automation might be achieved with further work.

## Discussion

A fundamental goal in biology is to understand the architectural principles of proteins—the pattern of constraints on and between amino acids that underlies folding, biochemical activities, and adaptation. An emerging approach is to leverage the growing databases of protein sequences to statistically infer these constraints from large and diverse ensembles of homologous sequences. This strategy has two defining features that distinguish it from the more traditional direct physical study of specific model proteins. First, by averaging over the space of homologs, the statistical approach emphasizes the general constraints shared by many related proteins over those that are idiosyncratic to particular proteins. Second, by quantitatively examining the structure of correlations, the statistical approach provides models for the global pattern of cooperativity between amino acids. SCA adds an extra concept; by weighting correlations with a function of the evolutionary conservation of the underlying amino acids, this approach incorporates a measure of their functional relevance [56, 57]. Mathematical decomposition of the weighted coevolution matrix reveals an internal architecture for proteins in which the basic functional units are groups of amino acids called sectors. The sector architecture is consistent with two empirically known but poorly understood properties of proteins: (i) *sparsity*, such that only a fraction of the amino acids are functionally critical [21, 58], and (ii) *distributed cooperativity*, such that folding and function can depend on the coupled action of amino acids linking distantly positioned sites [59–61]. It has also revealed a previously unrecognized feature of proteins: *modularity*, such that multiple functionally distinct sectors are possible in a single protein domain [10].

### A hierarchical model for sectors

Previous work has introduced the concept of sectors as quasi-independent units of protein structure that are associated with distinct functional properties [10], but has largely ignored their internal architecture. This work presents a more refined description in which a sector may itself be decomposed into a physically contiguous core element (e.g. IC3, Fig 9C), surrounded by peripheral elements (e.g. ICs 1 and 2, Fig 9C) that have the property of differential variation along functional branches of a protein family (Fig 7A). Thus, we propose a model that sectors are structural units of function and the ICs define patterns of variation within these units.

These observations also highlight the practical value of the mapping between positional correlations and sequence subfamilies. When functional divergences between subfamilies are annotated, the mapping can identify the positions responsible for this divergence. For example, in the Hsp70 family of chaperones, the existence of subfamilies with known differences in allosteric function led to the identification of positions involved in the underlying mechanism [17]. Turned around, when the role of specific positions in a protein is known, the mapping can help annotate sequences according to the associated functional property. For example, sequence divergence within sector positions with known function in the S1A family permitted classification of the sequence space according to that functional property [10]. In principle, high-throughput methods for functional annotation of members of a protein family should permit even more refined mappings between amino-acid variation and phylogenetic or functional divergence, a step towards relating genotype-to-phenotype at the molecular level.

### Relationship to other methods

It is valuable to explain the similarities and distinctions of SCA with other analyses of coevolution in multiple sequence alignments. The direct coupling analysis (DCA [4]) and its various extensions [9, 62–64] are focused on using coevolution to determine physical contacts between amino acids within or between protein tertiary structures. As different as this problem may seem from discovering the pattern of *functionally* coevolving amino acids, there is a deep relationship. Recent work shows that the two approaches focus on two extremes of the same hierarchical architecture of coevolution [7, 8]. SCA focuses on the global modes of coevolution (the top eigenmodes of a conservation-weighted correlation matrix), and DCA on the minimal units of coevolution (the bottom eigenmodes of an unweighted correlation matrix). Thus, coevolving direct contacts are at one end of the hierarchy and sectors at the other. Consistent with this, coevolving direct contacts are found within sectors and outside of sectors, but not bridging two independent sectors [8]. Another approach for analyzing coevolution in protein alignments is mutual information, which has been successful at predicting the amino acids responsible for specificity in some protein-protein interactions [5]. The distinction between this method and SCA lies in the nature of the weighting function *ϕ*; in essence, the mutual information method uses flat positional weights (*ϕ* = 1), which has the effect of emphasizing more unconserved correlations and may therefore be more appropriate when studying rapidly diverging functional properties [40]. Taken together, these observations begin to clarify the relationship of the different approaches, and poses the question of the nature of physical information held at various levels of the hierarchy of coevolution, a matter for future experimentation. From a theoretical point of view, the observations highlight the need for a better, more unified framework representing the full hierarchy in amino acid correlations in proteins, a key next goal in advancing the statistical approach to the biology of proteins.

### Conclusion

Sector analysis provides a representation of proteins that is distinct from the first-order analysis of positional conservation and that (so far) is not obtained from structure determination or functional mutagenesis. Thus, it provides a valuable tool for directing experimental studies of protein folding and function, and ultimately, for formulating a physical and evolutionary theory consistent with the design of natural proteins.

## Materials and Methods

Multiple sequence alignments were obtained from previous work [10] or from the PFAM database (release 27.0, accession codes PF00071 (G proteins), PF00186 (DHFR), and PF13354 (class A *β*-lactamases)), and were subject to pre-processing with default parameter values as described in Box 1. Reference sequences/structures selected for each family were rat trypsin (PDB 3TGI), human Ras (PDBs 5P21 and 4Q21), E. coli DHFR (PDB 1RX2), and E. coli TEM-1 *β*-lactamase (PDB 1FQG), and with sub-sampling to the number of effective sequences, yielded the following final alignment statistics: S1A serine proteases (928 effective sequences by 205 positions), G proteins (3366 effective sequences by 158 positions), DHFR (1157 effective sequences by 151 positions), *β*-lactamase (497 effective sequences by 200 positions). All calculations were carried out using a new python implementation of the statistical coupling analysis (pySCA v6.2), following the algorithms described in Box 1 and in the main text. Step-by-step tutorials for executing the analysis for the four protein families are provided in the S3 Text and accompany the toolbox distribution. The pySCA toolbox is available for download through GitHub (https://github.com/reynoldsk/pySCA), and with online instructions at http://reynoldsk.github.io/pySCA.

## Supporting Information

S1 Text Statistical Coupling Analysis: supplementary methods and codes. We provide a more detailed description of the SCA method. The pySCA toolbox is available for download through GitHub (https://github.com/reynoldsk/pySCA), and with online instructions at http://reynoldsk.github.io/pySCA.
(PDF)

S2 Text Description and usage of the pySCA toolbox. The pySCA toolbox (v.6.1) is a distribution of SCA written in Python and comprises a library of functions (scaTools.py), four scripts to automate most calculations (scaAnnotateMSA.py, scaProcessMSA.py, scaCore.py, and scaSectorID.py), and several tutorials written using the interactive python notebook environment (iPython notebook). Here we describe installation of this toolbox, its usage via the scripts, and provide a list of classes and functions in the scaTools.py module with hyperlinks to access the main code documentation online.
(PDF)

S3 Text Tutorials. We provide tutorials to describe the sector identification process for four protein families, with the goal of illustrating several features of the SCA. The tutorials are additionally available online as html files, and can be downloaded as interactive python notebooks for use with the pySCA toolbox (https://github.com/reynoldsk/pySCA).
(PDF)

S1 Table Updates in pySCA 6.1. (JPG)

S1 FigDimension reduction of ${\tilde{C}}_{ij}^{ab}$.**A**, The amino acid correlation matrix for positions 47 and 59 (${\tilde{C}}_{47,59}^{ab}$) in the dihydrofolate reductase (DHFR) alignment and the corresponding singular value decomposition. The decomposition shows the obvious dominance of the first singular value (the “spectral norm”). **B**, Two spatially proximal positions in DHFR chosen for illustrating properties of the SCA correlation tensor ${\tilde{C}}_{ij}^{ab}$. **C**, The ${\tilde{C}}_{47,59}^{ab}$ matrix reconstructed from just the top singular value (${\tilde{C}}_{47,59}^{ab}=P_{47,59}^{a1}\lambda_{47,59}^{1}Q_{47,59}^{1b})$, and **D**, a scatterplot comparing the original and reconstructed matrices. The data demonstrate the sufficiency of the spectral norm in this case. **E**, the spectral norm for all pairs of positions *i*, *j* plotted against the Frobenius norm defined by ${\left(\sum_{c}{\left(\lambda_{ij}^{c}\right)}^{2}\right)}^{1/2}$, a measure of the magnitude of ${\tilde{C}}_{ij}^{ab}$ where all the singular values are retained. The data demonstrate the general sufficiency of the spectral norm.(JPG)

S2 FigRobustness of k*—the number of significant eigenmodes of ${\tilde{C}}_{ij}$—to randomization trials and sampling of sequences.**A**, The histogram of eigenvalues (the “eigenspectrum”) of ${\tilde{C}}_{ij}$ for the G protein family (black bars) and for the average of *N* = 10 trials of random shuffling of amino acids at each position in the alignment, independently (red line) (reproduced from Fig 4A, main text). The randomization process exactly preserves the frequencies of amino acids at each position (the “first-order” statistics), but eliminates all correlations except those due to finite sampling. Since the first eigenvalue is strongly dependent on the first-order statistics, it is ignored in determining k*. The cutoff for significant eigenvalues is $\lambda_{2}^{rand}+2\sigma$, the second random eigenvalue plus two standard deviations computed over *N* randomization trials. Panels **B-C** show the robustness of the cutoff for different values of *N*, and **E-F** shows robustness over different independent trials of sub-sampling the alignment sequences to preserve the same number of effective sequences (here, *M*_*eff*_ = 3366 out of a total of 16294 after alignment pre-processing steps). See main text and Box 1 for alignment pre-processing and calculation of number of effective sequences. The analysis shows that k* is highly robust to both number of randomization trials and to the sampling of sequences in the MSA.(PDF)

S3 FigThe pattern of amino acid contributions to positional coevolution.**A-C**, As described in S1 Text, section H, S1 Fig, one property of the SCA correlation tensor ${\tilde{C}}_{ij}^{ab}$ is compressibility, such that the information in each 20 × 20 amino acid coevolution matrix for each pair of positions (*i*, *j*) can be represented by a scalar value $\lambda_{ij}^{1}$, the top singular value (**B**). Per the SVD (**A**), the top singular value is associated with the top left and right singular vectors $P_{ij}^{a1}$ and *Q*1*b*_*ij*_, which contain the weights for the contributions of amino acids at positions i and j, respectively. Since coevolution is a symmetric property of amino acids at two positions (${\tilde{C}}_{ij}^{ab}={\tilde{C}}_{ji}^{ba}$, we can further simplify the SVD further as in **C**. **D-E**, Besides compressibility, another empirical property of ${\tilde{C}}_{ij}^{ab}$ is that for any given position *i*, the top singular vector $P_{i,j}^{a1}$ is essentially invariant over all j; that is the amino acids by which position i coevolves with other positions j is nearly the same. For example, for three positions within the core of the G protein (**D**, positions 82, 125, and 130), the amino acids by which other positions i coevolve with these positions varies, but the amino acids by which these positions coevolve with other positions j is nearly the same (**E**. Thus, it is possible to define a projection for each position (S1 Text. Eq 19) by which the alignment tensor $x_{si}^{a}$ can be reduced to an alignment matrix *x*_*si*_ (S1 Text, Eq 20).(PDF)

S4 Fig Sequence-position mapping for the DHFR protein family. **A-F** show the positions comprising the six ICs of the SCA coevolution matrix, respectively, as colored spheres on the structure of E. coli DHFR (PDB 1RX2), and **G-L** show stacked histograms of the corresponding sequence space colored colored by phylogenetic annotation. The data show that ICs 1, 5, and 6 roughly separate eukaryotic and prokaryotic sequences and that the remainder are more homogeneous with regard to phylogenetic divergence. The significance of these apparent heterogeneities will require further investigation, but prior work demonstrates functional and mechanistic differences between the eukaryotic and prokaryotic members of this protein family [65].
(PDF)

S5 FigIndependent components of the G protein family.The independent components (ICs) corresponding to the four top eigenmodes of the ${\tilde{C}}_{ij}$ matrix. The solid red line is a fit to the t-distribution, with the cutoff indicated representing the top 95% of the cumulative density function. The ICs are generally well-fit by this empirical distribution, and serves as a basis for systematic definition of coevolving positions.(PDF)

S6 FigThe robustness of the independent components (ICs) of the ${\tilde{C}}_{ij}$ matrix to alignment sub-sampling.Scatterplots of the top four independent components (ICs) for the full alignment of G proteins (*M*_*eff*_ = 3366) against those for four trials of sub-sampling the alignment to ≈15% of sequences. The analysis shows that the composition of ICs (and therefore sector definitions and sequence projections) are highly robust to the number of effective sequences in the alignment.(PDF)

S7 FigSectors in the S1A protein family.**A** shows the IC-based sub-matrix of the ${\tilde{C}}_{ij}$ matrix for the S1A family and **B-G** shows the positions corresponding to each IC on a representative structure of a member of the protein family (rat trypsin, PDB 3TGI). Each IC shows a hierarchical pattern of correlation between constituent positions, with little compelling evidence for strong inter-IC correlations. Consistent with this, each IC corresponds to a distinct and largely contiguous network of amino acid contacts in the protein structure (**B**). ICs 1–3 correspond to sectors defined in Halabi et al. [10].(PDF)

S8 FigSector identification for the DHFR and *β*-lactamase protein families.Panels **A** and **C** show the IC-based sub-matrix of the $\tilde{C_{ij}}$ matrix for the DHFR and the *β*-lactamase protein families. The cartoons at right indicate the sector analyses. DHFR displays considerable transitive external correlations between ICs, suggesting a single sector. The *β*-lactamase family displays two sectors, one comprising IC2 and the other comprising ICs1, and 3–6. Panels B and D show the positions contributing to each IC mapped to the protein structure; in each case the sectors form physically contiguous structural units.(PDF)
