# Supplementary material for: Evolution-Based Functional Decomposition of Proteins
Source: PLoS Comput Biol. 2016 Jun 2;12(6):e1004817. doi: 10.1371/journal.pcbi.1004817 (PMC4890866; doi:10.1371/journal.pcbi.1004817)
Supplement: S1 Text — We provide a more detailed description of the SCA method. The pySCA toolbox is available for download through GitHub (https://github.com/reynoldsk/pySCA), and with online instructions at http://reynoldsk.github.io/pySCA. (PDF) [file pcbi.1004817.s001.pdf]

S1 Text. Statistical coupling analysis: supplementary methods and codes

Here, we provide a more complete description of the SCA approach. The pySCA toolbox is available for download through GitHub (`https://github.com/reynoldsk/pySCA`), and with online instructions at `http://reynoldsk.github.io/pySCA`.

A. The multiple sequence alignment

As shown in Figure 1, a multiple sequence alignment of M sequences by L positions can be represented as a three-dimensional binary array x_{si}^a, where $x_{si}^a = 1$ if sequence s has amino acid a at position i, and 0 otherwise; gaps are ignored and always set to 0. With sequence weights, the amino acid frequencies at individual positions are $f_i^a = \langle x_{si}^a \rangle_s \equiv \sum_s w_s x_{si}^a / M'$, where $M' = \sum_s w_s$ represents the effective number of sequences in the alignment. Similarly, joint frequencies of amino acids between pairs of positions are defined by $f_{ij}^{ab} = \langle x_{si}^a x_{sj}^b \rangle_s \equiv \sum_s w_s x_{si}^a x_{sj}^b / M'$.

B. Positional Conservation

The conservation of each position in the alignment is measured by the divergence of the observed frequency f_i^a of amino acid a at position i from the background probability q^a of amino acid a. This background probability is computed from the mean frequency of amino acid a in all proteins in the NCBI non-redundant database (1):

A	C	D	E	F	G	H	I	K	L	M	N	P	Q	R	S	T	V	W	Y
0.073	0.025	0.050	0.061	0.042	0.072	0.023	0.053	0.064	0.089	0.023	0.043	0.052	0.040	0.052	0.073	0.056	0.063	0.013	0.033

where the letters refer to the standard one-letter abbreviation for amino acids. Given this, the probability $P_M[f_i^a]$ of observing the actual frequency f_i^a in an alignment of M ideally sampled sequences is given by the binomial density function:

$$P_M[f_i^a] = \frac{M!}{(Mf_i^a)!(M(1-f_i^a))!}(q^a)^{Mf_i^a}(1-q^a)^{M(1-f_i^a)}. \quad (1)$$

When M is large (the relevant limit for the analysis), the Stirling formula leads to the approximation

$$P_M[f_i^a] \simeq e^{-MD_i^a}, \quad \text{with} \quad (2)$$

$$D_i^a = f_i^a \ln \frac{f_i^a}{q^a} + (1-f_i^a) \ln \frac{1-f_i^a}{1-q^a}. \quad (3)$$

D_i^a is the Kullback-Leibler relative entropy (2), indicating how unlikely the observed frequency of amino acid a at position i would be if a occurred randomly with probability q^a - a quantitative measure of position-specific conservation.

C. Definition of overall positional conservation

As described in the main text, Eq.3 gives the conservation of each amino acid a at each position i. An overall positional conservation D_i taking into account the frequencies of all 20 amino acids can also be defined, but requires introducing a background probability for gaps; for instance, $\bar{q}^0 = \sum_i f_i^0 / L$, the fraction of gaps in the alignment, with $f_i^0 = 1 - \sum_{a=1}^{20} f_i^a$ the fraction of gaps at position i, and $\bar{q}^a = (1-\bar{q}^0)q^a$. The probability of jointly observing the frequencies $(f_i^1, \ldots, f_i^{20})$ of each of the 20 possible amino acids at position i is given by

$$P_M[f_i^1, \cdots, f_i^{20}] = \frac{M!}{(Mf_i^0)! \cdots (Mf_i^{20})!}(\bar{q}^0)^{Mf_i^0} \cdots (\bar{q}^{20})^{Mf_i^{20}} \simeq e^{-MD_i}, \quad (4)$$

where $D_i = \sum_{a=0}^{20} f_i^a \ln(f_i^a / \bar{q}^a)$ defines the overall conservation at position i.

D. Positional weights from bootstrap resampling

SCA involves construction of a conservation weighted correlation matrix $\tilde{C}_{ij}^{ab} = \phi_i^a \phi_j^b (f_{ij}^{ab} - f_i^a f_j^b)$ (Eq.4, main text), with the weights ϕ controlling the degree of emphasis on conservation. An approach to define the weights ϕ_i^a is through a bootstrap resampling procedure on the alignment. The idea is to consider the effect on the conservation of each position i upon removing each sequence s. This "perturbation" is taken as an estimate of the significance of each amino acid at each position in the alignment by its impact on the measure of conservation used - here, the relative entropy D_i^a, defined in Eq.3. To develop this formally, let M_i^a be the number of sequences with amino acid a at position i, and M be the total number of sequences. When sequence s is left out, the frequency $f_i^a = M_i^a/M$ becomes

$$f_{i,s}^a = \frac{M_i^a - x_{si}^a}{M-1} = \left(1 + \frac{1}{M}\right) f_i^a - \frac{x_{si}^a}{M} + O\left(\frac{1}{M^2}\right), \tag{5}$$

where we remind that $x_{si}^a = 1$ if sequence s has amino acid a at position i, and 0 otherwise. In the limit of large number of sequences M, expanding D_i^a, viewed as a function of $f_{i,s}^a$, to first order in $1/M$ leads to

$$D_{i,s}^a \approx \hat{D}_i^a - \frac{x_{si}^a}{M} \frac{\partial D_i^a}{\partial f_i^a}, \tag{6}$$

where $\hat{D}_i^a$ is the relative entropy D_i^a with f_i^a replaced by $(1 + 1/M)\, f_i^a$. Ignoring the scaling factor of $1/M$ (or, equivalently, rescaling the perturbation in conservation $D_{i,s}^a - \hat{D}_i^a$ by M to be independent of alignment size), we find that this perturbation approach indicates a weighting function ϕ_i^a for the alignment that is the gradient of relative entropy:

$$\frac{\partial D_i^a}{\partial f_i^a} = \ln\left[\frac{f_i^a(1-q^a)}{(1-f_i^a)q^a}\right]. \tag{7}$$

The definition of the SCA amino acid correlation matrix (Eq.4, main text) with weights defined here is used in all versions of SCA from 2.0 to current.

E. Older versions of SCA

The original implementation of SCA (v. 1.0-1.5) defined conservation with different notations and defined co-evolution through a more specific type of perturbation analysis on the sequence alignment. Here, we describe the equivalence of the original conservation definition with this work and the conceptual similarity of the definition of coevolution.

1. Equivalence with previous definitions of conservation

D_i^a is equivalent to measures of positional conservation introduced in previous reports of the SCA method. In essence, D_i^a is the asymptotic limit for large M for $\Delta G_i^{\text{stat},a}$ (SCA MATLAB Toolbox v1.0, as reported in Refs. (3–6)), and $\Delta E_i^{\text{stat},a}$ (SCA Toolbox v1.5, as reported in Ref. (7)):

$$\Delta G_i^{\text{stat},a} = \Delta E_i^{\text{stat},a} = -\frac{1}{M} \ln P_M[f_i^a] \simeq D_i^a. \tag{8}$$

The pre-factor $-1/M$ scales the positional conservation parameter for alignments of different size, and represents the statistical unit of conservation symbolically indicated by kT^* or γ^* in previous works.

2. The original SCA method

The implementation of the SCA method introduced originally in Ref. (5) was based on a perturbation to the amino acid distribution at one test site i to measure the difference in position-specific conservation of each amino acid at a second site j. In general, the perturbation consisted of restricting the test site to the most prevalent amino acid a_i, a manipulation that extracts a sub-alignment with size equal to $f_i^{a_i} M$. For test sites in which sub-alignments

retained sufficient size and diversity to be globally representative of the full alignment (i.e., $f_i^{a_i} M > 100$ sequences), a difference conservation value was calculated:

$$\Delta\Delta G_{j,i}^{\mathrm{stat},b,a_i} = \Delta\Delta E_{j,i}^{\mathrm{stat},b,a_i} = -\frac{1}{M}\left[\ln\left(P_M\left[f_j^b\right]\right) - \ln\left(P_M\left[f_{j|i}^{b|a_i}\right]\right)\right], \tag{9}$$

where $f_{j|i}^{b|a_i}$ is the frequency of amino acid b in the sub-alignment obtained by retaining only the sequences having a well represented amino acid a_i at position i. $\Delta\Delta G_{j,i}^{\mathrm{stat},b,a_i}$ represents the change in the conservation of amino acid b at position j due to the perturbation introduced at position i, a measure of their correlation (the term was renamed to $\Delta\Delta E$ in subsequent publications and is ignored entirely now to avoid confusion with physical energies). The first term on the right hand side, $-\ln\left(P_M\left[f_j^b\right]\right)/M$, corresponds to D_j^b. A basic tenet of the original SCA approach was that perturbations lead to sub-alignments that are representative of the full alignment, a condition satisfied typically by only the most frequent amino acid at a subset of positions. Under this assumption, $f_{j|i}^{b|a_i} \approx f_j^b$ for most amino acids b at positions j. We may therefore expand the second term, $-\ln P_M\left[f_{j|i}^{b|a_i}\right]/M$, by writing

$$f_{j|i}^{b|a_i} = \frac{f_{ij}^{a_i b}}{f_i^{a_i}} = f_j^{(b)} + \frac{f_{ij}^{a_i b} - f_i^{a_i} f_j^b}{f_i^{a_i}} = f_j^b + \frac{C_{ij}^{a_i b}}{f_i^{a_i}} \tag{10}$$

with $C_{ij}^{a_i b}$ defined as in Eq.4, so that

$$-\frac{1}{M}\ln\left(P_M\left[f_{j|i}^{b|a_i}\right]\right) \approx D_j^b + \frac{C_{ij}^{a_i b}}{f_i^{a_i}}\frac{\partial D_j^b}{\partial f_j^b}. \tag{11}$$

This leads to

$$\Delta\Delta G_{j,i}^{\mathrm{stat},b,a_i} \approx -\frac{1}{f_i^{a_i}}\frac{\partial D_j^b}{\partial f_j^b} C_{ij}^{a_i b}, \tag{12}$$

which shows that the perturbation procedure also represents a weighted procedure for correlations that is fully consistent with the general principles presented in the main text.

F. Reduction to positional correlations

$\tilde{C}_{ij}^{ab}$ is a four-dimensional array of L positions $\times$ L positions $\times$ 20 amino acids $\times$ 20 amino acids, but its analysis shows that it may be compressed into a $L \times L$ matrix of positional correlations. To explain, we use an elementary method from linear algebra for factorizing matrices known as the singular value decomposition (SVD). The SVD for $\tilde{C}_{ij}^{ab}$ for a given pair of positions (i, j) is:

$$\tilde{C}_{ij}^{ab} = \sum_{k=1}^{20} P_{ij}^{ak} \lambda_{ij}^{k} Q_{ij}^{kb}. \tag{13}$$

Per this decomposition, each 20×20 amino acid coevolution matrix for each (i, j) is written as a product of three 20×20 matrices: λ, a diagonal matrix of singular values (ranked by magnitude), and P and Q, orthogonal matrices whose columns contain the associated left and right singular vectors (Fig. xx). Each singular value indicates the quality of variance in $\tilde{C}_{ij}^{ab}$ captured, and each corresponding left and right singular vector gives the weights for the combination of amino acids at positions i and j that contribute to this variance. One obvious approach to dimension reduction of $\tilde{C}_{ij}^{ab}$ is to compute the Frobenius norm of each 20×20 amino acid coevolution matrix for each pair of positions (i, j):

$$\tilde{C}_{ij} = \sqrt{\sum_{k=1}^{20} \left(\lambda_{ij}^{k}\right)^2}. \tag{14}$$

This defines the SCA positional coevolution matrix $\tilde{C}_{ij}$ (Eq.5 of the main text). However, examination of the singular values for each (i, j) suggests the sufficiency of an even simpler matrix norm. Specifically, the SVD shows that for

essentially every (i,j) the first singular value dominates the others, $\lambda_{ij}^1 \gg \lambda_{ij}^k$ for $k \neq 1$ (Fig. xx). That is, the information in the amino acid correlation matrix for each pair of positions can be effectively represented by just the top singular value (a quantity also known as the spectral norm):

$$\tilde{C}_{ij}^{ab} \simeq P_{ij}^{a1} \lambda_{ij}^1 Q_{ij}^{1b}. \tag{15}$$

As an example, we show the SVD of the amino acid correlation matrix for two amino acid positions (47 and 59) that make direct contact in DHFR (Fig. S1B). Consistent with Eq. (15), the amino acid correlation matrix reconstructed from just the top singular mode shows near perfect agreement with the original matrix (Fig. 1C-D). Thus, a matrix of positional correlations $\tilde{C}_{ij}$ can also be defined simply by taking the spectral norm of $\tilde{C}_{ij}^{ab}$ for each pair (i,j) of positions:

$$\tilde{C}_{ij} = \lambda_{ij}^1. \tag{16}$$

The sufficiency of the spectral norm is illustrated by the nearly-perfect agreement of the $\tilde{C}_{ij}$ matrix computed using the spectral norm with that computed by using the Frobenius norm, which retains all the singular values for each (i,j) (Fig.1E). In the current implementation of the SCA codes (v6.0), we continue to use the Frobenius norm by default but permit optional return of the spectral norm for further examination of the generality of this result.

G. Spectral decomposition and Independent component analysis (ICA)

As described in the main text, the first step in analyzing the SCA positional coevolution matrix $\tilde{C}_{ij}$ is eigenvalue decomposition, a process that diagonalizes (uncorrelates) the coevolution matrix by linearly combining the amino acid positions into eigenmodes; the elements of the diagonalized matrix are the eigenvalues which indicate the magnitude of information in $\tilde{C}_{ij}$ captured, and the corresponding eigenvectors give the weights for combining amino acid positions. To determine the number of significant eigenmodes, we compare the histogram of eigenvalues of the $\tilde{C}_{ij}$ matrix (the eigenvale spectrum) with the average spectrum for many trials (default 10) of randomizing the alignment. In this randomization procedure, columns of the alignment are scrambled independently; this preserves the first order constraints on sequence positions while removing all correlations not due just to finite sampling noise. Note that the randomized eigenspectrum still retains a large first eigenvalue (due to preservation of the first order constraints on positions). Accordingly, we define the number of significant eigenmodes (k^*) to be those above the average *second* eigenvalue plus 2 standard deviations. This cutoff is robust to the number of randomization trials and to the precise composition of the alignment as long as the number of effective sequences is preserved (Fig. Sxx).

By this definition, the top k^* eigenvectors $\tilde{V}_{ik}$ of $\tilde{C}_{ij}$ are uncorrelated but so are any combination of these vectors obtained by rotating them. Using the eigenvectors to represent patterns of coevolution implicitly includes an additional constraint - the maximization of variance captured, which is indeed desirable for the purpose of reducing the analysis of coevolution patterns to just the space spanned top few eigenmodes. However, several other criteria can be used to specify a rotation of the top k^* eigenvectors, such as sparsity or independence. Independent component analysis (ICA (8)) uses this later criterion to define maximally independent components through a numerical optimization scheme.

Different implementations of ICA use different measures of independence and different algorithms for optimizing them. Here, we use one of the simplest implementations of ICA, called infomax (9), with modifications introduced in Ref. (10). We take as input the top k^* eigenvectors of a correlation matrix, which we concatenate in a $k^* \times L$ matrix V. The algorithm iteratively updates an unmixing matrix W, starting from the $k^* \times k^*$ identity matrix $W_0 = I_{k^*}$, with increments ΔW given by

$$\Delta W = \rho \left(I_{k_{\text{top}}} + \left(1 - \frac{2}{1 + \exp(-WV)} \right) (WV)^\top \right) W. \tag{17}$$

The parameter ρ is a learning rate that has to be sufficiently small for the iterations to converge.

The independent components $\tilde{V}_{ik}^p$ are obtained by applying W to the eigenvectors in $\tilde{V}$. To set their overall scale and sign, we normalize them to unit length ($\sum_i (V_{ik}^p)^2 = 1$) and orient them so that the position i with largest $|V_{ik}^p|$ satisfies $V_{ik}^p > 0$. The order of the independent components, which is not necessarily prescribed in other versions of ICA, is here well defined by the algorithm and is related to the order of the principal components.

H. Mapping between sequence and position correlations

In the main text, we present the mapping between sequence and amino acid correlations in the context of an unweighted full alignment X_{sn}. Here we describe the necessary steps to apply this approach to the conservation-weighted dimension-reduced convolution matrix $\tilde{C}_{ij}^{ab}$. To include sequence weights in the analysis, F (the matrix of sequence similarities) is redefined as $F'_{nm} = \sum_s w_s X_{sn} X_{sm}/M'$, where $M' = \sum_s w_s$ is the effective number of sequences in the alignment. Then, given Λ' and V', the eigenvalues and eigenvectors of $M'F'$, a mapping to the sequence space is obtained by

$$U' = XV'\Lambda'^{1/2}. \quad (18)$$

Mapping to sequence space the components of the dimension-reduced $L \times L$ coevolution matrix $\tilde{C}_{ij}$, defined by Eq. (16), is not immediate, given that it is not a proper covariance matrix of the form $\tilde{C} = \tilde{X}^\top \tilde{X}/M'$. However, an empirical property of the correlations $\tilde{C}_{ij}^{ab}$ provides a simple solution. We showed earlier that the SVD in Eq. (13) has the property that the information in the 20×20 amino acid coevolution matrix for each i, j is compressible to a single scalar value, the top singular value, or spectral norm, see Eq. (16). It also turns out that for any given position i, the top singular vector P_{ij}^{a1} corresponding to the top singular value is (up to the sign) essentially invariant over all positions j (Fig. S4). That is, the amino acids by which a position i makes significant correlations with other positions j is nearly the same, and therefore can be sufficiently described by just the amino acid distribution at position i taken independently. Indeed, we can define a $L \times 20$ matrix

$$\bar{P}_i^a = \frac{\phi_i^a f_i^a}{\left(\sum_b (\phi_i^b f_i^b)^2\right)^{1/2}}, \quad (19)$$

whose rows specify the combination of amino acids at each position that contribute to the observed correlations in $\tilde{C}_{ij}$. Thus, using $\bar{P}_i^a$, we can reduce the dimensionality of the alignment x_{si}^a from a $M \times L \times 20$ array to an $M \times L$ matrix x_{si}:

$$x_{si} = \sum_a \bar{P}_i^a x_{si}^a. \quad (20)$$

In x_{si}, each position i of each sequence s is no longer a 20-dimensional vector, but just a single value representing the weight of the amino acid at (s, i) as given by the projection matrix $\bar{P}_i^a$. The dimension-reduced alignment x_{si} now provides the mapping between the space of positional coevolution (in the top ICs of the $\tilde{C}_{ij}$ matrix) and the corresponding sequence space. Specifically, if $\tilde{\Delta}$ and $\tilde{V}$ are the eigenvalues and eigenvectors, respectively, of the positional coevolution matrix $\tilde{C}_{ij}$, then

$$\tilde{U} = x\tilde{V}\tilde{\Delta}^{-\frac{1}{2}} \quad (21)$$

represents the structure of the sequence space (now with both position and sequence weights) corresponding to the patterns of positional coevolution in $\tilde{V}$. Furthermore, if W is the matrix derived from ICA of $\tilde{V}_{1\ldots k^*}$, Eq.7 of main text, then

$$\tilde{U}^p = W\tilde{U} \quad (22)$$

represents the sequence space corresponding to $\tilde{V}^p$, the ICs of the $\tilde{C}_{ij}$ matrix.

I. Weights ϕ as redefining the similarity between sequences

The mapping between sequence and position correlations provides an interpretation in terms of sequence similarity for the weights ϕ in Eq. (4). Ignoring for simplicity the sequence weights w_s, changing the pairwise frequencies $F_{nm} = \sum_s X_{sn} X_{sm}/M$ to weighted pairwise frequencies $\tilde{F}_{nm} = \phi_n \phi_m F_{nm}$ corresponds to changing X_{sn} to $\tilde{X}_{sn} = \phi_n X_{sn}$, and therefore the sequence similarity $S_{rs} = \sum_n X_{sn} X_{sm}/L$ to $\tilde{S}_{rs} = \sum_n (\phi_n)^2 X_{rn} X_{sn}/L$. This redefinition of the similarity between sequences gives more importance to the positions that are more conserved. As a consequence, two sequences differing at conserved sites are considered to be more dissimilar than two sequences differing at the same number of less conserved sites. To the extent that conservation reflects functional significance, this defines a metrics between sequences that better reflects functional relationships.

Reciprocally, if one accepts that the conservation-weighted similarity $\tilde{S}_{rs}$ better reflects functional relationships than S_{rs}, then the mapping between sequence and position correlations indicates that $\tilde{F}_{nm}$ (or $\tilde{C}_{ij}^{ab}$) should be more adequate for describing functional correlations between positions than F_{nm} (or, respectively, C_{ij}^{ab}). This justifies the weights ϕ in Eq. (4), main text or (Eq. (7) here) from the viewpoint of sequence similarities.

References

[1] Pruitt KD, Tatusova T, Maglott DR. NCBI reference sequences (RefSeq): a curated non-redundant sequence database of genomes, transcripts and proteins. Nucleic Acids Res. 2007 Jan;35(Database issue):D61–5.
[2] Cover TM, Thomas JA. Elements of information theory. John Wiley & Sons; 2012.
[3] Hatley ME, Lockless SW, Gibson SK, Gilman AG, Ranganathan R. Allosteric determinants in guanine nucleotide-binding proteins. Proc Natl Acad Sci U S A. 2003 Nov;100(24):14445–50.
[4] Süel GM, Lockless SW, Wall MA, Ranganathan R. Evolutionarily conserved networks of residues mediate allosteric communication in proteins. Nat Struct Biol. 2003 Jan;10(1):59–69.
[5] Lockless SW, Ranganathan R. Evolutionarily conserved pathways of energetic connectivity in protein families. Science. 1999 Oct;286(5438):295–9.
[6] Shulman AI, Larson C, Mangelsdorf DJ, Ranganathan R. Structural determinants of allosteric ligand activation in RXR heterodimers. Cell. 2004 Feb;116(3):417–29.
[7] Socolich M, Lockless SW, Russ WP, Lee H, Gardner KH, Ranganathan R. Evolutionary information for specifying a protein fold. Nature. 2005 Sep;437(7058):512–8.
[8] Hyvärinen A, Karhunen J, Oja E. Independent component analysis. vol. 46. John Wiley & Sons; 2004.
[9] Bell AJ, Sejnowski TJ. An information-maximization approach to blind separation and blind deconvolution. Neural Comput. 1995 Nov;7(6):1129–59.
[10] Amari Si, Cichocki A, Yang HH, et al. A new learning algorithm for blind signal separation. Advances in neural information processing systems. 1996;9:757–763.
