# Supplementary material for: Evolution-Based Functional Decomposition of Proteins
Source: PLoS Comput Biol. 2016 Jun 2;12(6):e1004817. doi: 10.1371/journal.pcbi.1004817 (PMC4890866; doi:10.1371/journal.pcbi.1004817)
Supplement: S2 Text — The pySCA toolbox (v.6.1) is a distribution of SCA written in Python and comprises a library of functions (scaTools.py), four scripts to automate most calculations (scaAnnotateMSA.py, scaProcessMSA.py, scaCore.py, and scaSectorID.py), and several tutorials written using the interactive python notebook environment (iPython notebook). Here we describe installation of this toolbox, its usage via the scripts, and provide a list of classes and functions in the scaTools.py module with hyperlinks to access the main code documentation online. (PDF) [file pcbi.1004817.s002.pdf]

S2 Text. Description and usage of the pySCA toolbox

I. INSTALLATION AND USAGE

The pySCA toolbox (v.6.1) is a distribution of SCA written in Python and comprises a library of functions (scaTools.py), four scripts to automate most calculations (scaAnnotateMSA.py, scaProcessMSA.py, scaCore.py, and scaSectorID.py), and several tutorials written using the interactive python notebook environment (iPython notebook). Here we describe installation and usage of this toolbox via the scripts. See `http://reynoldsk.github.io/pySCA` for online instructions, updates, and further information.

A. Installation

The SCA toolbox may be downloaded at `https://github.com/reynoldsk/pySCA`. To use pySCA, it is essential to first download and install the following (free) packages:

(1) Anaconda Scientific Python - this package will install python and several libraries necessary for operation of the pySCA codes (NumPy, SciPy, iPython, and Matplotlib). See `https://store.continuum.io/cshop/anaconda/`

(2) Biopython - To install, either run from the python command line:
`>>>conda install biopython`
or download and install from `http://biopython.org/wiki/Main_Page`

In addition to these required packages, the following are highly recommended:

(1) The pfamseq.txt database - this file contains the phylogenetic annotations for PFAM sequences. This is necessary for annotation of PFAM alignments with taxonomic/phylogenetic information using the scaAnnotateMSA.py script. This is a rather large database. Download at `ftp://ftp.sanger.ac.uk/pub/databases/Pfam/current_release/database_files/`

(2) A robust pairwise sequence alignment program. Either of the following seem acceptable, in order of speed in our experience: (1) ggsearch - a component of the FASTA software package (`http://fasta.bioch.virginia.edu/fasta_www2/fasta_down.shtml`), or (2) needle - a component of the EMBOSS software package (`http://emboss.sourceforge.net/`.

(3) PyMol - A molecular graphics package. Necessary to use pySCA's automated structure mapping functions, and useful for mapping sectors to tertiary structure in general.

(4) mpld3 - a package that allows more interactive plot visualization in the ipython notebook environment. If not using, it will be necessary to comment out the line ”import mpld3” at the beginning of the tutorials.

Finally, after installation of these packages, it is necessary to make two user-specific edits to the pySCA files: (1) check the 'shebang' line (!#) at the start of the four pySCA scripts to appropriately reflect the path to your python installation, and (2) edit the following variables in the ”PATHS” section of scaTools.py to reflect the actual directory locations of these files on the installed computer systems:

(1) path2pfamseq = 'pfamseq.txt' *provide full path to location of this database file*

(2) path2structures = 'Inputs/' *provide full path to location of PDB structure files for analysis*

(3) path2pymol = 'Applications/MacPyMOL.app/Contents/MacOS/MacPyMOL' *provide full path to location of this application*

(4) path2needle = '/usr/local/bin' *provide full path to location of the needle executable, if EMBOSS is installed*

B. Scripts

The four scripts included with the pySCA toolbox automate the usage of the codes in scaTools.py to make the core calculations in SCA. The scripts are to be sequentially executed, and comprise (1) alignment annotation (optional), (2) alignment pre-processing, (3) core SCA calculations, and (4) sector identification, and outputs are stored in a user-specified database for later analysis in ipython notebook or optionally output as a MATLAB workspace for further analysis in that environment.

1. scaAnnotateMSA.py - Alignment annotation

The scaAnnotateMSA.py script reads in a FASTA formatted multiple sequence alignment and provides utilities to automatically annotate sequence headers with taxonomic information. These headers are used in interpreting the mapping between positional coevolution and the corresponding sequence divergences in the alignment (e.g. Fig. 6 of main text). Currently, this script supports two different alignment sources: (1) PFAM alignments, for which annotations are extracted from the pfamseq.txt taxonomy database, and (2) BLAST-derived alignments, for which annotations are added using the NCBI Entrez utilities provided in BioPython. For the latter, it is necessary to supply a list of gi numbers as an additional argument. Note that for both PFAM and NCBI utilities, the process of sequence annotation is rather slow (on the order of hours for typical alignment sizes). The annotated alignment is written out to disk for use in subsequent scripts. Below are two example usages, the first for a PFAM alignment of the DHFR family (`PF00186_full.fasta`), and the second (`DHFR_PEPM3.fasta`) for a custom alignment made using PSI-BLAST followed by the PROMALS-3D automated multiple alignment program:

```
>>> ./annotate_MSA.py Inputs/PF00186_full.fasta -o Outputs/PF00186_full.an -a 'pfam'
>>> ./annotate_MSA.py Inputs/DHFR_PEPM3.fasta -o Outputs/DHFR_PEPM3.an -a 'ncbi' -g Inputs/DHFR_PEPM3.gis
```

where flag `-o` specifies the output alignment name, flag `-a` specifies the alignment type (`'pfam'` or `'ncbi'`), and flag `-g` specifies the file with the list of gi numbers (for non-PFAM alignments only). See the header of scaAnnotateMSA.py script or associated html help page for additional details.

2. scaProcessMSA.py - alignment pre-processing

The scaProcessMSA.py script carries out pre-processing of alignments, including trimming of both positions and sequences with excessive gaps, applying sequence weights to remove trivial over-representation of closely related sequences, and if desired, sub-sampling of sequences to reduce compute times. The script accepts a FASTA formatted sequence alignment, PDB structures or sequences of a reference sequence, and a number of optional parameters detailed in the script header. The results are saved in a database for further analysis with an option to write out results to a MATLAB workspace. An example usage for the annotated G protein PFAM alignment (`PF00071_full.an`):

```
>>> ./scaProcessMSA.py Inputs/PF00071_full.an -s 5P21 -c A -f 'Homo sapiens' -m
```

where the flag `-s` species the PDB file of the reference sequence, `-c` species the chain ID, flag `-f` specifies the species corresponding to the reference sequence (this speeds identification of the reference sequence in the alignment), and optional flag `-m` provides all output variables in a MATLAB structure written to a workspace (`PF00071_full.mat`) in addition to the default python database (`PF00071_full.db`). See the header of scaProcessMSA.py or html help page for additional details.

3. scaCore.py - the core calculations

The scaCore.py script carries out the core calculations in SCA, including sequence similarities, positional conservation and coevolution, eigenvalue decomposition of the coevolution matrix, and the eigenvalue decomposition of randomization trials. Typical usage:

```
>>> ./scaCore.py PF00071_full.db -m
```

where the input is the database produced by scaProcessMSA.py, and the optional flag `-m` provides all outputs as another structure variable written to the MATLAB workspace `PF00071_full.mat` in addition to the python database. See the header of scaCore.py or html help page for additional details and options.

4. scaSectorID.py

The scaSectorID.py script carries out various steps for sector identification, including definition of number of significant eigenmodes (k_{max}), rotation of eigenvectors by ICA, and definition of the amino acid positions that contribute to each of the k_{max} independent components. Typical usage:

```
>>> ./scaSectorID.py PF00071_full.db -m
```

where the input is the database produced by scaCore.py, and the optional flag `-m` provides all outputs as another structure variable written to the MATLAB workspace `PF00071_full.mat` in addition to the python database. See the header of scaSectorID.py or html help for additional details and options.

II. The `scaTools.py` functions

Here we provide a list of classes and functions in the scaTools module as an introduction to the package; hyperlinks provide access the main code documentation online.

A. Multiple sequence alignment processing and annotation

scaTools.readAlg
: Read in a multiple sequence alignment in fasta format, and return the headers and sequences.

scaTools.AnnotPfam
: Phylogenetic annotation of a Pfam alignment (in fasta format) using information from the PFAM database pfamseq.txt. The output is a fasta file containing phylogenetic annotations in the header (to be parsed with "|" as a delimiter).

scaTools.clean_al
: Replaces any character that is not a valid amino acid by a gap.

scaTools.MSAsearch
: Identify the sequence in the alignment that most closely corresponds to the species of a specified reference sequence (often the sequence associated with a PDB structure), and return its index.

scaTools.chooseRefSeq
: This function chooses a default reference sequence if none is given by taking the sequence which has the mean pairwise sequence identity closest to that of the entire alignment.

scaTools.makeATS
: If specified, truncate the alignment to the structure (assumes MSAsearch has already been executed to identify the reference sequence (iref)) and produce a mapping (ats) between alignment positions and the positions in the reference sequence.

scaTools.lett2num
: Translates an alignment from a representation where the 20 natural amino acids are represented by letters to a representation where they are represented by the numbers 1,...,20, with any symbol not corresponding to an amino acid represented by 0.

scaTools.alg2bin

Translate an alignment of size M x L where the amino acids are represented by numbers between 0 and N_{aa} (obtained using lett2num) to a sparse binary array of size M x (N_{aa} x L). This gives the alignment representation shown in Fig. 1C

scaTools.seqWeights

Compute sequence weights for an alignment where the weight of a sequence is the inverse of the number of sequences in its neighborhood, defined as the sequences with sequence similarity below max_seqid (default = 0.8). The sum of the weights defines an effective number of sequences.

scaTools.filterSeq

Filter the alignment to remove sequences with more than max_fracgaps (default = 0.2)

scaTools.filterPos

Truncate the positions of an input alignment to reduce gaps, taking into sequence weights into account when computing amino acid frequencies.

scaTools.randSel

Random selection of Mtot sequences, drawn with weights and without replacement. The seed for the random number generator is fixed to ensure reproducibility.

scaTools.weighted_rand_list

Generate a random list of at most Nmax elements with weights (numpy array) but without replacement. Called by randSel.

scaTools.weighted_rand_sel

Generate a random index with probability given by input weights. Called by weighted_rand_list.

B. Basic Math and Statistical Functions

scaTools.freq

Compute amino acid frequencies for a given alignment, taking sequence weights into account.

scaTools.eigenVect

Return the eigenvectors and eigenvalues, ordered by decreasing values of the eigenvalues, for a real symmetric matrix M. The sign of the eigenvectors is fixed so that the mean of its components is non-negative.

scaTools.svdss

Singular value decomposition (SVD) for sparse matrices (top k components). The singular values are ordered by decreasing values, the sign of the singular vectors is fixed, For a matrix X, the SVD is defined as $X = U\Sigma V^T$.

scaTools.basicICA

Basic ICA algorithm, based on work by Bell and Sejnowski (**?**), and as described in S1 Text, Eq. 17.

scaTools.rotICA

ICA rotation (using basicICA) with default parameters and normalization of outputs.

C. The core SCA calculations

scaTools.seqSim

Take an MxL alignment (converted to numeric representation using lett2num) and compute a MxM matrices of sequence similarities, without sequence weights, with sequence weights, or with sequence and position weights.

scaTools.posWeights

Compute single-site measures of conservation D_i (S1 Text, Eq. 4) and D_i^a (S1 Text, Eq. 3), and the sca position weights, $\frac{\partial D_i^a}{\partial f_i^a}$ (S1 Text, Eq. 7)

scaTools.seqProj

Compute three different projections of the sequences based on eigenvectors of the sequence similarity matrix.

scaTools.scaMat

Computes the SCA matrix.

D. Mapping/projection between sequence and positional correlations

scaTools.projUica
: Compute the projection of one alignment on the top ICs of the sequence space of another. This is useful to compare the sequence space of one alignment to another.

scaTools.projAlg
: Dimension reduction of the three-dimensional alignment array x_{si}^{a} to a two-dimensional matrix x_{si} using a projection matrix, as described in S1 Text, Eq. 19-20.

scaTools.projUpica
: Compute the projection of an alignment on the kpos ICA components of the SCA matrix of another. This is useful to compare the sequence space (as projected by the positional correlations) of one alignment to another.

E. Sector Analysis

scaTools.sizeLargestCompo
: Compute the size of the largest component of a graph given its adjacency matrix.

scaTools.numConnected
: Calculates the number of positions in the largest connected component for groups of positions i with $V_p[i,k] > eps$ and $V_p[i,k] > V_p[i,kk]$, for $kk! = k$ and eps in eps_list. Useful for looking evaluating the physical connectivity of different sectors or sub-sectors.

scaTools.chooseKpos
: Given the eigenvalues of the SCA matrix, and the eigenvalues for the set of randomized matrices, return the number of significant eigenmodes.

scaTools.icList
: Produces a list of positions contributing to each independent component (IC) above a defined statistical cutoff. Any positions above the cutoff on more than one IC are assigned to one IC based on which group of positions to which it shows a higher degree of coevolution.

scaTools.randAlg
: Generate a random alignment with Mseq sequences based on the frequencies f_i^a of amino acids with a = 0,1,...,Naa (0 for gaps).

scaTools.randomize
: Randomize the alignment while preserving the frequencies of amino acids at each position and compute the resulting eigenvalue spectrum of the SCA matrix.

F. Direct Contact Analysis

Though not described in the main text, the toolbox also includes functions for predicting contacts using one implementation of the direct contact analysis (as described in (**?**)).

scaTools.Pair
: A class for a pair of amino acid positions, attributes: pair of amino acid residues, the direct information between the positions, and physical distance between the residues.

scaTools.directInfo
: Calculate direct information as in the Direct Coupling Analysis (DCA) method proposed by M. Weigt et al. (**?**).

scaTools.dirInfoFromJ
: Direct information from the matrix of couplings J_{ij} (called by the function directInfo).

scaTools.truncDiag
: Set to 0 the elements of a matrix M up to a distance dmax from the diagonal.

scaTools.Secton
: A class for a sectons - small groups of (usually contacting) amino acids identified by direct information.

G. PDB processing

scaTools.pdbSeq
Extract sequence, position labels and matrix of distances from a PDB file.

scaTools.writePymol
Write basic a pymol script for displaying sectors and exporting an image.

scaTools.figStruct
Make and display an image of the sectors (within a python notebook).

H. Output and Plotting

scaTools.Unit
A generic Unit class - can contain sectors, sequence families, etc. Attributes: list of items, a color code, name or description, and vector describing the member items.

scaTools.figWeights
2d scatter plot with color indicating weight.

scaTools.figColors
Color code for figUnits

scaTools.figUnits
2d scatter plot, with color coding as specified by 'units', which must be a list of elements in the class Unit. Useful to make plots color coded by sequence annotation, or sector definition.

scaTools.figMapping
Function that automates finding the top k_{pos} independent components, projection, and plotting. Useful to get a representation of the sectors/subfamilies mapping for a given kpos.

scaTools.MultiBar
Multiple bar diagram (plots contributions to each bar from different elements in x as different colors). This can be useful if you would like to inspect how sector positions are distributed among independent components/eigenmodes.

scaTools.singleBar
Single bar diagram, called by MultiBar.

scaTools.cytoscapeOut
Output tab-delimited text that can be read in by cytoscape. The goal is to enable graph representations of the SCA couplings, where residues are nodes, and couplings are edges.
