# Supplementary material for: Evolution-Based Functional Decomposition of Proteins
Source: PLoS Comput Biol. 2016 Jun 2;12(6):e1004817. doi: 10.1371/journal.pcbi.1004817 (PMC4890866; doi:10.1371/journal.pcbi.1004817)
Supplement: S3 Text — We provide tutorials to describe the sector identification process for four protein families, with the goal of illustrating several features of the SCA. The tutorials are additionally available online as html files, and can be downloaded as interactive python notebooks for use with the pySCA toolbox (https://github.com/reynoldsk/pySCA). (PDF) [file pcbi.1004817.s003.pdf]

S3 Text. Tutorials

We provide tutorials to describe the sector identification process for four protein families, with the goal of illustrating several features of the SCA. The tutorials are additionally available online as html files, and can be downloaded as interactive python notebooks for use with the pySCA toolbox (`https://github.com/reynoldsk/pySCA`).

In these tutorials, the alignment processing, SCA calculations and projection steps are pre-computed using three python scripts: scaProcessMSA.py, scaCore.py and scaSectorID.py. The tutorials load the results of these calculations (stored as python pickle dictionaries) and demonstrate how to analyze the resulting data. As each tutorial is designed to illustrate a different aspect of the analyses, we suggest completing the tutorials in the order listed below.

G-proteins (Alignment: PF00071)
This tutorial walks through the analysis for the G-protein (small GTPase, Ras-like) family as described in the main text, and provides an illustration of the basic SCA methodology. We show how to compute basic statistics for the starting sequence alignment, single-site conservation values, and the SCA matrix. The SCA matrix is analyzed by spectral decomposition, sectors are defined, and we explain how to analyze the sequence-to-position space mapping.

S1A serine proteases (Custom alignment)
The S1A tutorial provides an example where each independent component corresponds to a single sector. When mapped to the structure, each sector forms a physically contiguous group of residues. The sequence-position space projection shows that the top three sectors are associated with the divergence of three different functional properties: enzyme catalytic activity, enzyme specificity, and vertebrate/invertebrate enzymes.

Dihydrofolate Reductase (DHFR) (Alignment: PF00186)
In this tutorial, we compare results of the SCA for two different alignments: a PFAM alignment (PF00186) and an independent manually curated alignment constructed using a custom database of orthologous sequences (DHFRPEPM3.an). Despite differences in the construction, sequence distribution and size of the two alignments, the sector definition is remarkably consistent: in both cases we arrive at a single sector assembled from six independent components. In both cases, the six independent components reflect the divergence of the sector along phylogenetic lines.

Beta-lactamase (Alignment: PF13354)
The Beta-lactamase tutorial illustrates the process of sector identification for a sequence alignment containing clear sequence groups or clades of higher similarity. In this instance, we see that the application of sequence weights helps to ”smooth out” the sequence distribution. We find two sectors: sector 1 consists of IC2, and is associated with a uniform distribution of sequences - indicating that this sector is a property of the entire alignment. Structural mapping shows that this sector is associated with the core catalytic machinery of the beta-lactamase. Sector 2 is composed of the remaining 5 ICs, and is subdivided along phylogenetic lines. Sector 2 forms a structural ”shell” around sector 1, and the divergence of this sector in different sequence families suggests that it may ”tune” the activity of the Beta-lactamase in different functional or phylogenetic subgroups.

SCA_G

January 3, 2016

0.1 SCA6.0 - The G protein family

Summary: This script describes the basic flow of analytical steps in SCA6.0, using the G-protein (small GTPase, Ras-like) family as an example (PFAM PF00071). The analysis consists of five steps, which directly follow from the accompanying publication (Rivoire et al, "An Evolution-Based Functional Decomposition of Natural Proteins"):

1) Alignment processing and composition. We begin by analyzing the composition of the mulitple sequence alignment: what is the number of effective sequences, and how are the sequences structured into families? For the G-protein family, the PFAM alignment sequence space is well-sampled and fairly homogeneous (unstructured), as evidenced by the fact that overall alignment sequence similarity shows a unimodal distribution near 25%.

2) First-order statistics: position-specific conservation. Next, we examine overall positional conservation for the sequence alignment. This shows the expected result, that a handful of positions are strongly conserved.

3) Second-order statistics: conserved correlations. Plots of the SCA matrix ($\tilde{C_{ij}}$), the associated eigenvectors and eigenspectrum, and the corresponding independent components (IC). We choose the number of significant eigenmodes, $k^* = 4$, by comparison of the eigenspectrum of $\tilde{C_{ij}}$ to that for a collection of 10 randomized alignments.

4) Sector decomposition. Sector definition based on the top k^* ICs. We begin by fitting the top k^* statistically significant ICs to a t-distribution, and then identify the amino acid positions contributing to the top five percent of the corresponding cumulative density function. We then construct a sub-matrix of $\tilde{C_{ij}}$ that contains only the selected top-scoring positions for the k^* ICs, ordered by their degree of contribution to each IC. This plot is used to choose sector assignments. For the g-protein family, we define two sectors, sector 1 composed of ICs 1,2, and 3, and sector 2 composed of IC 4. Related to Figs. 4 and 5 of the main text.

5) Sequence subfamilies and the basis of sector hierarchy. We relate the two sectors (and underlying ICs) to the pattern of divergence between amino acid sequences. To do this, we make a mapping between sequence space and positional correlations using singular value decomposition. We see that the amino acid positions associated with IC1 and IC2 differentiate between different g-protein subclasses, suggesting that these regions might tune allosteric regulation in a subclass specific way.

Prior to running this tutorial, the core calculation scripts must be executed from the command line as follows: >> ./scaProcessMSA.py Inputs/PF00071_rd2.an -s 5P21 -c A -f 'Homo sapiens' -t -n >> ./scaCore.py Outputs/PF00071_rd2.db >> ./scaSectorID.py Outputs/PF00071_rd2.db

Note that we supply pre-annotated alignments for all tutorial scripts *(the annotate_pfMSA step is slow, and should only be run once).*

O.Rivoire, K.Reynolds and R.Ranganathan 1/2015

In [1]:
```
%matplotlib inline
from __future__ import division

import os
import time
import matplotlib.pyplot as plt
import numpy as np
```

```
import copy
import scipy.cluster.hierarchy as sch
from scipy.stats import scoreatpercentile
import matplotlib.image as mpimg
from IPython.display import display
from IPython.display import Image
from Bio.Seq import Seq
from Bio import motifs
import colorsys
import scaTools as sca
import mpld3
import cPickle as pickle
from optparse import OptionParser

if not os.path.exists('Outputs/'): os.makedirs('Outputs/')
```

To begin, we read in the results of the above three scripts (scaProcessMSA, scaCore and scaSectorID), stored as three dictionaries in the database PF00071_full.db. To see what variables are stored in each dictionary, use the command dictionary.keys( ), e.g.:

```
>>> print Dseq.keys()
```

```
In [2]: db = pickle.load(open('Outputs/PF00071_rd2.db','rb'))
        Dseq = db['sequence']  #the results of scaProcessMSA
        Dsca = db['sca']       #the results of scaCore
        Dsect = db['sector']   #the results of scaSectorID
```

0.1.1 I. Alignment processing and composition

First, we print out a few statistics describing the alignment:

```
In [3]: print("After processing, the alignment size is %i sequences and %i positions" % \
              (Dseq['Nseq'], Dseq['Npos']))
        print("With sequence weights, there are %i effective sequences" % (Dseq['effseqs']))
```

```
After processing, the alignment size is 4974 sequences and 158 positions
With sequence weights, there are 3366 effective sequences
```

To examine alignment composition, we plot a histogram of all pairwise sequence identities *(left panel)* and a global view of the sequence similarity matrix (defined by $S \equiv \frac{1}{L} X X^{\top}$) *(right panel)*. The data show that the alignment is described by a nearly homogeneous distribution of sequence identities with a mean value of about 25%.

```
In [4]: # List all elements above the diagonal (i<j):
        listS = [Dsca['simMat'][i,j] for i in range(Dsca['simMat'].shape[0]) \
                 for j in range(i+1, Dsca['simMat'].shape[1])]
        #Cluster the sequence similarity matrix
        Z = sch.linkage(Dsca['simMat'],method = 'complete', metric = 'cityblock')
        R = sch.dendrogram(Z,no_plot = True)
        ind = map(int, R['ivl'])
        #Plotting
        plt.rcParams['figure.figsize'] = 9, 4
        plt.subplot(121)
        plt.hist(listS, Dseq['Npos']/2)
        plt.xlabel('Pairwise sequence identities', fontsize=14)
        plt.ylabel('Number', fontsize=14)
        plt.subplot(122)
        plt.imshow(Dsca['simMat'][np.ix_(ind,ind)], vmin=0, vmax=1); plt.colorbar();
```

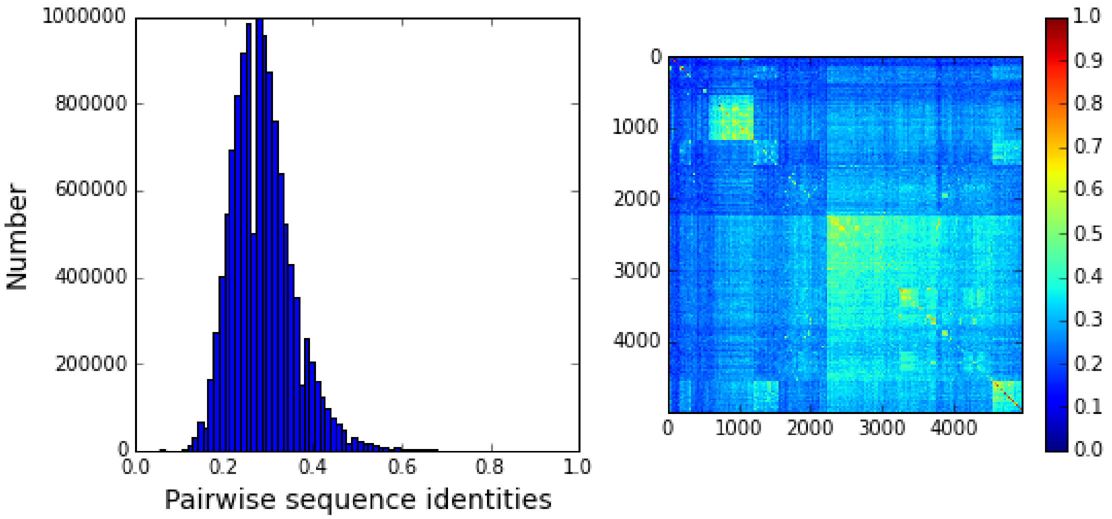

Sequence annotations: We construct a dictionary of phylogenetic annotations (parsed from the sequence headers) and check the representation of sequences in the top taxonomic levels. For each level, we print a list of taxonomic classes, and the number of sequences found for each.

In [5]:
```
#construct a dictionary of phylogenetic groups
annot = dict()
for i, h in enumerate(Dseq['hd']):
    hs = h.split('|')
    annot[hs[0]] = sca.Annot(hs[1], hs[2], hs[3].replace('.',''))

# Most frequent taxonomic groups:
atleast = 10
for level in range(4):
    descr_list = [a.taxo.split(',')[level] for a in annot.values() \
                  if len(a.taxo.split(',')) > level]
    descr_dict = {k:descr_list.count(k) for k in descr_list \
                  if descr_list.count(k)>=atleast}
    print '\n Level %i:' % level
    print descr_dict
```

```
Level 0:
{'Eukaryota': 4879, 'Bacteria': 74}

 Level 1:
{'Ichthyosporea': 22, 'stramenopiles': 159, 'Alveolata': 530, 'Oxymonadida': 14, 'Choanoflagellida': 47
'Viridiplantae': 192, 'Parabasalia': 321, 'Bacteroidetes': 18, 'Amoebozoa': 510, 'Fungi': 702,
'Metazoa': 2048, 'Cyanobacteria': 20, 'Diplomonadida': 32, 'Heterolobosea': 143,
Euglenozoa': 148, 'Proteobacteria': 27}

 Level 2:
{'Schizopyrenida': 143, 'Blastocystis': 20, 'Trichomonadida': 320, 'Dikarya': 622,
'Bacillariophyta': 23, 'Echinodermata': 46, 'Porifera': 105, 'Capsaspora': 22, 'Apicomplexa': 89,
'Mycetozoa': 255, 'Chytridiomycota': 15, 'Fungi incertae sedis': 17, 'Pelagophyceae': 25,
 'Codonosigidae': 22, 'Streptophyta': 137, 'Perkinsea': 32, 'Hexamitidae': 32,
 'Salpingoecidae': 25, 'Archamoebae': 255, 'Chordata': 985, 'Platyhelminthes': 89, 'Oomycetes': 67,
```

```
 Kinetoplastida': 148, 'Arthropoda': 520, 'Microsporidia': 46, 'Placozoa': 44, '
 Gammaproteobacteria': 10, 'Ciliophora': 408, 'Nematoda': 175, 'PX clade': 24, 'Chlorophyta': 55,
 'Cnidaria': 78}

 Level 3:
{'Vahlkampfiidae': 143, 'Trebouxiophyceae': 12, 'Demospongiae': 105, 'Albuginales': 22,
'Trypanosomatidae': 148, 'Ascomycota': 471, 'Early diverging fungal lineages': 17, 'Phaeophyceae': 23,
'Perkinsida': 32, 'Chytridiomycetes': 15, 'Trichoplax': 44, 'Unikaryonidae': 17, 'Tunicata': 122,
'Monosiga': 22, 'Peronosporales': 45, 'Intramacronucleata': 408, 'Crustacea': 73, 'Eleutherozoa': 46,
'Trichomonadidae': 320, 'Chlorophyceae': 19, 'Cephalochordata': 54, 'Enterocytozoonidae': 11,
 'Aureococcus': 25, 'Coccidia': 37, 'Salpingoeca': 25, 'Chromadorea': 154,
  'Coscinodiscophyceae': 16, 'Basidiomycota': 151, 'Enoplea': 21, 'Entamoebidae': 255,
  'Trematoda': 85, 'Anthozoa': 76, 'Dictyosteliida': 253, 'Embryophyta': 136, 'Mamiellophyceae': 24,
  'Hexapoda': 402, 'Aconoidasida': 52, 'Craniata': 809, 'Chelicerata': 45, 'Giardiinae': 32}
```

Based on this, we select taxonomic groups and associate them to colors for representation. We choose broad taxonomic groups that are well-represented in the alignment (corresponding to Level 1). To see a complete legend that maps numeric codes to color, use:

```
>>> sca.figColors()
```

```
In [6]: phylo = list();
        fam_names = ['Metazoa', 'Amoebozoa', 'Viridiplantae', 'Fungi',\
                     'Alveolata', 'Parabasalia']
        col = (0, 0.6, 0.38, 0.18, 0.8, 0.5)
        #Metazoa = red, Amoebozoa = yellow, Viridiplantae = green,
        #Fungi = cyan, Alveolata = blue, Parabasalia = purple
        for i,k in enumerate(fam_names):
            sf = sca.Unit()
            sf.name = fam_names[i].lower()
            sf.col = col[i]
            sf.items = [j for j,q in enumerate(Dseq['hd'])  if sf.name in q.lower()]
            phylo.append(sf)
```

We also attempt to annotate the sequences by their declared sub-class of G protein - Ras, Rab, Rac, and Rho. These annotations are simply parsed from the header, and could contain mis-assignments.

```
In [7]: gprot_names = ['Ras', 'Rab', 'Rac','Rho']
        gprot_classes = list()
        col = (0, 0.65, 0.15, 0.38)
        #Ras=light blue, Rab = orange, Rac=yellow, Rho=dark blue
        for c,k in enumerate(gprot_names):
            gp = sca.Unit()
            gp.col = col[c]
            gp.name = k
            gp.items = [i for i,h in enumerate(Dseq['hd']) if k in h]
            gprot_classes.append(gp)
```

To examine the relationship between global sequence similarity, phylogeny, and functional sub-class, we plot the top six independent components of the sequence correlation matrix (including sequence weights). In these plots, each point represents a particular sequence, and the distance between points reflects global sequence identity. In the top row each point (sequence) is color coded by phylogenetic annotation, in the bottom row, they are color-coded by g-protein class.

```
In [8]: plt.rcParams['figure.figsize'] = 9, 8
        U = Dsca['Uica'][1]
```

```python
pairs = [[2*i,2*i+1] for i in range(3)]
for k,[k1,k2] in enumerate(pairs):
    plt.subplot(2,3,k+1)
    sca.figUnits(U[:,k1], U[:,k2], phylo)
    plt.xlabel(r"$IC_{seq}^{%i}$"%(k1+1), fontsize=16)
    plt.ylabel(r"$IC_{seq}^{%i}$"%(k2+1), fontsize=16)
    plt.subplot(2,3,k+4)
    sca.figUnits(U[:,k1], U[:,k2], gprot_classes)
    plt.xlabel(r"$IC_{seq}^{%i}$"%(k1+1), fontsize=16)
    plt.ylabel(r"$IC_{seq}^{%i}$"%(k2+1), fontsize=16)
plt.tight_layout()
```

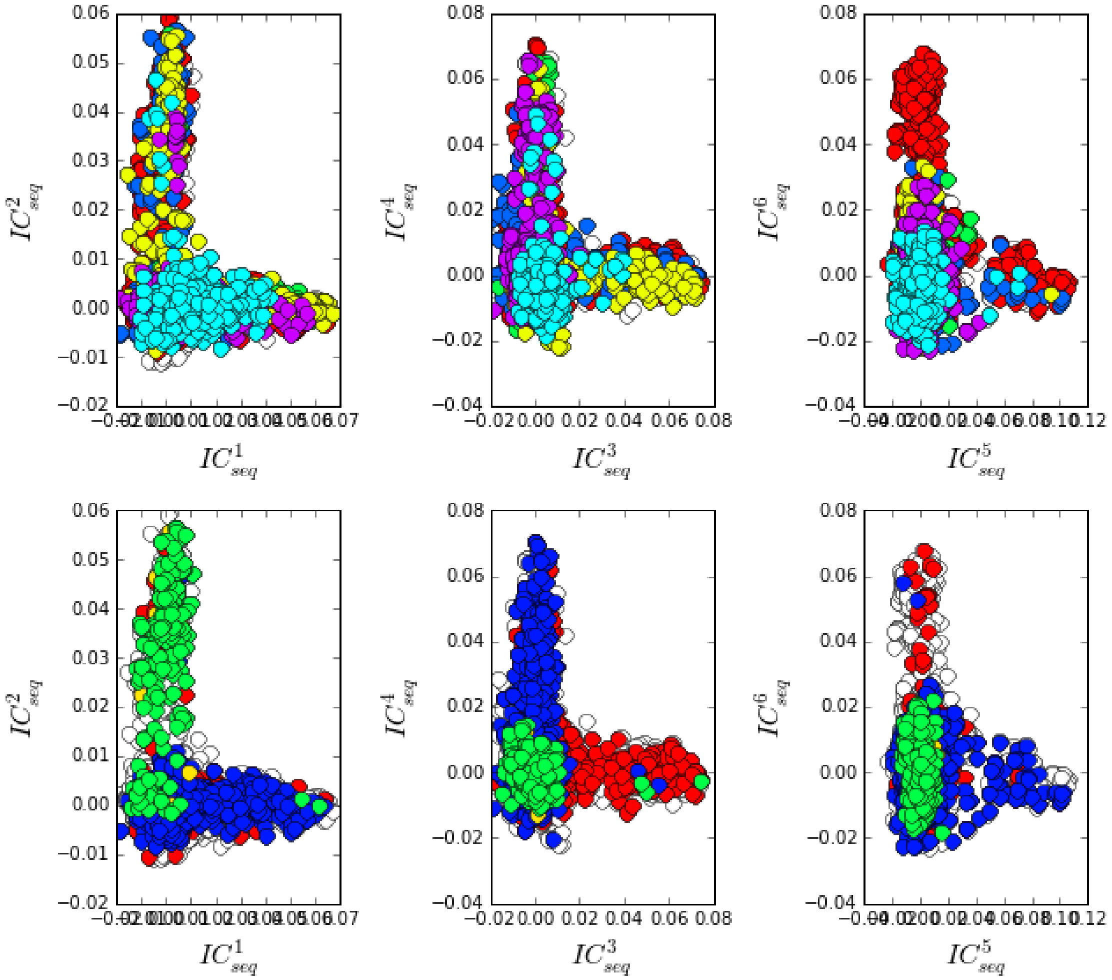

The data show a mixed distribution of phylogenetic groups along modes 1-5. A subset of metazoan sequences emerges along the mode six, IC^6_{seq}. In contrast, the top modes of the sequence similarity matrix do seem to correspond to functional G protein subclasses. For example, the Rho proteins *(green)* emerge along IC^2_{seq} , the Ras proteins *(red)* along IC^3_{seq}, and a subset of Rabs *(blue)* along IC^4_{seq} and IC^5_{seq} and a subset of Ras proteins along IC^6_{seq}. Many G-protein paralogs (reflecting different subclasses) can be found in each type of organism, and thus the global pattern of sequence divergence is distinct from phylogeny.

0.1.2 II. First-order statistics: position-specific conservation.

Plot the position-specific conservation values for each g-protein position. D_i is calculated according to equation S4 (supplemental information).

In [9]:
```
fig, axs = plt.subplots(1,1, figsize=(9,4))
xvals = [i+1 for i in range(len(Dsca['Di']))]
xticks = [0,45,95,144]
plt.bar(xvals,Dsca['Di'], color='k')
plt.tick_params(labelsize=11); plt.grid()
axs.set_xticks(xticks);
labels = [Dseq['ats'][k] for k in xticks]
axs.set_xticklabels(labels);
plt.xlabel('Amino acid position', fontsize=18); plt.ylabel('Di', fontsize=18);
```

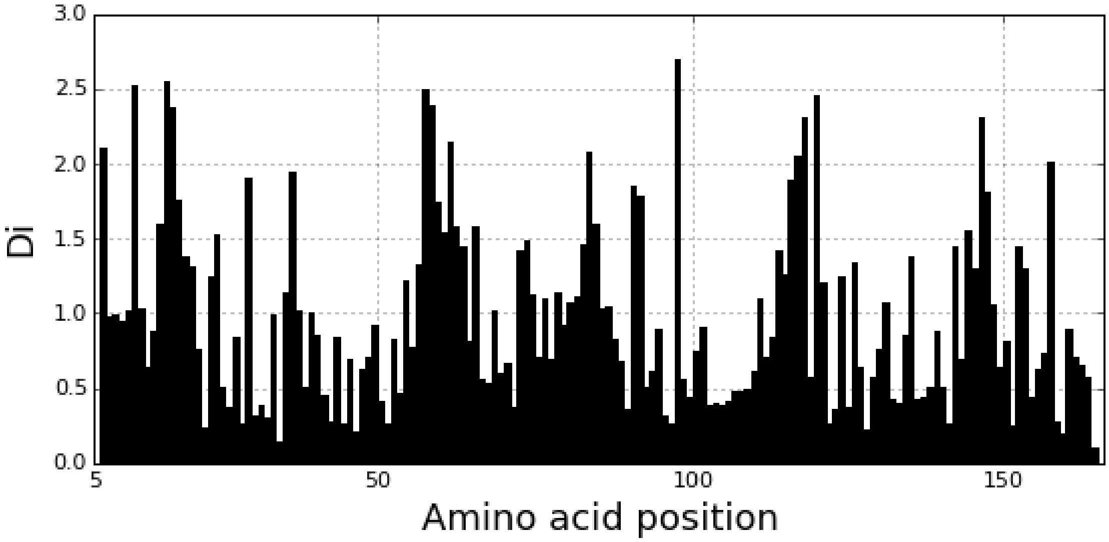

0.1.3 III. Second-order statistics: conserved correlations.

Plot the SCA correlation matrix ($\tilde{C_{ij}}$) computed according to Equations 4+5

In [10]:
```
plt.rcParams['figure.figsize'] = 13, 8
plt.imshow(Dsca['Csca'], vmin=0, vmax=1.4,interpolation='none',\
           aspect='equal')
```

Out[10]:
```
<matplotlib.image.AxesImage at 0x12bf49950>
```

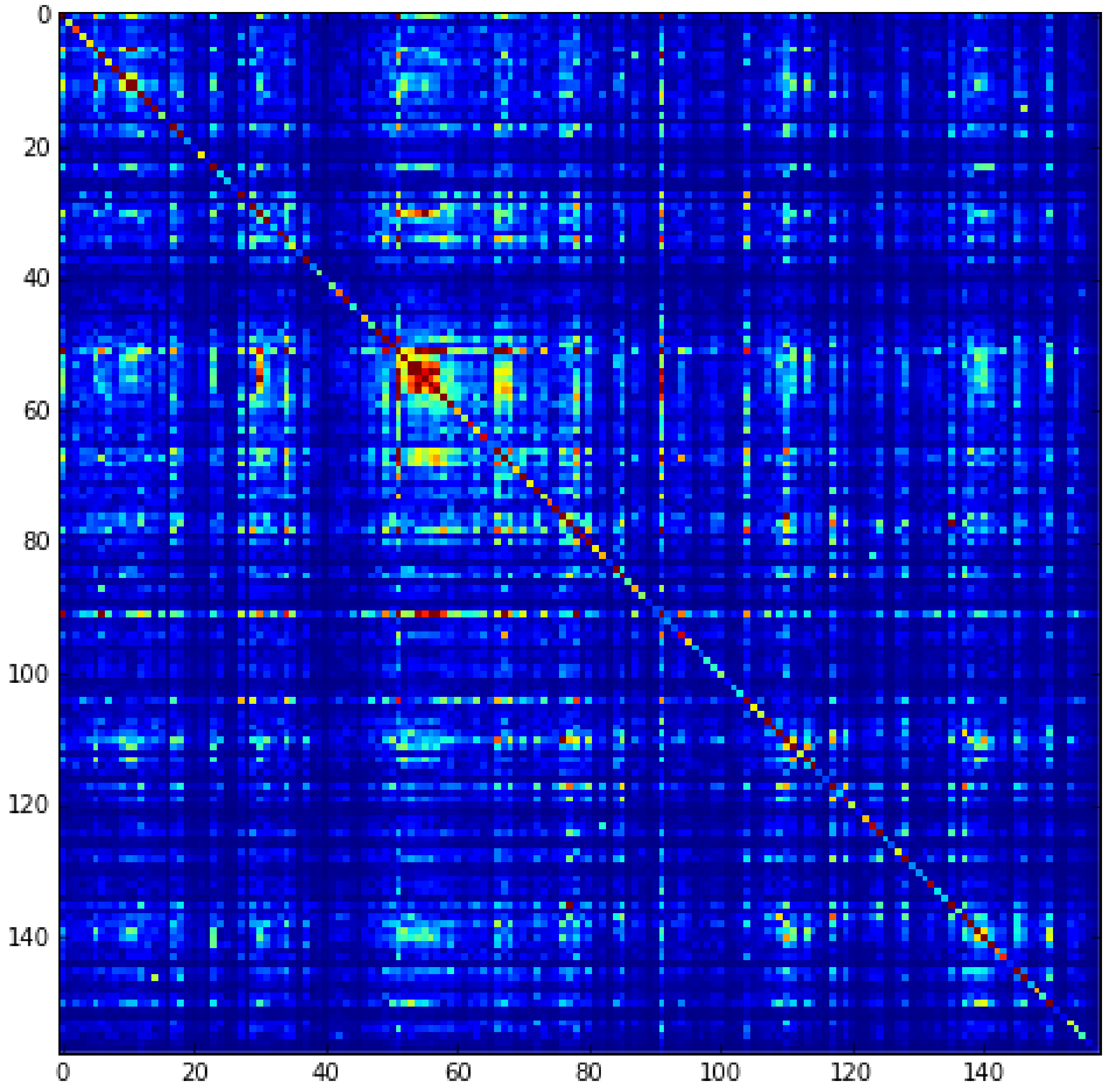

Plot the eigenspectrum of (1) the SCA positional coevolution matrix ($\tilde{C_{ij}}$) *(black bars)* and (2) 10 trials of matrix randomization for comparison. This graph is used to choose the number of significant eigenmodes ($k^* = 4$).

```
In [11]: plt.rcParams['figure.figsize'] = 9, 4
         hist0, bins = np.histogram(Dsca['Lrand'].flatten(), bins=Dseq['Npos'], \
                                    range=(0,Dsect['Lsca'].max()))
         hist1, bins = np.histogram(Dsect['Lsca'], bins=Dseq['Npos'], \
                                    range=(0,Dsect['Lsca'].max()))
         plt.bar(bins[:-1], hist1, np.diff(bins),color='k')
         plt.plot(bins[:-1], hist0/Dsca['Ntrials'], 'r', linewidth=3)
         plt.tick_params(labelsize=11)
         plt.xlabel('Eigenvalues', fontsize=18); plt.ylabel('Numbers', fontsize=18);
         print 'Number of eigenmodes to keep is %i' %(Dsect['kpos'])
         mpld3.display()
```

```
Number of eigenmodes to keep is 4
```

```
Out[11]: <IPython.core.display.HTML object>
```

Plot the top significant eigenmodes *(top row)* and associated independent components *(bottom row)*. The ICs are an optimally independent representation of the four different residue groups.

```
In [12]: plt.rcParams['figure.figsize'] = 9, 6
         pairs = [[0,1],[1,2],[2,3]]
         EVs = Dsect['Vsca']
         ICs = Dsect['Vpica']
         for k,[k1,k2] in enumerate(pairs):
             plt.subplot(2,3,k+1)
             plt.plot(EVs[:,k1], EVs[:,k2], 'ok')
             plt.xlabel("EV%i"%(k1+1), fontsize=16)
             plt.ylabel("EV%i"%(k2+1), fontsize=16)
             plt.subplot(2,3,k+4)
             plt.plot(ICs[:,k1], ICs[:,k2], 'ok')
             plt.xlabel("IC%i"%(k1+1), fontsize=16)
             plt.ylabel("IC%i"%(k2+1), fontsize=16)
         plt.tight_layout()
```

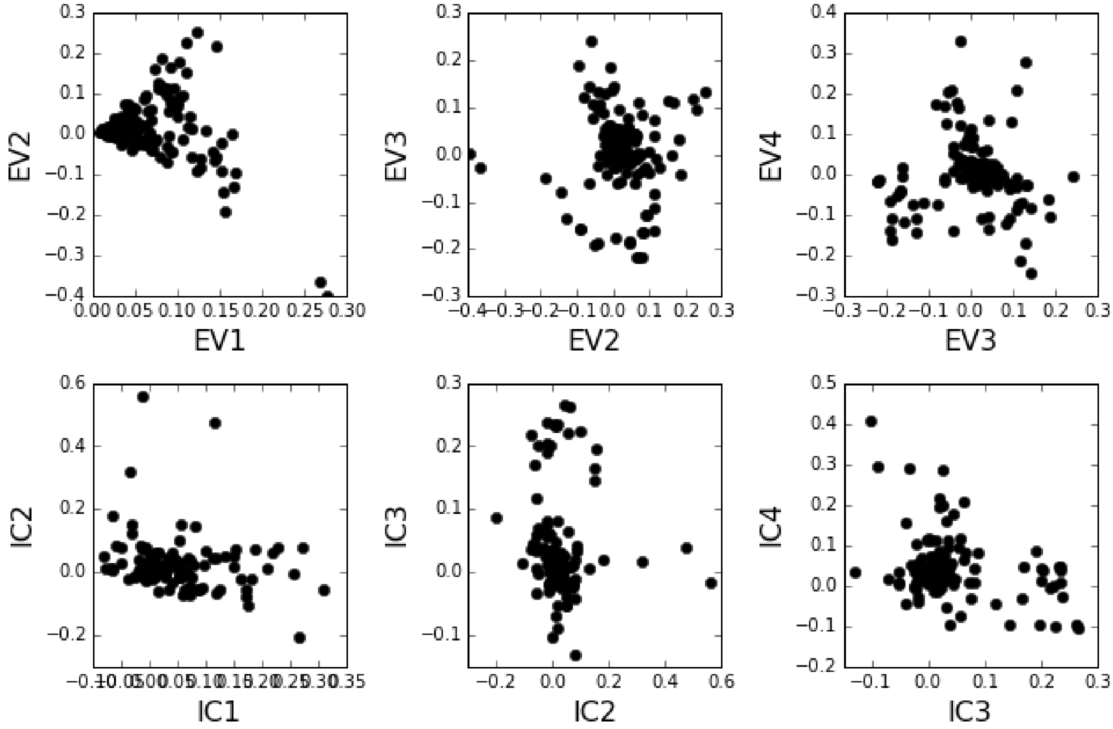

0.1.4 IV. Sector decomposition.

To define the positions with significant contributions to each of the independent components (ICs), we make a empirical fit for each IC to the t-distribution and select positions with greater than a specified cutoff on the CDF. We choose $p = 0.95$ as our cutoff. Note that since some positions might contribute significantly to more than one IC (an indication of non-independence of ICs), we apply a simple algorithm to assign such positions to one IC. Specifically, we assign positions to the IC with which it has the greatest degree of co-evolution.

The data indicate generally good fits for the top five ICs (also shown in supplemental figure S2), and we return the positions contributing to each IC in a format suitable for cut and paste into PyMol.

```
In [13]: plt.rcParams['figure.figsize'] = 8, 8

         Vpica = Dsect['Vpica']
         for k in range(Dsect['kpos']):
             iqr = scoreatpercentile(Vpica[:,k],75) - scoreatpercentile(Vpica[:,k],25)
             binwidth=2*iqr*(len(Vpica)**(-0.33))
             nbins=round((max(Vpica[:,k])-min(Vpica[:,k]))/binwidth)
             plt.subplot(Dsect['kpos'],1,k)
             h_params = plt.hist(Vpica[:,k], nbins)
             x_dist = np.linspace(min(h_params[1]), max(h_params[1]), num=100)
             plt.plot(x_dist,Dsect['scaled_pd'][k],'r',linewidth = 2)
             plt.plot([Dsect['cutoff'][k],Dsect['cutoff'][k]], [0,60], 'k--',linewidth = 1)
             plt.xlabel(r'$V^p_{%i}$'%(k+1), fontsize=14)
             plt.ylabel('Number', fontsize=14)

         for n,ipos in enumerate(Dsect['ics']):
             sort_ipos = sorted(ipos.items)
             ats_ipos = ([Dseq['ats'][s] for s in sort_ipos])
             ic_pymol = ('+'.join(ats_ipos))
             print('IC %i is composed of %i positions:' % (n+1,len(ats_ipos)))
             print(ic_pymol + "\n")
```

```
IC 1 is composed of 18 positions:
22+32+34+36+39+54+63+64+68+71+73+75+81+83+85+110+116+144

IC 2 is composed of 8 positions:
5+11+56+61+62+72+96+99

IC 3 is composed of 16 positions:
10+14+15+16+28+35+57+58+59+60+117+119+145+146+147+156

IC 4 is composed of 11 positions:
17+23+82+90+115+123+125+130+134+141+143

/Users/kreynolds/anaconda/lib/python2.7/site-packages/matplotlib/axes/_subplots.py:69: MatplotlibDepreca
  mplDeprecation)
```

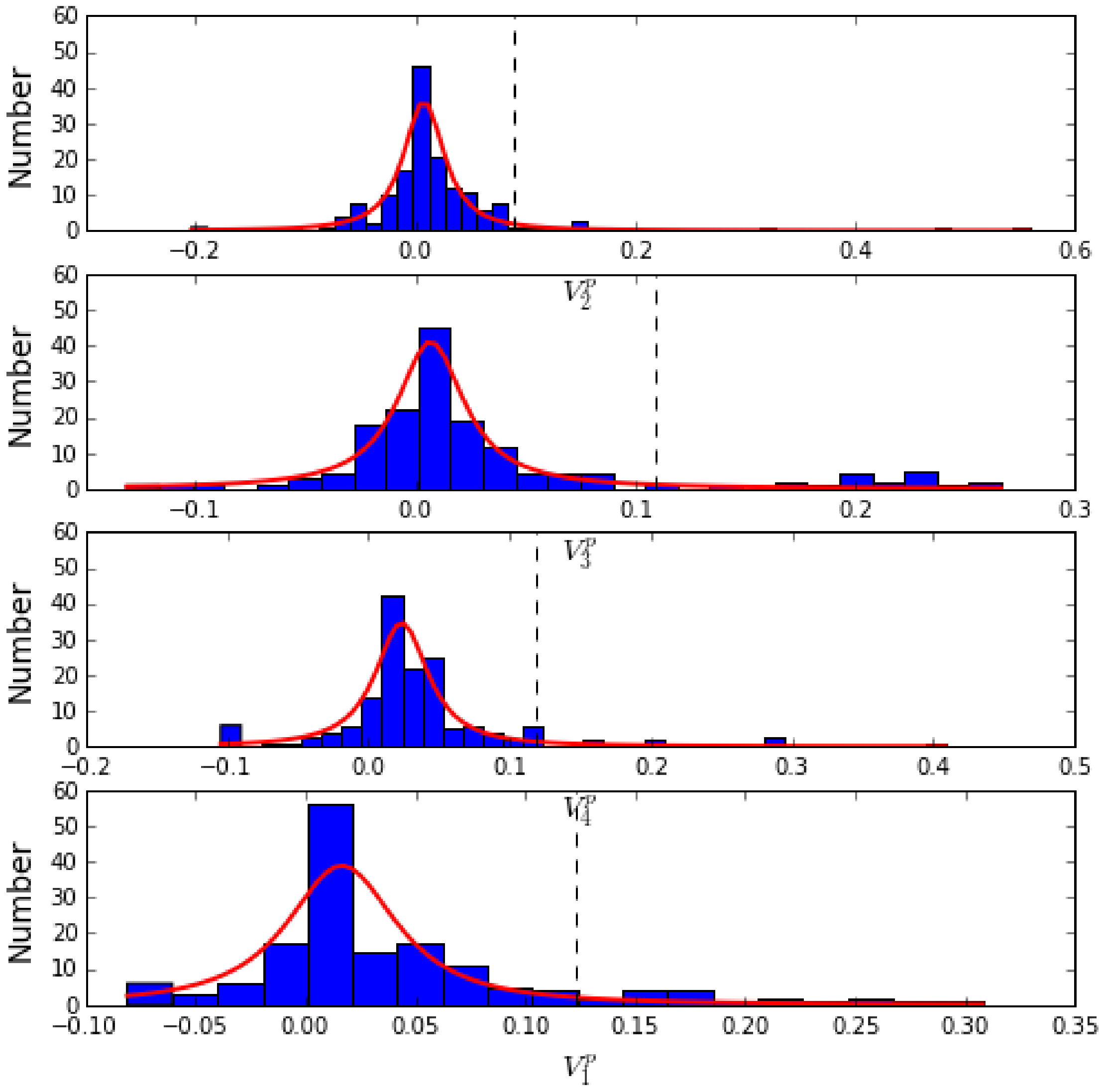

To define protein sectors, we examine the structure of the SCA positional correlation matrix with positions contributing to the top independent components (ICs) ordered by weight (*left panel*). This provides a basis to determine/interpret which ICs are truly statistically independent (defining an independent sector) and which represent hierarchical breakdowns of one sector. In this case, the data suggest that ICs 1, 2, and 3 have strong inter-IC correlations and should be considered a single sector, and IC4 shows little corrleation with other ICs, implying a distinct sector (see the dendrogram that follows). In the *right panel* the ICs are re-ordered to reflect this decomposition.

```
In [14]: #plot the SCA positional correlation matrix, ordered by contribution to the top ICs
         plt.rcParams['figure.figsize'] = 9, 9
         plt.subplot(121)
         plt.imshow(Dsca['Csca'][np.ix_(Dsect['sortedpos'], Dsect['sortedpos'])], \
                    vmin=0, vmax=2.2,interpolation='none',\
                    aspect='equal',extent=[0,sum(Dsect['icsize']),\
                                           0,sum(Dsect['icsize'])])
         line_index=0
```

```
for i in range(Dsect['kpos']):
    plt.plot([line_index+Dsect['icsize'][i],line_index+Dsect['icsize'][i]],\
             [0,sum(Dsect['icsize'])],'w', linewidth = 2)
    plt.plot([0,sum(Dsect['icsize'])],[sum(Dsect['icsize'])-\
            line_index,sum(Dsect['icsize'])-line_index],'w', linewidth = 2)
    line_index += Dsect['icsize'][i]

#define the new sector groupings - 3 total
sec_groups = ([0,1,2],[3])
sectors = list()
c = [0.66, 0]
for n,k in enumerate(sec_groups):
    s = sca.Unit()
    all_items = list()
    all_Vp = list()
    for i in k:
        all_items = all_items+Dsect['ics'][i].items
        all_Vp = all_Vp+list(Dsect['ics'][i].vect)
    svals = np.argsort(all_Vp)
    s.items = [all_items[i] for i in svals]
    s.col = c[n]
    sectors.append(s)

#plot the re-ordered matrix
plt.subplot(122)
line_index=0
sortpos = list()
for s in sectors:
    sortpos.extend(s.items)
plt.imshow(Dsca['Csca'][np.ix_(sortpos, sortpos)], vmin=0, vmax=2.2,\
           interpolation='none',aspect='equal',\
           extent=[0,len(sortpos),0,len(sortpos)])
for s in sectors:
    plt.plot([line_index+len(s.items),line_index+len(s.items)],\
             [0,len(sortpos)],'w', linewidth = 2)
    plt.plot([0,sum(Dsect['icsize'])],[len(sortpos)-line_index,\
                   len(sortpos)-line_index],'w', linewidth = 2)
    line_index += len(s.items)
plt.tight_layout()
```

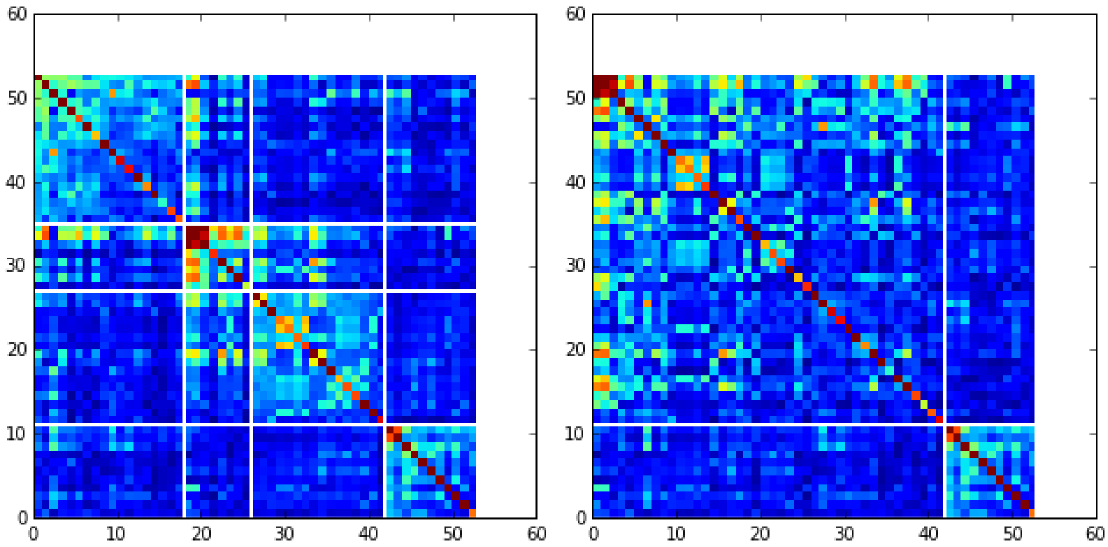

The below dendrogram diagrams the relationship between independent components. In this plot, solid lines represent physically contiguous structural units, and dashed lines indicate spatially fragmented groups of residues. We see that ICs 1,2,and 3 combine to form a single sector (sector 1), and that sector 2 (IC4) is more independent.

```
In [15]: i = Image(filename='figs/Gprot_sec_hier.png'); i
```

```
Out[15]:
```

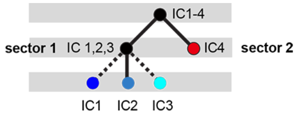

The assignments have clear physical consistency with the concept of sectors as functional, physically contiguous units in the protein structure (see also Figs.4-5). In the *left panels*, sector one is formed from the combination of positions in IC1 *(bright blue)*, IC2 *(light blue)* and IC3 *(cyan)*. Sector2 (IC4) is shown in red spheres, and forms a phyically contiguous unit structurally distinct from sector one.

```
In [16]: i = Image(filename = 'figs/Gprot_secstruct.png'); i
```

```
Out[16]:
```

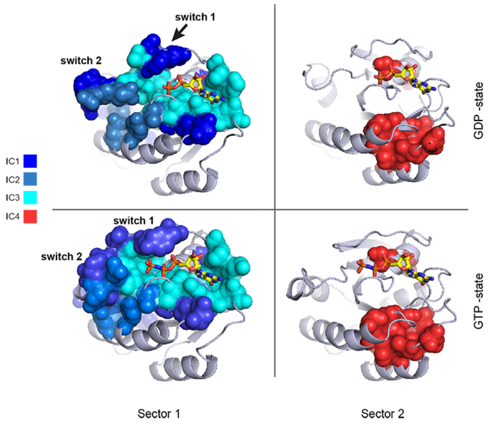

Print the sector positions, in a format suitable for pyMol, and create a pyMol session (in the Outputs directory) with the sectors (and decomposition into independent components) as seperate objects.

```
In [17]: for i,k in enumerate(sectors):
              sort_ipos = sorted(k.items)
              ats_ipos = ([Dseq['ats'][s] for s in sort_ipos])
              ic_pymol = ('+'.join(ats_ipos))
              print('Sector %i is composed of %i positions:' % (i+1,len(ats_ipos)))
              print(ic_pymol + "\n")
         sca.writePymol('5P21', sectors, Dsect['ics'], Dseq['ats'], \
                        'Outputs/PF00071.pml','A', '../Inputs/', 0)
```

```
Sector 1 is composed of 42 positions:
5+10+11+14+15+16+22+28+32+34+35+36+39+54+56+57+58+59+60+61+62+63+64+68+71+72+73+75+81+83+85+96+99+
110+116+117+119+144+145+146+147+156

Sector 2 is composed of 11 positions:
17+23+82+90+115+123+125+130+134+141+143
```

0.1.5 V. Sequence subfamilies and the basis of sector hierarchy.

How does the phylogenetic and functional heterogeneity in the MSA influence the sector definitions? To address this, we take advantage of mathematical methods for mapping between the space of positional and sequence correlations, as described in *Rivoire et al* (see equations 8-11). Using this mapping, we plot the top k^* ICs of the matrix $\tilde{C_{ij}}$ as 2-D scatter plots *(top row)*, and compare them to the corresponding sequence space divergence *(middle and bottom rows)*. The amino acid positions contributing to each IC are colored by sector *(sector 1 = blue, sector 2 = red, top row)*. The sequences are color-coded according to phylogenetic classifications *(middle row)* or G-protein class *(bottom row)* as we defined above.

```
In [18]: plt.rcParams['figure.figsize'] = 14, 10
         pairs= [[0,1],[1,2],[2,3]]
         for n,[k1,k2] in enumerate(pairs):
             plt.subplot(3,3,n+1)
             sca.figUnits(Dsect['Vpica'][:,k1], Dsect['Vpica'][:,k2], \
                          sectors, dotsize = 6)
             plt.xlabel('IC%i' % (k1+1), fontsize=16)
             plt.ylabel('IC%i' % (k2+1), fontsize=16)
             plt.subplot(3,3,n+4)
             sca.figUnits(Dsect['Upica'][:,k1], Dsect['Upica'][:,k2], \
                          phylo, dotsize = 6)
             plt.xlabel(r'$U^p_{%i}$' % (k1+1), fontsize=16)
             plt.ylabel(r'$U^p_{%i}$' % (k2+1), fontsize=16)
             plt.subplot(3,3,n+7)
             sca.figUnits(Dsect['Upica'][:,k1], Dsect['Upica'][:,k2], \
                          gprot_classes, dotsize = 6)
             plt.xlabel(r'$U^p_{%i}$' % (k1+1), fontsize=16)
             plt.ylabel(r'$U^p_{%i}$' % (k2+1), fontsize=16)
         plt.tight_layout()
```

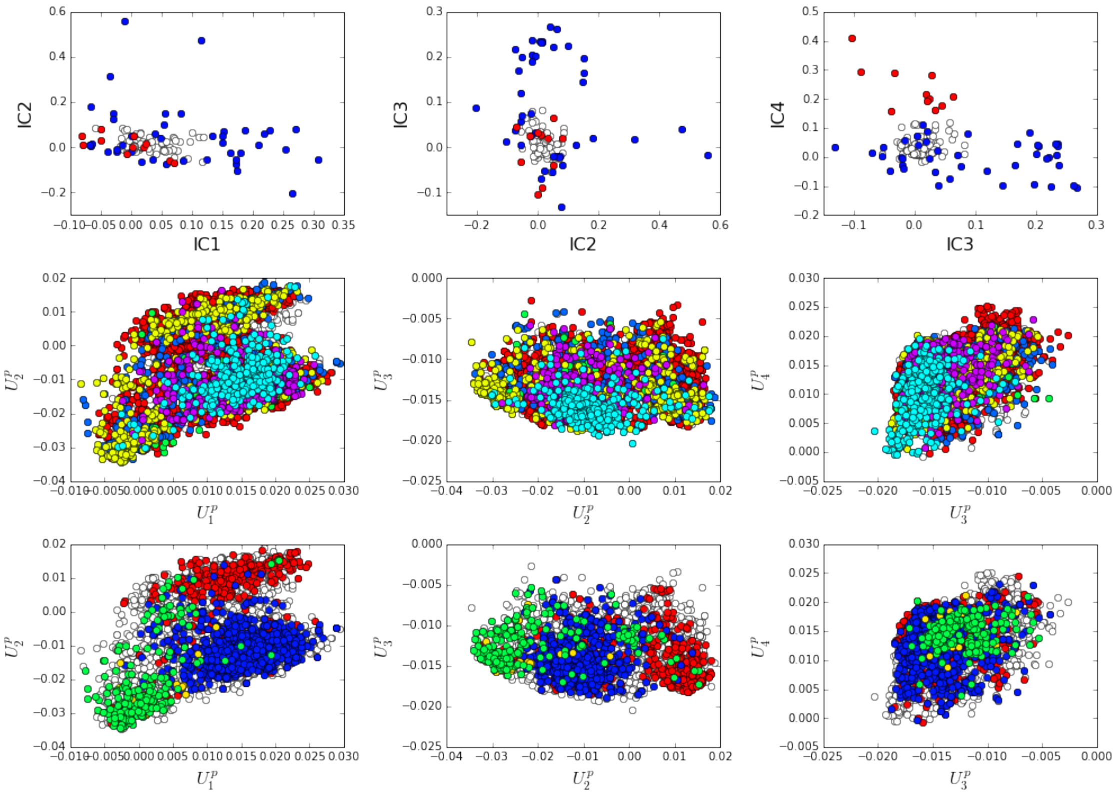

There is some clear divergence in G-protein subtype along U_1^p and U_2^p, indicating that the amino acid positions associated with IC1 and IC2 vary in a subtype-specific pattern. To more clearly see seperations in sequence classification, we also plot the above distributions of sequences (along U_1^p, U_2^p,U_3^p,and U_4^p) as stacked bar plots. This representation lets us directly see the contribution of sequences that might be hidden (due to overlapping points) on the above scatter plots. The *top row* reflects phylogenetic classifications and the *bottom row* shows G-protein functional classes.

In [19]:
```
plt.rcParams['figure.figsize'] = 15, 4

col = list()
for k in gprot_classes:
    col = col + [colorsys.hsv_to_rgb(k.col,1,1)]
for k in range(Dsect['kpos']):
    forhist = list()
    for group in gprot_classes:
        forhist.append([Dsect['Upica'][i,k] for i in group.items])
    plt.subplot(2,Dsect['kpos'],k+5)
    plt.hist(forhist, histtype='barstacked',color=col)
    plt.xlabel(r'$U^p_{%i}$' % (k+1), fontsize=16)

col = list()
for k in phylo:
    col = col + [colorsys.hsv_to_rgb(k.col,1,1)]
for k in range(Dsect['kpos']):
    forhist = list()
    for group in phylo:
```

```
        forhist.append([Dsect['Upica'][i,k] for i in group.items])
    plt.subplot(2,Dsect['kpos'],k+1)
    plt.hist(forhist, histtype='barstacked',color=col)
```

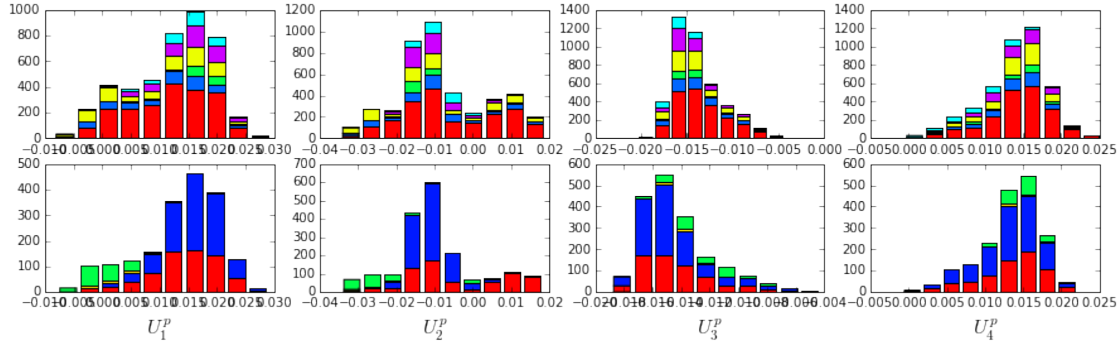

The interpretation for the two sectors is clear:

Sector 1 is composed of ICs 1,2 and 3 - we see above that the positions contributing to IC1 and IC2 seperate out the Ras-like *(red)* and Rho *(green)* g-protein functional classes (see the plots of U_1^p and U_2^p above). In contrast, the positions along IC3 and IC4 are associated with a homogeneous pattern of sequences; that is they have no obvious relationship to g-protein class or phylogeny. This suggests that sector 1 consists of a core element (IC3) that is conserved among G-proteins and two related/co-evolving parts which diverge in particular G-protein functional classes. The structural mapping of these positions is consistent with this interpretation - we observe that the positions associated with IC3 form the base of the nucleotide binding pocket (a general feature of the g-protein family) and that the IC1 and IC2 positions form a peripheral shell, which may reflect functional divergence in G-protein regulatory mechanisms in different family members.

Sector 2 is defined along (V_4^p). The sequences along the corresponding component (U_4^p) are homogeneously distributed with respect to both phylogeny and g-protein functional class, consistent with the notion that this sector is likley a global property of the entire alignment.

SCA_S1A

January 3, 2016

0.1 SCA6.0 - The S1A Serine Proteases

Summary: This script repeats the analysis of the S1A serine protease family, an example of a single protein containing at least three independent sectors. This analysis starts from the alignment used in: - Halabi, Rivoire, Leibler and Ranganathan. (2009) Cell 138:774-786. The goal is to examine the output of the pySCA (v6) analysis for the comparison to earlier results. In the S1A serine protease family, prior results indicate three sectors: "red" - associated with catalytic specificity, "green" - associated with catalytic function (the catalytic triad), and "blue" - associated with stability.

To facilitate comparison, here are the S1A sector definitions as described in Halabi et al. in a format suitable for pasting into pyMol:
Red: 17+161+172+176+177+180+183+187+188+189+191+192+213+215+216+220+221+226+227+228
Green: 19+33+42+43+55+56+57+58+102+141+142+184+194+195+196+197+198+199+213+214+216+225
Blue: 21+26+46+52+68+69+71+77+80+81+104+105+108+118+123+124+136+153+157+201+210+229+237+242+245

Prior to running this tutorial, the core calculation scripts must be executed from the command line as follows: >> ./scaProcessMSA.py Inputs/s1Ahalabi_1470_nosnakes.an -s 3TGI -c E -t -n >> ./scaCore.py Outputs/s1Ahalabi_1470_nosnakes.db >> ./scaSectorID.py Outputs/s1Ahalabi_1470_nosnakes.db Note that we supply pre-annotated alignments for all tutorial scripts *(the annotate_pfMSA step is slow, and should only be run once)*.

O.Rivoire, K.Reynolds and R.Ranganathan 10/2015

In [25]:
```
%matplotlib inline
from __future__ import division

import os
import time
import matplotlib.pyplot as plt
import numpy as np
import copy
import scipy.cluster.hierarchy as sch
from scipy.stats import scoreatpercentile
import matplotlib.image as mpimg
from IPython.display import display
from IPython.display import Image
from Bio.Seq import Seq
from Bio import motifs
import colorsys
import scaTools as sca
import mpld3
import cPickle as pickle
from optparse import OptionParser

if not os.path.exists('Outputs/'): os.makedirs('Outputs/')
```

To begin, we read in the results of the above three scripts (scaProcessMSA, scaCore and scaSectorID), stored as three dictionaries in the database s1Ahalabi_1470_nosnakes.db

In [26]:
```
db = pickle.load(open('Outputs/s1Ahalabi_1470_nosnakes.db','rb'))
Dseq = db['sequence']  #the results of scaProcessMSA
Dsca = db['sca']       #the results of scaCore
Dsect = db['sector']   #the results of scaSectorID
```

0.1.1 I. Alignment processing and composition

First, we print out a few statistics describing the alignment:

In [27]:
```
print("After processing, the alignment size is %i sequences and %i positions" % \
      (Dseq['Nseq'], Dseq['Npos']))
print("With sequence weights, there are %i effective sequences" % (Dseq['effseqs']))
```

```
After processing, the alignment size is 1344 sequences and 205 positions
With sequence weights, there are 928 effective sequences
```

To examine alignment composition, we plot a histogram of all pairwise sequence identities *(left panel)* and a global view of the sequence similarity matrix (defined by $S \equiv \frac{1}{L}XX^\top$) *(right panel)*. The data show that the alignment is described by a nearly homogeneous distribution of sequence identities with a mean value of about 30%.

In [28]:
```
# List all elements above the diagonal (i<j):
listS = [Dsca['simMat'][i,j] for i in range(Dsca['simMat'].shape[0]) \
         for j in range(i+1, Dsca['simMat'].shape[1])]
#Cluster the sequence similarity matrix
Z = sch.linkage(Dsca['simMat'],method = 'complete', metric = 'cityblock')
R = sch.dendrogram(Z,no_plot = True)
ind = map(int, R['ivl'])
#Plotting
plt.rcParams['figure.figsize'] = 9, 4
plt.subplot(121)
plt.hist(listS, Dseq['Npos']/2)
plt.xlabel('Pairwise sequence identities', fontsize=14)
plt.ylabel('Number', fontsize=14)
plt.subplot(122)
plt.imshow(Dsca['simMat'][np.ix_(ind,ind)], vmin=0, vmax=1); plt.colorbar();
```

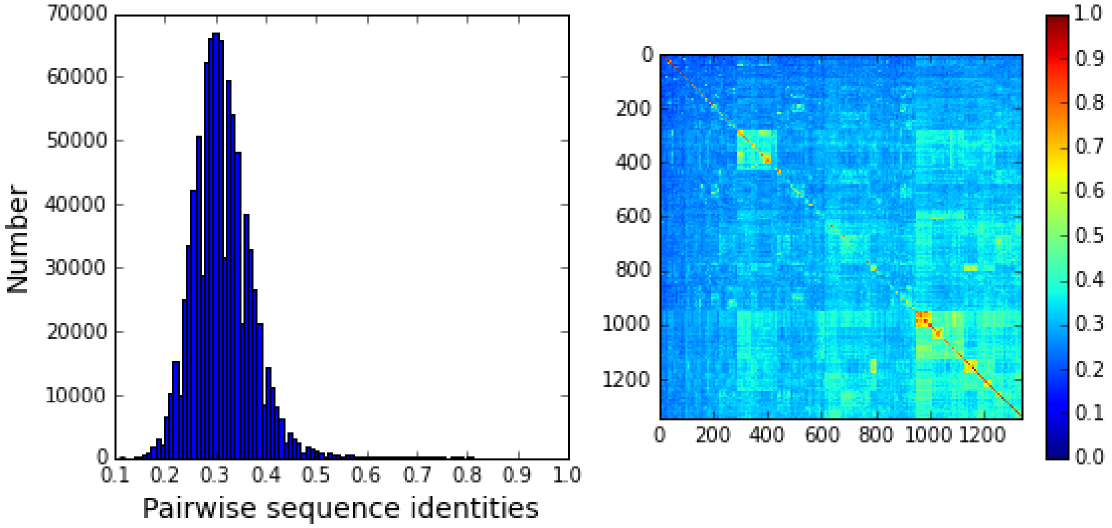

Sequence annotations: We construct a dictionary of phylogenetic annotations (parsed from the sequence headers) and check the representation of sequences in the top taxonomic levels. For each level, we print a list of taxonomic classes, and the number of sequences found for each. We also construct dictionaries of annotations for vertebrate vs. non vertebrate sequences, and substrate specificity (to later be used when mapping to sequence space). We print a list of common specificities following the list of taxonomic classes.

```
In [29]: #construct a dictionary of phylogenetic groups
         annot = dict()
         spec = dict()
         vert = dict()
         vert['vertebrate'] = []
         vert['not vertebrate']= []
         for i, h in enumerate(Dseq['hd']):
             s1 = h.split('__')
             s2 = s1[0].split('|')
             hs = s1[1].split('|')
             tax = []
             annot[s2[1]] = sca.Annot(s1[0], hs[2], ','.join(hs[3:-2]))
             if (hs[-2] in spec):
                 spec[hs[-2]].append(i)
             else:
                 spec[hs[-2]] = [i]
             if hs[0] == 'vertebrate':
                 vert[hs[0]].append(i)
             else:
                 vert['not vertebrate'].append(i)

         # Most frequent taxonomic groups:
         atleast = 10
         for level in range(3):
             descr_list = [a.taxo.split(',')[level] for a in annot.values() \
                           if len(a.taxo.split(',')) > level]
             descr_dict = {k:descr_list.count(k) for k in descr_list \
                           if descr_list.count(k)>=atleast}
             print '\n Level %i:' % level
             print descr_dict

         # Most frequent catalytic specificities:
         print '\nCatalytic Specificities: '
         for k in spec.keys():
             if len(spec[k]) > 5:
                 print k+': '+str(len(spec[k]))
```

```
Level 0:
{'Oligochaeta': 11, 'Malacostraca': 12, 'Actinopterygii': 127, 'Actinobacteria (class)': 14,
 'Mammalia': 447, 'Amphibia': 58, 'Insecta': 564, 'Arachnida': 25}

 Level 1:
{'Astigmata': 23, 'Decapoda': 12, 'Pleuronectiformes': 14, 'Carnivora': 45, 'Cypriniformes': 60,
 'Anura': 58, 'Hymenoptera': 15, 'Ruminantia': 61, 'Coleoptera': 81, 'Tetraodontiformes': 19,
 'Rodentia': 174, 'Perciformes': 12, 'Lepidoptera': 59, 'Actinomycetales': 14, 'Haplotaxida': 11,
 'Primates': 142, 'Diptera': 391, 'Laurasiatheria': 14}
```

```
 Level 2:
{'Streptomycetaceae': 12, 'Muridae': 170, 'Bovidae': 61, 'Lumbricidae': 11, 'Noctuidae': 28,
'Drosophilidae': 203, 'Tetraodontidae': 19, 'Tenebrionidae': 70, 'Pipidae': 58, 'Canidae': 44,
'Apidae': 10, 'Paralichthyidae': 12, 'Sarcoptidae': 14, 'Cyprinidae': 60, 'Suidae': 14,
'Culicidae': 153, 'Hominidae': 129, 'Cercopithecidae': 11}

Catalytic Specificities:
chymotrypsin: 85
kallikrein: 86
tryptase: 19
chymase: 8
allergen: 21
not trypsin: 34
thrombin: 7
plasminogen activator: 6
haptoglobin: 13
ELSE: 611
mast cell protease: 24
plasminogen: 6
marapsin: 6
granzyme: 52
elastase: 42
trypsin: 182
```

Based on this, we select both taxonomic groups and specificity classes, and associate them to colors for representation. We choose broad taxonomic groups (corresponding to Level 1), and well-populated specificity classes that are well-represented in the alignment . To see a complete legend that maps numeric codes to color, use:

```
>>> sca.figColors()
```

We start with taxonomic groups:

```
In [30]: phylo = list();
         fam_names = ['Oligochaeta', 'Malacostraca','Actinopterygii','Actinobacteria',\
                      'Mammalia','Amphibia','Insecta','Arachnida']
         col = (0,0.12,0.18,0.38,0.5,0.60,0.78,0.90)
         #Oligochaeta = red, Malacostraca = orange, Actinopterygii = yellow,
         #Actinobacteria = green, Mammalia = cyan, Amphibia = dark blue,
         #Insecta = purple, Arachnida = bright pink
         for i,k in enumerate(fam_names):
             sf = sca.Unit()
             sf.name = fam_names[i].lower()
             sf.col = col[i]
             sf.items = [j for j,q in enumerate(Dseq['hd'])  if sf.name in q.lower()]
             phylo.append(sf)
```

Now we assign substrate specificity classes, and also sort sequences into catalytically active and inactive enzymes (the haptoglobins).

```
In [31]: spec_names = ['chymotrypsin','trypsin','tryptase', 'kallikrein', 'granzyme']
         cat_act = ['active','haptoglobin']
         col_spec = [0,0.12,0.38,0.5,0.60,0.9]
         #chymotrypsin = red, trypsin = orange, tryptase = green,
         #kallikrein = cyan, granzyme = bright pink
```

```
col_vert = [0.12, 0.5]
#vertebrates = orange, invertebrates = cyan
col_act = [0.65,0.5]
#active = blue, inactive (haptoglobin) = cyan
spec_classes = []; cat_classes = []; vert_classes = [];

for i,k in enumerate(spec_names):
    sp = sca.Unit()
    sp.col = col_spec[i]
    sp.name = k
    sp.items = spec[k]
    spec_classes.append(sp)

for i,k in enumerate(cat_act):
    sp = sca.Unit()
    sp.col = col_act[i]
    sp.name = k
    if k == 'haptoglobin':
        sp.items = spec[k]
    else:
        sp.items = [k for k in range(Dseq['Nseq']) if (k not in spec['haptoglobin'] \
                                                        and k not in spec['ELSE'])]
    cat_classes.append(sp)

for i,k in enumerate(vert.keys()):
    sp = sca.Unit()
    sp.col = col_vert[i]
    sp.name = k
    sp.items = vert[k]
    vert_classes.append(sp)
```

To examine the relationship between **global** sequence similarity, phylogeny, and functional sub-class, we plot the top six independent components of the sequence correlation matrix (including sequence weights). In these plots, each point represents a particular sequence, and the distance between points reflects global sequence identity. The color codings are as follows: **top row:** phylogenetic annotation **second row:** active *(blue)* vs. inactive/haptoglobin *(cyan)* **third row:** specificity, chymotrypsin *(red)*, trypsin *(orange)*, tryptase *(green)*, kallikrein *(cyan)*, granzyme *(bright pink)* **fourth row:** vertebrate *(orange)* vs. invertebrate *(cyan)*

The data show some seperation of particular phylogenetic groups along ICs 1-5. For example, a subset of mammalian sequences *(cyan)* seperate out along IC2. In contrast, S1A specificity and catalytic activity are poorly seperated/classified using the independent components of the global sequence similarity matrix. This provides one indication that S1A enzymatic specificity and catalysis are not well-described by global sequence identity, but are largely encoded in a subset of positions. We will later see that these sequences *can* be seperated by projecting the sequence space using the positional correlations.

```
In [32]: plt.rcParams['figure.figsize'] = 9, 13
         U = Dsca['Uica'][1]
         pairs = [[2*i,2*i+1] for i in range(3)]
         for k,[k1,k2] in enumerate(pairs):
             plt.subplot(4,3,k+1)
             sca.figUnits(U[:,k1], U[:,k2], phylo)
             plt.xlabel(r"$IC_{seq}^{%i}$"%(k1+1), fontsize=16)
             plt.ylabel(r"$IC_{seq}^{%i}$"%(k2+1), fontsize=16)
             plt.subplot(4,3,k+4)
             sca.figUnits(U[:,k1], U[:,k2], cat_classes)
             plt.xlabel(r"$IC_{seq}^{%i}$"%(k1+1), fontsize=16)
```

```
    plt.ylabel(r"$IC_{seq}^{%i}$"%(k2+1), fontsize=16)
    plt.subplot(4,3,k+7)
    sca.figUnits(U[:,k1], U[:,k2], spec_classes)
    plt.xlabel(r"$IC_{seq}^{%i}$"%(k1+1), fontsize=16)
    plt.ylabel(r"$IC_{seq}^{%i}$"%(k2+1), fontsize=16)
    plt.subplot(4,3,k+10)
    sca.figUnits(U[:,k1], U[:,k2], vert_classes)
    plt.xlabel(r"$IC_{seq}^{%i}$"%(k1+1), fontsize=16)
    plt.ylabel(r"$IC_{seq}^{%i}$"%(k2+1), fontsize=16)
plt.tight_layout()
```

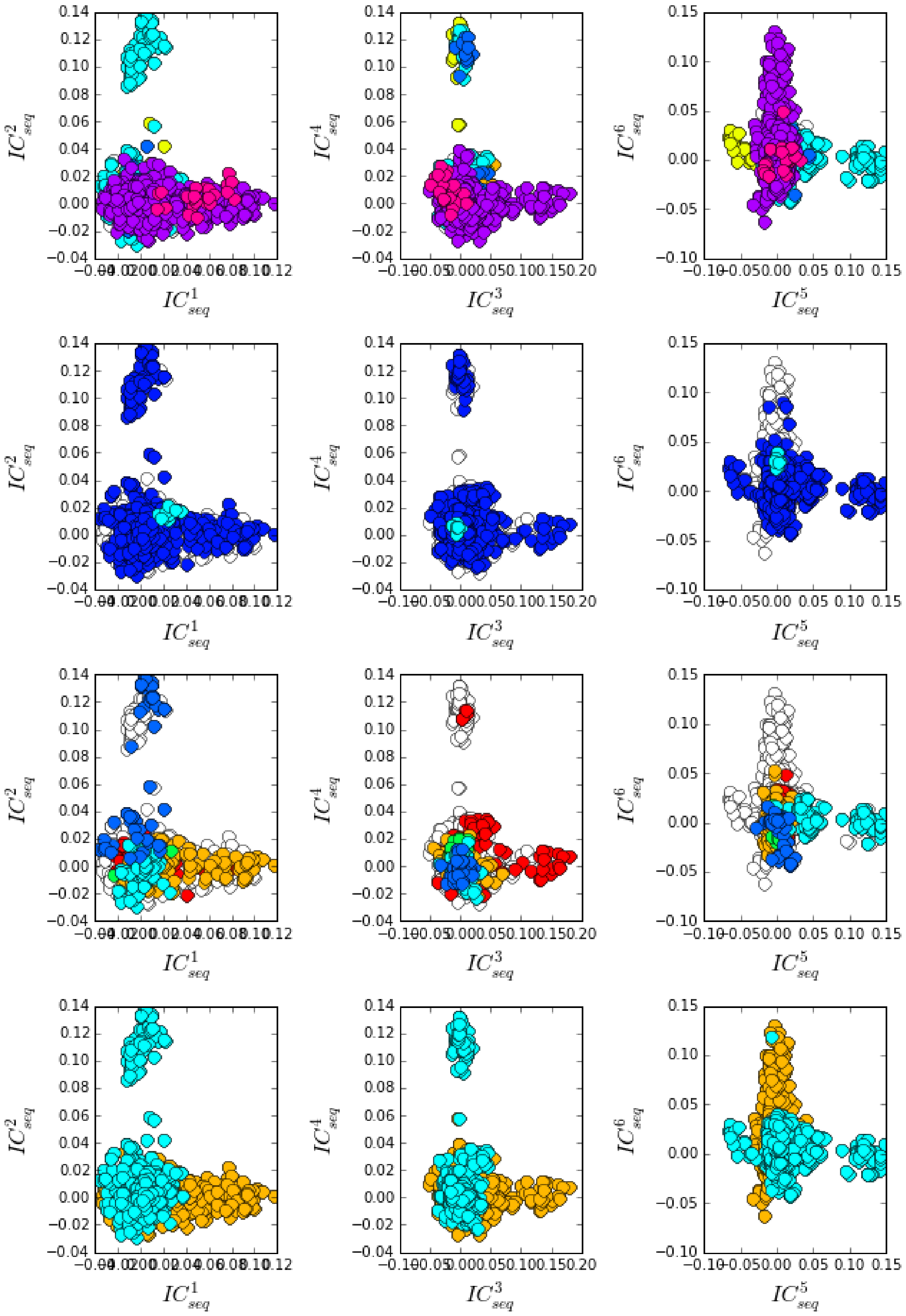
IC^2_seq
IC^1_seq
IC^4_seq
IC^3_seq
IC^6_seq
IC^5_seq

0.1.2 II. First-order statistics: position-specific conservation.

Plot the position-specific conservation values for each S1A protease position. D_i is calculated according to equation S4 (supplemental information).

In [33]:
```
fig, axs = plt.subplots(1,1, figsize=(9,4))
xvals = [i+1 for i in range(len(Dsca['Di']))]
xticks = [0,50,100,150,200]
plt.bar(xvals,Dsca['Di'], color='k')
plt.tick_params(labelsize=11); plt.grid()
axs.set_xticks(xticks);
labels = [Dseq['ats'][k] for k in xticks]
axs.set_xticklabels(labels);
plt.xlabel('Amino acid position', fontsize=18); plt.ylabel('Di', fontsize=18);
```

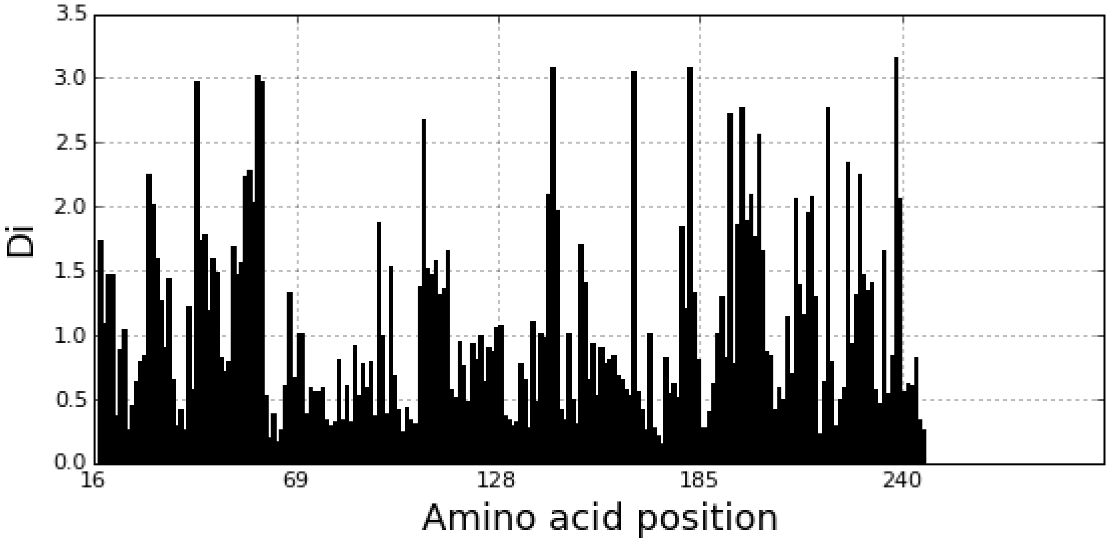

0.1.3 III. Second-order statistics: conserved correlations.

Plot the SCA correlation matrix ($\tilde{C_{ij}}$) computed according to Equations 4 + 5 of Rivoire et al.

In [34]:
```
plt.rcParams['figure.figsize'] = 9, 8
plt.imshow(Dsca['Csca'], vmin=0, vmax=1.4,interpolation='none',\
           aspect='equal')
```

Out[34]:
```
<matplotlib.image.AxesImage at 0x10a44ee90>
```

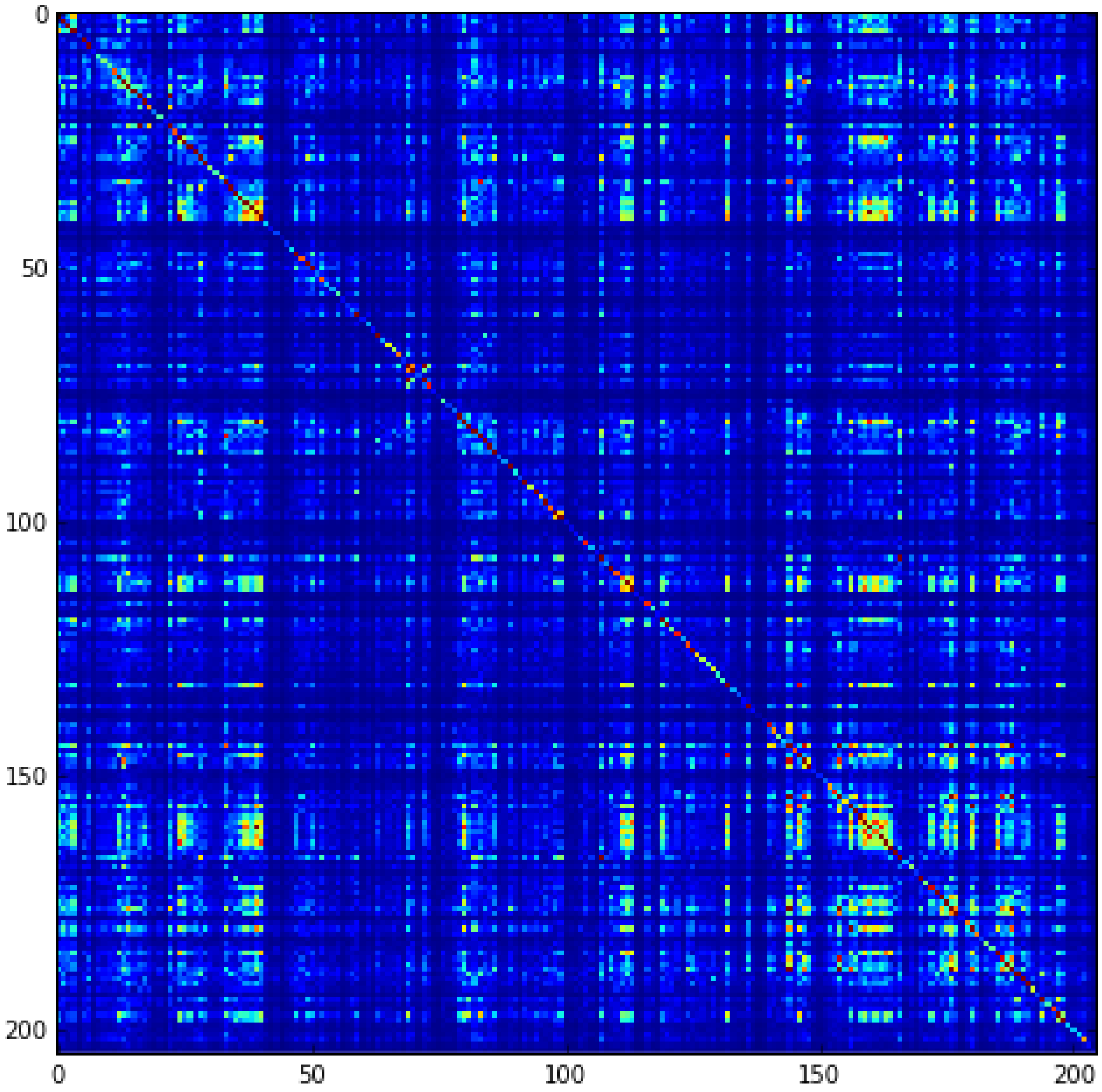

Plot the eigenspectrum of (1) the SCA positional coevolution matrix ($\tilde{C_{ij}}$) *(black bars)* and (2) 10 trials of matrix randomization for comparison. This graph is used to choose the number of significant eigenmodes ($k^* = 7$).

```
In [35]: plt.rcParams['figure.figsize'] = 9, 4
         hist0, bins = np.histogram(Dsca['Lrand'].flatten(), bins=Dseq['Npos'], \
                                    range=(0,Dsect['Lsca'].max()))
         hist1, bins = np.histogram(Dsect['Lsca'], bins=Dseq['Npos'], \
                                    range=(0,Dsect['Lsca'].max()))
         plt.bar(bins[:-1], hist1, np.diff(bins),color='k')
         plt.plot(bins[:-1], hist0/Dsca['Ntrials'], 'r', linewidth=3)
         plt.tick_params(labelsize=11)
         plt.xlabel('Eigenvalues', fontsize=18); plt.ylabel('Numbers', fontsize=18);
         print 'Number of eigenmodes to keep is %i' %(Dsect['kpos'])
         #mpld3.display()
```

```
Number of eigenmodes to keep is 7
```

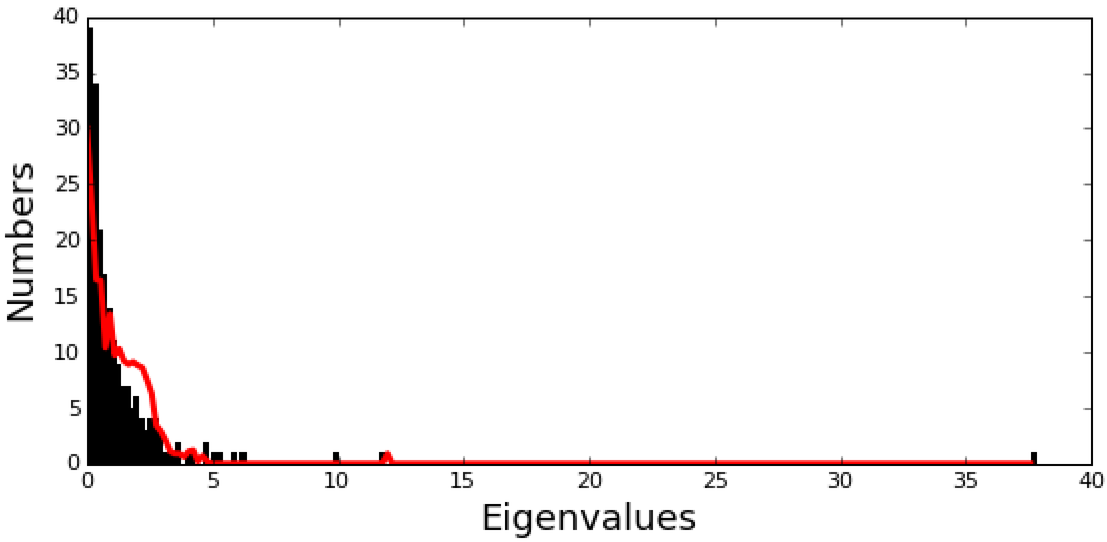

Plot the top significant eigenmodes *(top row)* and associated independent components *(bottom row)*. The ICs are an optimally independent representation of the seven different residue groups.

```
In [36]: plt.rcParams['figure.figsize'] = 9.5, 5.5
         pairs = [[0,1],[2,3],[4,5],[5,6]]
         EVs = Dsect['Vsca']
         ICs = Dsect['Vpica']
         for k,[k1,k2] in enumerate(pairs):
             plt.subplot(2,4,k+1)
             plt.plot(EVs[:,k1], EVs[:,k2], 'ok')
             plt.xlabel("EV%i"%(k1+1), fontsize=16)
             plt.ylabel("EV%i"%(k2+1), fontsize=16)
             plt.subplot(2,4,k+5)
             plt.plot(ICs[:,k1], ICs[:,k2], 'ok')
             plt.xlabel("IC%i"%(k1+1), fontsize=16)
             plt.ylabel("IC%i"%(k2+1), fontsize=16)
         plt.tight_layout()
```

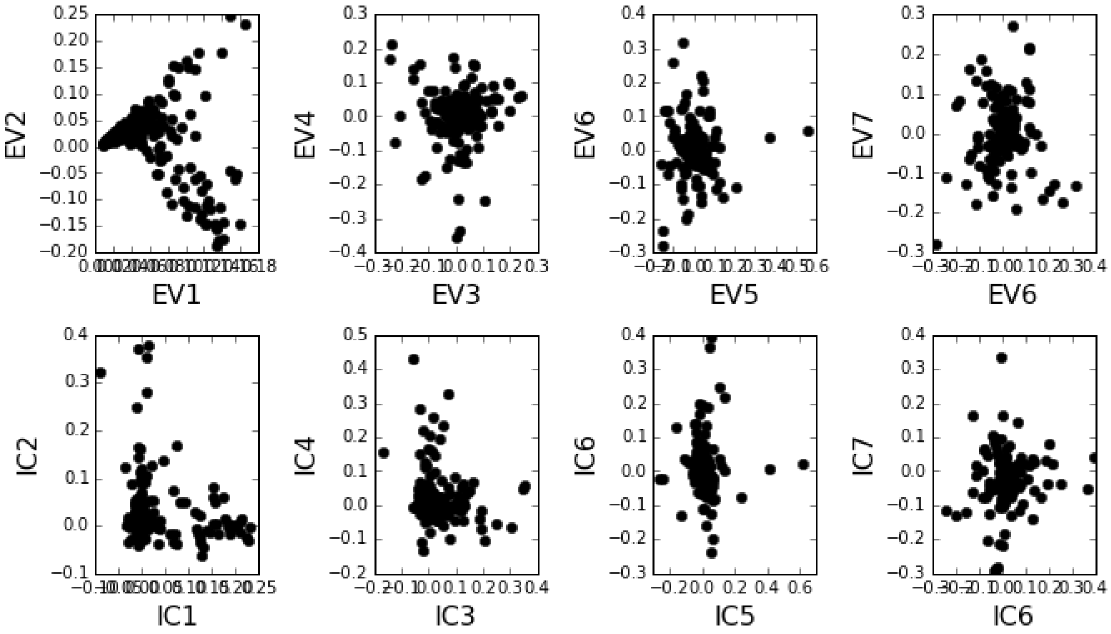

0.1.4 IV. Sector decomposition.

To define the positions with significant contributions to each of the independent components (ICs), we make a empirical fit for each IC to the t-distribution and select positions with greater than a specified cutoff on the CDF. We choose $p = 0.95$ as our cutoff. Note that since some positions might contribute significantly to more than one IC (an indication of non-independence of ICs), we apply a simple algorithm to assign such positions to one IC. Specifically, we assign positions to the IC with which it has the greatest degree of co-evolution.In the case of IC7, all of the positions are more strongly assigned to other ICs, so we end up with 6 ICs containing 97 total positions

The data indicate generally good fits for the top seven ICs, and we return the positions contributing to each IC in a format suitable for cut and paste into PyMol.

```
In [37]: plt.rcParams['figure.figsize'] = 8, 8

         Vpica = Dsect['Vpica']
         for k in range(Dsect['kpos']):
             iqr = scoreatpercentile(Vpica[:,k],75) - scoreatpercentile(Vpica[:,k],25)
             binwidth=2*iqr*(len(Vpica)**(-0.33))
             nbins=round((max(Vpica[:,k])-min(Vpica[:,k]))/binwidth)
             plt.subplot(Dsect['kpos'],1,k)
             h_params = plt.hist(Vpica[:,k], nbins)
             x_dist = np.linspace(min(h_params[1]), max(h_params[1]), num=100)
             plt.plot(x_dist,Dsect['scaled_pd'][k],'r',linewidth = 2)
             plt.plot([Dsect['cutoff'][k],Dsect['cutoff'][k]], [0,60], 'k--',linewidth = 1)
             plt.xlabel(r'$V^p_{%i}$'%(k+1), fontsize=14)
             plt.ylabel('Number', fontsize=14)

         for n,ipos in enumerate(Dsect['ics']):
             sort_ipos = sorted(ipos.items)
             ats_ipos = ([Dseq['ats'][s] for s in sort_ipos])
```

```
            ic_pymol = ('+'.join(ats_ipos))
            print('IC %i is composed of %i positions:' % (n+1,len(ats_ipos)))
            print(ic_pymol + "\n")
```

```
IC 1 is composed of 32 positions:
16+19+28+42+43+44+54+55+56+57+58+102+140+141+142+155+168+182+191+193+194+195+196+197+198+199+211+
214+220+225+237+238

IC 2 is composed of 22 positions:
29+138+160+161+172+176+177+180+183+184+188A+189+192+213+215+216+221+222+226+227+228+230

IC 3 is composed of 20 positions:
21+25+26+27+46+52+68+69+71+77+81+104+107+108+114+118+123+124+136+201

IC 4 is composed of 10 positions:
30+31+32+34+40+51+139+152+156+200

IC 5 is composed of 6 positions:
85+89+91+92+94+95

IC 6 is composed of 7 positions:
47+53+101+103+105+229+234

IC 7 is composed of 0 positions:

/Users/kreynolds/anaconda/lib/python2.7/site-packages/matplotlib/axes/_subplots.py:69:
MatplotlibDeprecationWarning: The use of 0 (which ends up being the _last_ sub-plot) is
deprecated in 1.4 and will raise an error in 1.5
  mplDeprecation)
```

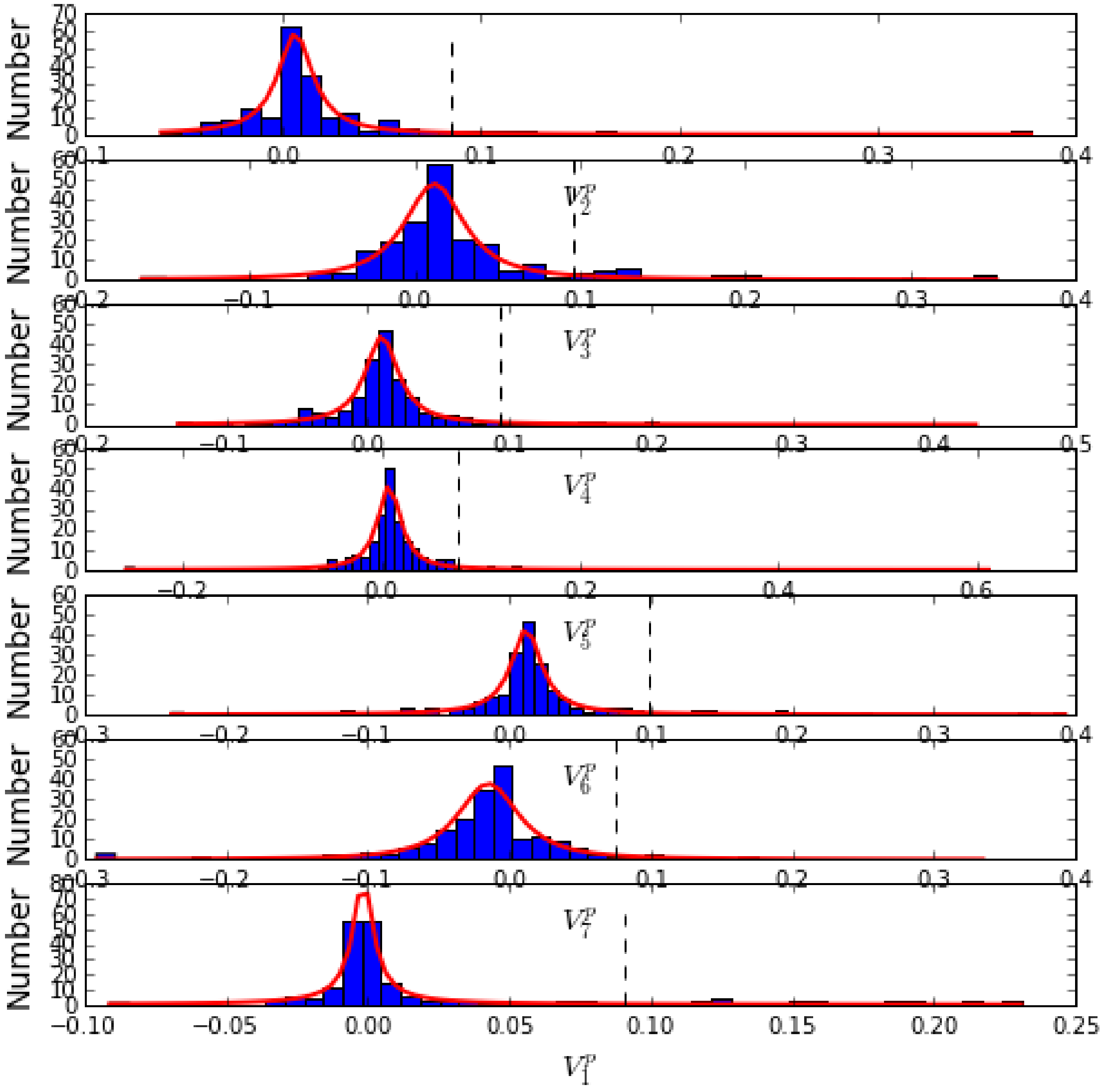

To define protein sectors, we examine the structure of the SCA positional correlation matrix with positions contributing to the top independent components (ICs) ordered by weight (*left panel*). This provides a basis to determine/interpret which ICs are truly statistically independent (defining an independent sector) and which represent hierarchical breakdowns of one sector. In this case, the data suggest that ICs 1-7 are indeed relatively independent.

```
In [38]: #plot the SCA positional correlation matrix, ordered by contribution to the top ICs
         plt.rcParams['figure.figsize'] = 10, 10
         plt.subplot(121)
         plt.imshow(Dsca['Csca'][np.ix_(Dsect['sortedpos'], Dsect['sortedpos'])], \
                     vmin=0, vmax=2,interpolation='none',\
                     aspect='equal',extent=[0,sum(Dsect['icsize']),\
                                            0,sum(Dsect['icsize'])])
         line_index=0
         for i in range(Dsect['kpos']):
             plt.plot([line_index+Dsect['icsize'][i],line_index+Dsect['icsize'][i]],\
```

```
              [0,sum(Dsect['icsize'])],'w', linewidth = 2)
    plt.plot([0,sum(Dsect['icsize'])],[sum(Dsect['icsize'])-\
            line_index,sum(Dsect['icsize'])-line_index],'w', linewidth = 2)
    line_index += Dsect['icsize'][i]

#define the new sector groupings - 3 total
sec_groups = ([0],[1],[2],[3],[4],[5])
sectors = list()

#defining color codes for the sectors
#s1 = green, s2 = red, s3 = blue, s4 = orange, s5 = hot pink, s6 = cyan
c = [0.4,0,0.7,0.15,0.9,0.5]
for n,k in enumerate(sec_groups):
    s = sca.Unit()
    all_items = list()
    all_Vp = list()
    for i in k:
        all_items = all_items+Dsect['ics'][i].items
        all_Vp = all_Vp+list(Dsect['ics'][i].vect)
    svals = np.argsort(all_Vp)
    s.items = [all_items[i] for i in svals]
    s.col = c[n]
    sectors.append(s)

#plot the re-ordered matrix
plt.subplot(122)
line_index=0
sortpos = list()
for s in sectors:
    sortpos.extend(s.items)
plt.imshow(Dsca['Csca'][np.ix_(sortpos, sortpos)], vmin=0, vmax=2.2,\
           interpolation='none',aspect='equal',\
           extent=[0,len(sortpos),0,len(sortpos)])
for s in sectors:
    plt.plot([line_index+len(s.items),line_index+len(s.items)],\
             [0,len(sortpos)],'w', linewidth = 2)
    plt.plot([0,sum(Dsect['icsize'])],[len(sortpos)-line_index,\
                    len(sortpos)-line_index],'w', linewidth = 2)
    line_index += len(s.items)
plt.tight_layout()
```

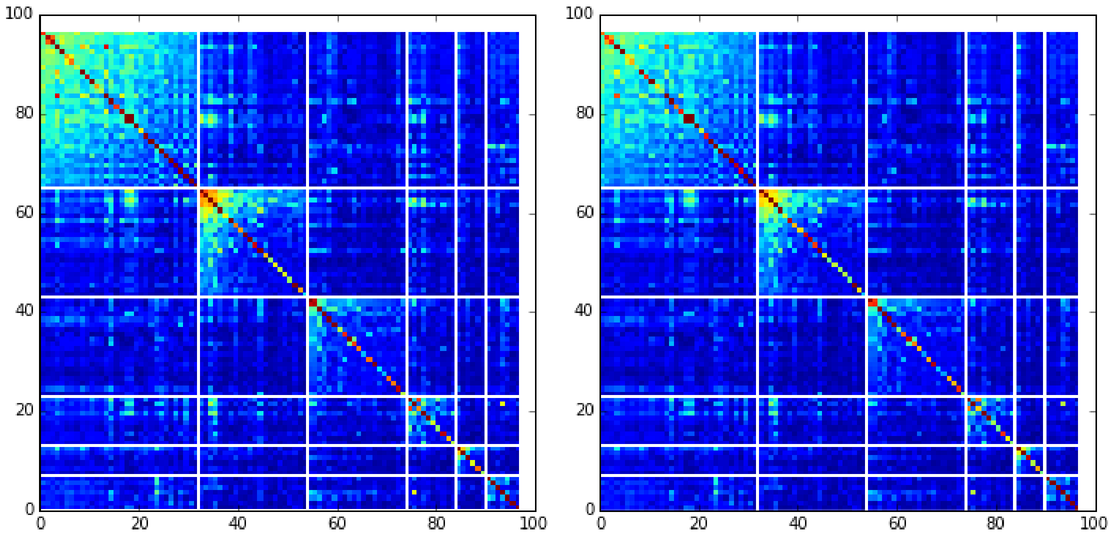

Print the sector positions, in a format suitable for pyMol, and create a pyMol session (in the Outputs directory) with the sectors (and decomposition into independent components) as seperate objects.

```
In [39]: for i,k in enumerate(sectors):
             sort_ipos = sorted(k.items)
             ats_ipos = ([Dseq['ats'][s] for s in sort_ipos])
             ic_pymol = ('+'.join(ats_ipos))
             print('Sector %i is composed of %i positions:' % (i+1,len(ats_ipos)))
             print(ic_pymol + "\n")
         sca.writePymol('3TGI', sectors, Dsect['ics'], Dseq['ats'], \
                        'Outputs/S1A.pml','E', '../Inputs/', 0)
```

```
Sector 1 is composed of 32 positions:
16+19+28+42+43+44+54+55+56+57+58+102+140+141+142+155+168+182+191+193+194+195+196+197+198+199+211+214+22[illegible]

Sector 2 is composed of 22 positions:
29+138+160+161+172+176+177+180+183+184+188A+189+192+213+215+216+221+222+226+227+228+230

Sector 3 is composed of 20 positions:
21+25+26+27+46+52+68+69+71+77+81+104+107+108+114+118+123+124+136+201

Sector 4 is composed of 10 positions:
30+31+32+34+40+51+139+152+156+200

Sector 5 is composed of 6 positions:
85+89+91+92+94+95

Sector 6 is composed of 7 positions:
47+53+101+103+105+229+234
```

These positions have clear physical consistency with the concept of sectors as functional, physically contiguous units in the protein structure. IC1 corresponds to the previously reported green sector, IC2 to the red sector, and IC3 is similar to the blue sector. ICs 4-6 follow the general principle of physical connectivity that seems to characterize sectors, but their functional meaning requires further study.

0.1.5 V. Sequence subfamilies and the basis of sector hierarchy.

How does the phylogenetic and functional heterogeneity in the MSA influence the sector definitions? To address this, we take advantage of mathematical methods for mapping between the space of positional and sequence correlations, as described in *Rivoire et al* (see equations 8-11). Using this mapping, we plot the top k^* ICs of the matrix $\tilde{C}_{ij}$ as 2-D scatter plots *(top row)*, and compare them to the corresponding sequence space divergence *(middle and bottom rows)*. The amino acid positions contributing to each IC are colored by sector *(sector 1 = green, sector 2 = red, sector 3 = blue, sector 4 = orange, sector 5 = pink, sector 6 = cyan, all top row)*. The sequences are color-coded according to phylogenetic classifications *(row 2)*, specificity *(row 3)*, vertebrate/invertebrate *(row 4)*, or catalytic activity *(row 5)* as we defined above.

```
In [40]: plt.rcParams['figure.figsize'] = 18, 20
         pairs= [[0,1],[1,2],[2,3],[4,5],[5,6]]
         for n,[k1,k2] in enumerate(pairs):
             plt.subplot(5,5,n+1)
             sca.figUnits(Dsect['Vpica'][:,k1], Dsect['Vpica'][:,k2], \
                          sectors, dotsize = 6)
             plt.xlabel('IC%i' % (k1+1), fontsize=16)
             plt.ylabel('IC%i' % (k2+1), fontsize=16)
             plt.subplot(5,5,n+6)
             sca.figUnits(Dsect['Upica'][:,k1], Dsect['Upica'][:,k2], \
                          phylo, dotsize = 6)
             plt.xlabel(r'$U^p_{%i}$' % (k1+1), fontsize=16)
             plt.ylabel(r'$U^p_{%i}$' % (k2+1), fontsize=16)
             plt.subplot(5,5,n+11)
             sca.figUnits(Dsect['Upica'][:,k1], Dsect['Upica'][:,k2], \
                          spec_classes, dotsize = 6)
             plt.xlabel(r'$U^p_{%i}$' % (k1+1), fontsize=16)
             plt.ylabel(r'$U^p_{%i}$' % (k2+1), fontsize=16)
             plt.subplot(5,5,n+16)
             sca.figUnits(Dsect['Upica'][:,k1], Dsect['Upica'][:,k2], \
                          vert_classes, dotsize = 6)
             plt.xlabel(r'$U^p_{%i}$' % (k1+1), fontsize=16)
             plt.ylabel(r'$U^p_{%i}$' % (k2+1), fontsize=16)
             plt.subplot(5,5,n+21)
             sca.figUnits(Dsect['Upica'][:,k1], Dsect['Upica'][:,k2], \
                          cat_classes, dotsize = 6)
             plt.xlabel(r'$U^p_{%i}$' % (k1+1), fontsize=16)
             plt.ylabel(r'$U^p_{%i}$' % (k2+1), fontsize=16)
         plt.tight_layout()
```

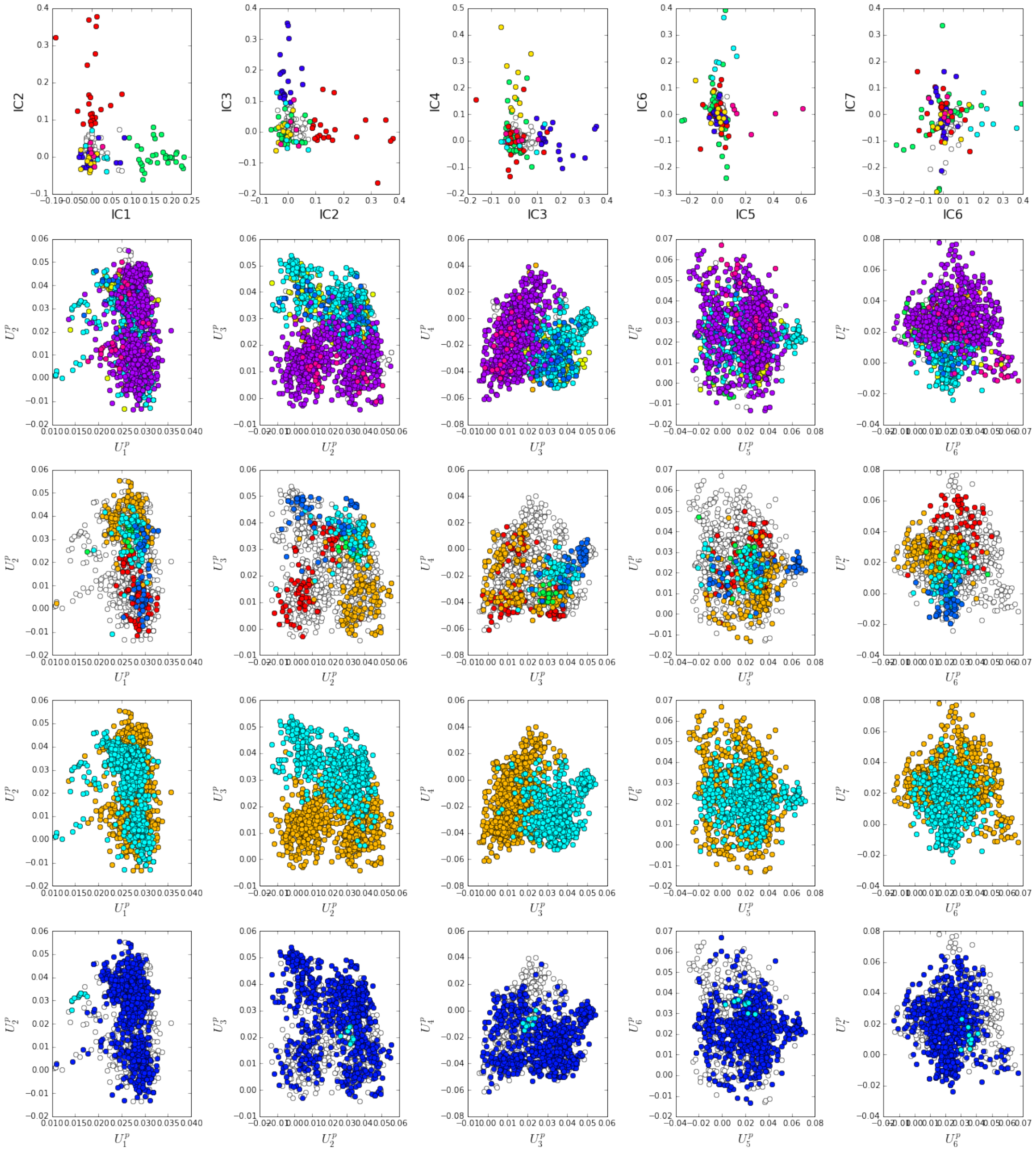

As previously described, we see a clear correpsondence between the top three sectors (red, green, blue) and distinct functional properties of the S1A protease family. IC1 *(the green sector)* seperates the non-catalytic haptoglobins *(cyan, bottom row)* from the catalytic family members. IC2 *(the red sector)* seperates the proteases by specificity, and IC3 *(the blue sector)* seperates vertebrate from invertebrate sequences. The remaining ICs (4-6) correspond to small numbers of physically contiguous positions, with no obvious relationship to particular sequence functional groups.

To more clearly see seperations in sequence classification, we also plot the above distributions of sequences as stacked bar plots.

In [41]:
```
plt.rcParams['figure.figsize'] = 18, 12

col = list()
```

```
for k in spec_classes:
    col = col + [colorsys.hsv_to_rgb(k.col,1,1)]
for k in range(Dsect['kpos']):
    forhist = list()
    for group in spec_classes:
        forhist.append([Dsect['Upica'][i,k] for i in group.items])
    plt.subplot(4,Dsect['kpos'],k+1)
    plt.hist(forhist, histtype='barstacked',color=col)

col = list()
for k in vert_classes:
    col = col + [colorsys.hsv_to_rgb(k.col,1,1)]
for k in range(Dsect['kpos']):
    forhist = list()
    for group in vert_classes:
        forhist.append([Dsect['Upica'][i,k] for i in group.items])
    plt.subplot(4,Dsect['kpos'],k+(Dsect['kpos'])+1)
    plt.hist(forhist, histtype='barstacked',color=col)

col = list()
for k in cat_classes:
    col = col + [colorsys.hsv_to_rgb(k.col,1,1)]
for k in range(Dsect['kpos']):
    forhist = list()
    for group in cat_classes:
        forhist.append([Dsect['Upica'][i,k] for i in group.items])
    plt.subplot(4,Dsect['kpos'],k+2*(Dsect['kpos'])+1)
    plt.hist(forhist, histtype='barstacked',color=col)


col = list()
for k in phylo:
    col = col + [colorsys.hsv_to_rgb(k.col,1,1)]
for k in range(Dsect['kpos']):
    forhist = list()
    for group in phylo:
        forhist.append([Dsect['Upica'][i,k] for i in group.items])
    plt.subplot(4,Dsect['kpos'],k+(3*Dsect['kpos'])+1)
    plt.hist(forhist, histtype='barstacked',color=col)
```

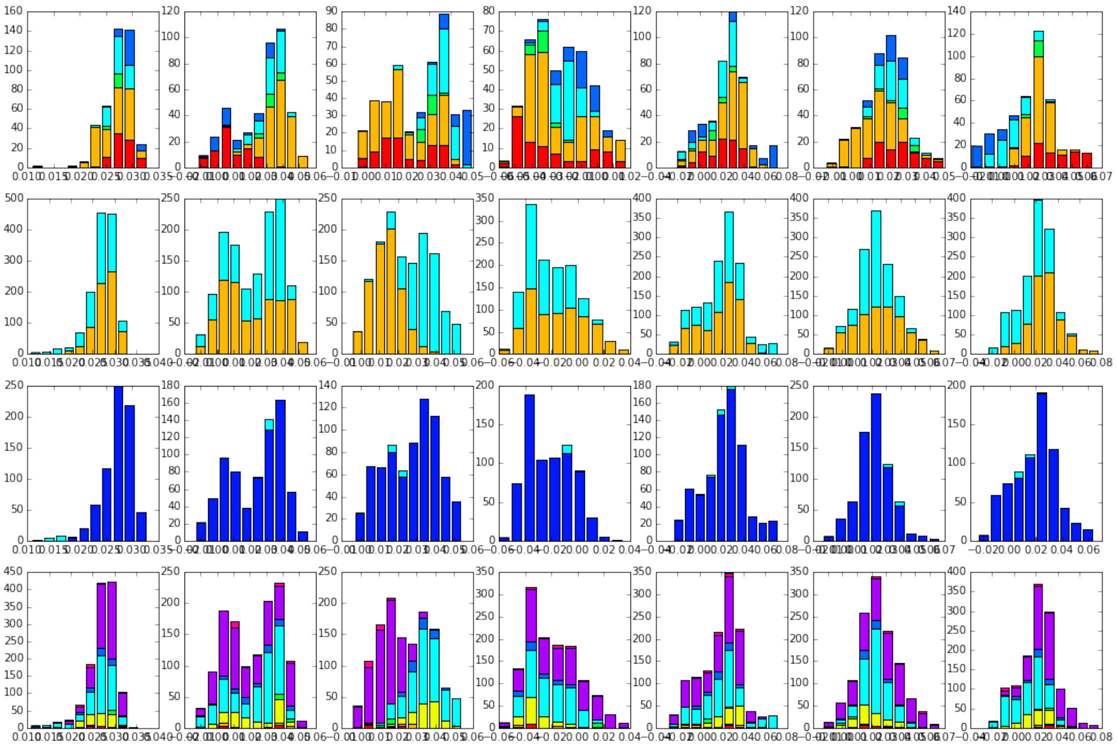

SCA_DHFR

January 3, 2016

0.1 SCA6.0 - The DHFR (dihydrofolate reductase) family

Summary This script describes the basic flow of the analytical steps in SCA6.0, using the DHFR family as an example. Here we compare results of the analysis for two different alignments: a PFAM alignment (PFAM PF00186) and an independent manually curated alignment constructed using a custom database of orthologous sequences (DHFR_PEPM3.an). Despite differences in the construction, sequence distribution and size of the two alignments, the sector definition is remarkably consistent: in both cases we arrive at a single sector assembled from six independent components.

For this tutorial, the core calculation scripts should be run from the command line as follows:

```
>> ./annotate_MSA.py Inputs/PF00186_full.txt -o Outputs/PF00186_full.an -a 'pfam'
>> ./scaProcessMSA.py Inputs/PF00186_full.an -s 1RX2 -c A -f 'Escherichia coli' -t -n
>> ./scaCore.py Outputs/PF00186_full.db
>> ./scaSectorID.py Outputs/PF00186_full.db
>> ./annotate_MSA.py Inputs/DHFR_PEPM3.fasta -o Outputs/DHFR_PEPM3.an -a 'ncbi'
-g Inputs/DHFR_PEPM3.gis
>> ./scaProcessMSA.py Inputs/DHFR_PEPM3.an -s 1RX2 -c A -t -n
>> ./scaCore.py Outputs/DHFR_PEPM3.db
>> ./scaSectorID.py Outputs/DHFR_PEPM3.db
```

Note that we supply annotated alignments for all tutorial scripts *(the annotate_pfMSA step is slow, and should only be run once)*.

O.Rivoire, K.Reynolds and R.Ranganathan 9/2014

```
In [15]: %matplotlib inline
         from __future__ import division

         import os
         import time
         import matplotlib.pyplot as plt
         import numpy as np
         import copy
         import colorsys
         import matplotlib.image as mpimg
         from IPython.display import display
         from IPython.display import Image
         import scipy.cluster.hierarchy as sch
         from scipy.stats import scoreatpercentile
         import scaTools as sca
         import mpld3
         import cPickle as pickle
         from optparse import OptionParser

         if not os.path.exists('Outputs/'): os.makedirs('Outputs/')
```

Read in the results of the above three scripts (scaProcessMSA, scaCore and scaSectorID), stored as dictionaries in the databases PF00186_full.db and DHFR_PEPM3.db. To see what variables are stored in each dictionary, use:

```
>> print dict.keys()
```

```
In [16]: Dseq = list(); Dsca = list(); Dsect = list()
         db = pickle.load(open('Outputs/PF00186_full.db','rb'))
         Dseq.append(db['sequence'])
         Dsca.append(db['sca'])
         Dsect.append(db['sector'])
         db2 = pickle.load(open('Outputs/DHFR_PEPM3.db', 'rb'))
         Dseq.append(db2['sequence'])
         Dsca.append(db2['sca'])
         Dsect.append(db2['sector'])
         N_alg = 2
         AlgName = ['PFAM', 'Manual']
```

0.1.1 I. Statistical Structure of the Multiple Sequence Alignment (MSA)

We start with a rough comparison of the sequence composition of the two alignments. Plot a histogram of all pairwise sequence identities *(left panel)* and a global view of the sequence similarity matrix (defined by $S \equiv \frac{1}{L}XX^\top$) *(right panel)*. The PFAM alignment is show in the *top row* and the manual alignment is shown in the *bottom row*. The manual alignment is smaller (644 seqs vs 2000 for PFAM), but both alignments are well-described by a nearly homogeneous distribution of sequence identities with a mean value of about 35%.

```
In [31]: ix = 1
         plt.rcParams['figure.figsize'] = 9, 15
         for k in range(N_alg):
             # List all elements above the diagonal (i<j):
             listS = [Dsca[k]['simMat'][i,j] for i in range(Dsca[k]['simMat'].shape[0]) \
                      for j in range(i+1, Dsca[k]['simMat'].shape[1])]
             #Cluster the sequence similarity matrix
             Z = sch.linkage(Dsca[k]['simMat'],method = 'complete', metric = 'cityblock')
             R = sch.dendrogram(Z,no_plot = True)
             ind = map(int, R['ivl'])
             #Plotting
             plt.rcParams['figure.figsize'] = 14, 4
             plt.subplot(2,2,ix)
             ix += 1
             plt.hist(listS, Dseq[k]['Npos']/2)
             plt.xlabel('Pairwise sequence identities', fontsize=14)
             plt.ylabel('Number', fontsize=14)
             plt.subplot(2,2,ix)
             ix += 1
             plt.imshow(Dsca[k]['simMat'][np.ix_(ind,ind)], vmin=0, vmax=1); plt.colorbar();
```

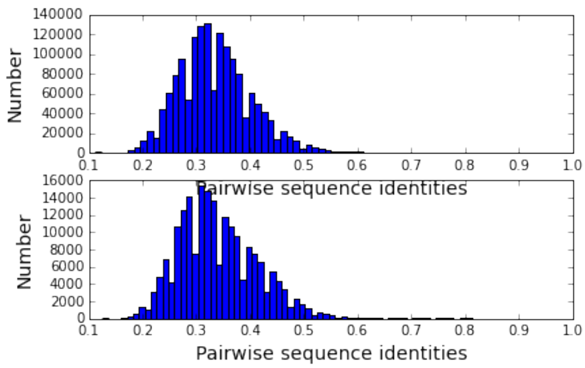

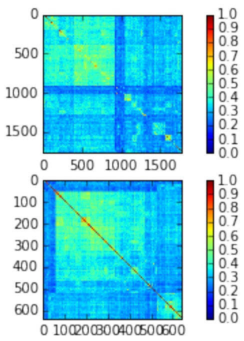

To examine: (1) sampling differences between the alignments and (2) the relationship between divergence in *sequence similarity* and *phylogeny* for both alignments, we plot the top independent components of the sequence correlation matrix (after sequence weighting), colored by phylogenetic group. We start by constructing a dictionary of phylogenetic annotations and checking the representation of sequences in the top taxonomic levels. The annotations are parsed from the sequence headers:

```
In [18]: for k in range(N_alg):
             print "Alignment: " + AlgName[k]
             #construct a dictionary of phylogenetic groups
             annot = dict()
             for i, h in enumerate(Dseq[k]['hd']):
                 hs = h.split('|')
                 if (len(hs) == 4):
                     annot[hs[0]] = sca.Annot(hs[1], hs[2], hs[3].replace('.',''))
                 elif (len(hs) == 3):
                     annot[hs[0]] = sca.Annot(hs[0],hs[1], hs[2].replace('.',''))

             # Most frequent taxonomic groups:
             atleast = 10
             for level in range(4):
                 descr_list = [a.taxo.split(',')[level] for a in annot.values() \
                               if len(a.taxo.split(',')) > level]
                 descr_dict = {k:descr_list.count(k) for k in descr_list \
                               if descr_list.count(k)>=atleast}
                 print '\n Level %i:' % level
                 print descr_dict
```

```
Alignment: PFAM

 Level 0:
{'Eukaryota': 212, 'Archaea': 21, 'Viruses': 38, 'Bacteria': 1494}

 Level 1:
{'environmental samples': 26, 'Tenericutes': 30, 'Alveolata': 11, 'Chlamydiae': 16,
 'dsDNA viruses': 37, 'Viridiplantae': 34, 'Firmicutes': 444, 'Bacteroidetes': 160, 'Fungi': 62,
 'Actinobacteria': 173, 'Metazoa': 82, 'Proteobacteria': 602, 'Euryarchaeota': 20}

 Level 2:
{'Mollicutes': 30, 'Sphingobacteriia': 13, 'Dikarya': 61, 'Negativicutes': 21, '
Deltaproteobacteria': 18, 'Betaproteobacteria': 119, 'Bacteroidia': 73,
```

```
'Coriobacteridae': 11, 'Flavobacteriia': 56, 'Chlamydiales': 16, 'Cytophagia': 12,
'Erysipelotrichi': 16, 'Bacillales': 94, 'Streptophyta': 26, ' no RNA stage': 37,
'Lactobacillales': 173, 'Chordata': 36, 'Clostridia': 140, 'Arthropoda': 31,
'Gammaproteobacteria': 312, 'Halobacteria': 18, 'Nematoda': 10, '
Actinobacteridae': 161, 'Alphaproteobacteria': 151}

 Level 3:
{'Erysipelotrichales': 16, 'Streptococcaceae': 66, 'Bacillaceae': 45, 'Rickettsiales': 12,
'Ascomycota': 51, 'Actinomycetales': 143, 'Mycoplasmataceae': 22,
'Pasteurellales': 20, 'Caudovirales': 14, 'Sphingobacteriales': 13, 'Staphylococcus': 14,
 'Coriobacteriales': 11, 'Sphingomonadales': 18, 'Lactobacillaceae': 66,
 'Clostridiales': 138, 'Bifidobacteriales': 18, 'Rhodobacterales': 27, 'Cytophagales': 12,
 'Xanthomonadales': 15, 'Burkholderiales': 69, 'Chromadorea': 10,
 'Paenibacillaceae': 17, 'Aeromonadales': 10, 'Oceanospirillales': 15,
 'Neisseriales': 27, 'Basidiomycota': 10, 'Halobacteriales': 18, 'Chlamydiaceae': 16,
 'Chromatiales': 20, 'Alteromonadales': 39, 'Rhizobiales': 76, 'Flavobacteriales': 53,
  'Enterococcaceae': 19, 'Legionellales': 10, 'Vibrionales': 39, 'Embryophyta': 26,
   'Enterobacteriales': 65, 'Selenomonadales': 21, 'Pseudomonadales': 43,
   'Craniata': 32, 'Hexapoda': 26, 'Bacteroidales': 73}
Alignment: Manual

 Level 0:
{'cellular organisms': 612}

 Level 1:
{' Eukaryota': 151, ' Bacteria': 461}

 Level 2:
{' Alveolata': 11, ' Opisthokonta': 137, ' Firmicutes': 100, ' Actinobacteria': 42,
 ' Bacteroidetes/Chlorobi group': 42, ' Proteobacteria': 259}

 Level 3:
{' Bacilli': 78, ' Gammaproteobacteria': 126, ' Betaproteobacteria': 58, ' Apicomplexa': 11,
 ' Bacteroidetes': 42, ' Fungi': 74, ' Actinobacteria': 42, ' Alphaproteobacteria': 69,
 ' Clostridia': 21, ' Metazoa': 62}
```

Based on this, we select taxonomic groups and colors for representation. Here, we just start by choosing the broadly well-represented groups. To see a complete color-coding legend, use:

```
>>> sca.figColors()
```

```
In [19]: phylo = list();
         fam_names = ['Eukaryota', 'Bacteroidetes', 'Firmicutes', \
                      'Actinobacteria', 'Proteobacteria']
         col = (0, 0.18, 0.38, 0.6, 0.8)
         #Eukaryota = red, Bacteriodetes = yellow, Firmicutes = green,
         #Actinobacteria = blue, Proteobacteria = purple
         for a in range(N_alg):
             phylo_alg = list()
             for i,k in enumerate(fam_names):
                 sf = sca.Unit()
                 sf.name = fam_names[i].lower()
                 sf.col = col[i]
                 sf.items = [j for j,q in enumerate(Dseq[a]['hd'])  \
                             if sf.name in q.lower()]
```

```
        phylo_alg.append(sf)
    phylo.append(phylo_alg)
```

Plot the top six independent components of the sequence correlation matrix (with sequence weights); color-coded by phylogenetic annotation. We compare the phylogenetic sampling for the PFAM alignment *(top row)* and manual alignment *(bottom row)*. The data show some very clear seqeunce distinctions based on phylogeny, and the two alignments seem to differ somewhat in the sequence divergence captured. In particular, the eukaryotic sequences *(in red)* seem to form a more distinct group in the manual alignment than in the PFAM alignment. For the PFAM alignment, the bacteriodetes *(yellow)* diverge along U_1, the actinobacteria *(blue)* along U_3, the firmicutes *(green)* along U_4 and U_5, and a subset of proteobacteria *(purple)* along U_6. For the manual alignment, the eukaryotes *(red)* diverge along U_2 and U_6, the actinobacteria *(blue)* along U_4, the firmicutes *(green)* along U_3, and a subset of proteobacteria *(purple)* along U_5

```
In [20]: plt.rcParams['figure.figsize'] = 9, 8
         ix = 1;
         for a in range(N_alg):
             U = Dsca[a]['Uica'][1]
             pairs = [[2*i,2*i+1] for i in range(3)]
             for k,[k1,k2] in enumerate(pairs):
                 plt.subplot(2,3,ix)
                 ix += 1
                 sca.figUnits(U[:,k1], U[:,k2], phylo[a])
                 #sca.figUnits(U[:,k1], U[:,k2], subfam)
                 plt.xlabel(r"${U'}^{(2)}_{%i}$"%(k1+1), fontsize=16)
                 plt.ylabel(r"${U'}^{(2)}_{%i}$"%(k2+1), fontsize=16)
             plt.tight_layout()
```

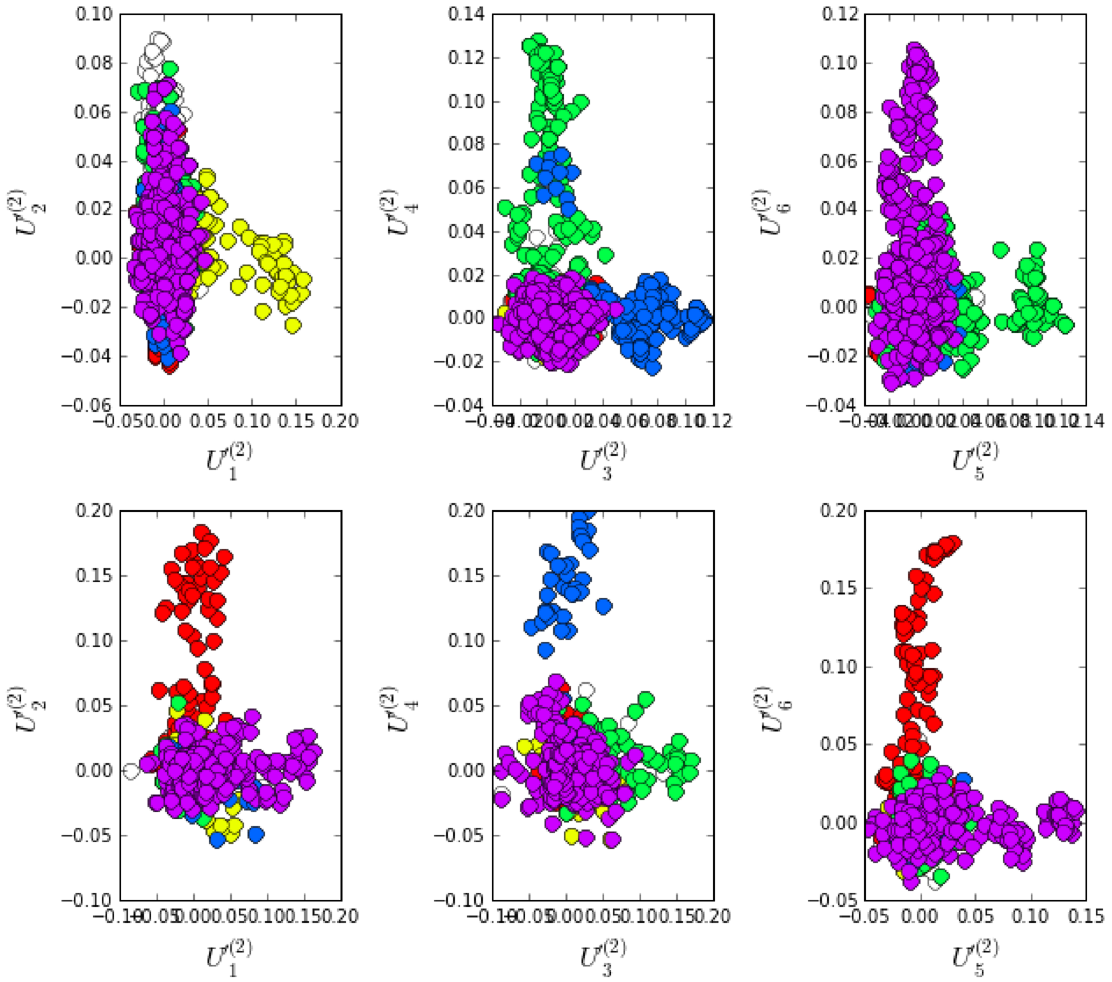

0.1.2 II. SCA. . . conservation and coevolution

Plot the eigenspectrum of (1) the SCA positional coevolution matrix ($\tilde{C_{ij}}$) *(black bars)* and (2) 10 trials of matrix randomization for comparison. This graph is used to choose the number of significant eigenmodes. Again, we plot the PFAM alignment in the *top row* and manual alignment in the *bottom row* for comparison. Overall the two eigenspectra are remarkably similar: due to small differences in the signficance cutoff, we define 6 signficant eigenmodes for the PFAM alignment, and 7 for the manual alignment.

```
In [21]: for a in range(N_alg):
             plt.rcParams['figure.figsize'] = 9, 4
             hist0, bins = np.histogram(Dsca[a]['Lrand'].flatten(), bins=Dseq[a]['Npos'], \
                                        range=(0,Dsect[a]['Lsca'].max()))
             hist1, bins = np.histogram(Dsect[a]['Lsca'], bins=Dseq[a]['Npos'],\
                                        range=(0,Dsect[a]['Lsca'].max()))
             plt.subplot(2,1,a+1)
             plt.bar(bins[:-1], hist1, np.diff(bins),color='k')
             plt.plot(bins[:-1], hist0/Dsca[a]['Ntrials'], 'r', linewidth=3)
             plt.tick_params(labelsize=11)
             plt.xlabel('Eigenvalues', fontsize=18); plt.ylabel('Numbers', fontsize=18);
```

```
            print 'Number of eigenmodes to keep is %i' %(Dsect[a]['kpos'])

        mpld3.display()
```

```
Number of eigenmodes to keep is 6
Number of eigenmodes to keep is 7
```

```
Out[21]: <IPython.core.display.HTML object>
```

To define the positions with significant contributions each of the independent components (ICs), we make a empirical fit for each IC to the t-distribution and select positions with greater than a specified cutoff on the CDF. We choose $p = 0.95$ as our cutoff. Note that since some positions might contribute significantly to more than one IC (and indication of non-independence of ICs), we apply a simple algorithm to assign such positions to one IC. Specifically, we assign positions to the IC with which it has the greatest degree of co-evolution.

For brevity, we don't plot the IC fits below (though we do in the other tutorial notebooks), but do print the list of positions associated with each IC for both alignments. Comparing between alignments, we can already see some distinctions in the residue positions associated to each component: IC1 is expanded for the manual alignment, ICs2,4+5 are similar for both alignments, and ICs 3+6 are swapped between the two alignments.

```
In [22]: plt.rcParams['figure.figsize'] = 20, 5
         for a in range(N_alg):
             print "alignment: "+AlgName[a]
             for n,ipos in enumerate(Dsect[a]['ics']):
                 sort_ipos = sorted(ipos.items)
                 ats_ipos = ([Dseq[a]['ats'][s] for s in sort_ipos])
                 ic_pymol = ('+'.join(ats_ipos))
                 print('IC %i is composed of %i positions:' % (n+1,len(ats_ipos)))
                 print(ic_pymol + "\n")
```

```
alignment: PFAM
IC 1 is composed of 11 positions:
3+5+6+11+18+45+92+100+111+125+153

IC 2 is composed of 19 positions:
7+14+15+27+31+35+42+43+44+46+49+54+57+94+95+96+113+122+126

IC 3 is composed of 3 positions:
21+22+24

IC 4 is composed of 5 positions:
23+28+32+51+55

IC 5 is composed of 13 positions:
9+13+25+37+39+56+64+71+81+105+107+121+133

IC 6 is composed of 7 positions:
40+47+50+52+53+59+61

alignment: Manual
IC 1 is composed of 13 positions:
13+23+25+27+28+30+32+37+38+51+55+63+121

IC 2 is composed of 18 positions:
```

```
7+14+15+22+31+35+42+43+44+46+49+54+57+61+94+95+96+113

IC 3 is composed of 9 positions:
40+47+50+52+53+59+81+100+103

IC 4 is composed of 12 positions:
3+6+11+18+45+60+90+92+111+125+147+149

IC 5 is composed of 12 positions:
9+39+56+64+71+85+104+105+107+115+133+158

IC 6 is composed of 5 positions:
21+24+93+122+123

IC 7 is composed of 4 positions:
5+126+151+156
```

To define protein sectors, we examine the structure of the SCA positional correlation matrix with positions contributing to the top independent components (ICs) ordered by weight *(left panel)*. Again we compare the results between the PFAM alignment *(top)* and manual alignment *(bottom)*. This provides a basis to determine/interpret which ICs are truly statistically independent (defining an independent sector) and which represent hierarchical breakdowns of one sector.

For both alignments, it seems that the ICs reflect a hierarchical break-down of a single sector, as determined by the high degree of co-evolution in the off-diagonal components (see the dendrogram that follows). In the *right panels* the ICs are combined and re-ordered by their contribution to V_1^p to better see this.

```
In [23]: sectors = list()
         ix = 1
         for a in range(N_alg):
             #plot the SCA positional correlation matrix, ordered by contribution
             #to the top ICs
             plt.rcParams['figure.figsize'] = 9, 9
             plt.subplot(2,2,ix); ix +=1;
             plt.imshow(Dsca[a]['Csca'][np.ix_(Dsect[a]['sortedpos'],\
                         Dsect[a]['sortedpos'])],vmin=0, vmax=2,\
                        interpolation='none',aspect='equal',\
                        extent=[0,sum(Dsect[a]['icsize']),0,\
                                sum(Dsect[a]['icsize'])])
             line_index=0
             for i in range(Dsect[a]['kpos']):
                 plt.plot([line_index+Dsect[a]['icsize'][i],\
                           line_index+Dsect[a]['icsize'][i]],\
                          [0,sum(Dsect[a]['icsize'])],'w', linewidth = 2)
                 plt.plot([0,sum(Dsect[a]['icsize'])],[sum(Dsect[a]['icsize'])\
                                -line_index,sum(Dsect[a]['icsize'])-line_index],\
                          'w', linewidth = 2)
                 line_index += Dsect[a]['icsize'][i]

             #combine all the ICs into a single sector and re-sort
             sec_groups = ([k for k in range(Dsect[a]['kpos'])])
             sectors_alg = list()
             s = sca.Unit()
             all_items = list()
             all_Vp = list()
             for i in range(Dsect[a]['kpos']):
```

```
    all_items = all_items+Dsect[a]['ics'][i].items
    tmp1 = Dsect[a]['Vpica'][Dsect[a]['ics'][i].items,:]
    all_Vp = all_Vp + list(tmp1[:,0].T)
svals = list(np.argsort(all_Vp)); svals.reverse()
s.items = [all_items[i] for i in svals]
s.col = (1/len(sec_groups))*n
sectors_alg.append(s)
sectors.append(sectors_alg)

#plot the re-ordered matrix
sortpos = list()
for s in sectors[a]:
    sortpos.extend(s.items)
plt.subplot(2,2,ix); ix += 1;
line_index=0
plt.imshow(Dsca[a]['Csca'][np.ix_(sortpos, sortpos)], \
           vmin=0, vmax=2,interpolation='none',aspect='equal',\
       extent=[0,len(sortpos),0,len(sortpos)])
for s in sectors[a]:
    plt.plot([line_index+len(s.items),line_index+len(s.items)],\
             [0,len(sortpos)],'w', linewidth = 2)
    plt.plot([0,sum(Dsect[a]['icsize'])],[len(sortpos)-line_index, \
                    len(sortpos)-line_index],'w', linewidth = 2)
    line_index += len(s.items)
plt.tight_layout()
```

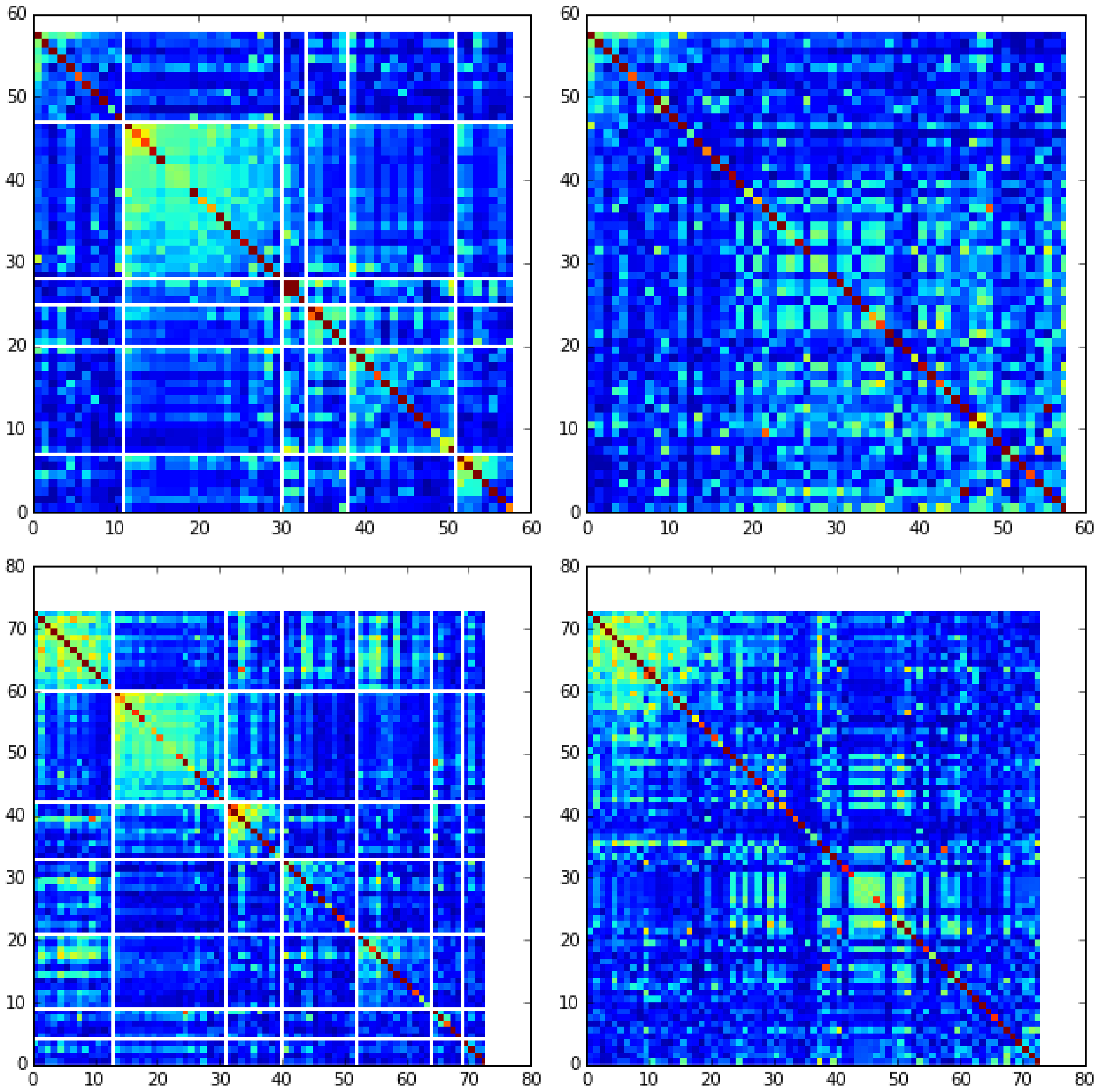

The below dendrogram diagrams the relationship between independent components for the PFAM alignment (the tree for the manual alignment is similar). In this plot, solid lines represent physically contiguous structural units, and dashed lines indicate spatially fragmented groups of residues.

```
In [24]: i = Image(filename='figs/DHFR_sec_hier.png'); i
```

Out[24]:

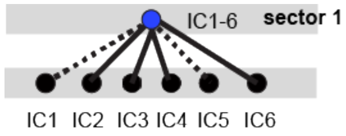

Print the sector positions (as defined for each alignment), in a format suitable for pyMol, and create two pyMol sessions with the sectors (and decomposition into independent components) as seperate objects.

```
In [25]: for a in range(N_alg):
             print "Alignment: " + AlgName[a]
             for i,k in enumerate(sectors[a]):
                 sort_ipos = sorted(k.items)
                 ats_ipos = ([Dseq[a]['ats'][s] for s in sort_ipos])
                 ic_pymol = ('+'.join(ats_ipos))
                 print('Sector %i is composed of %i positions:' % (i+1,len(ats_ipos)))
                 print(ic_pymol + "\n")

         sca.writePymol('1RX2', sectors[0], Dsect[0]['ics'], Dseq[0]['ats'],\
                        'Outputs/PF00186.pml','A', '../Inputs/', 0)
         sca.writePymol('1RX2', sectors[1], Dsect[1]['ics'], Dseq[1]['ats'],\
                        'Outputs/DHFR_PEPM3.pml','A', '../Inputs/', 0)
```

```
Alignment: PFAM
Sector 1 is composed of 58 positions:
3+5+6+7+9+11+13+14+15+18+21+22+23+24+25+27+28+31+32+35+37+39+40+42+43+44+45+46+47+49+50+51+52+53+54+
55+56+57+59+61+64+71+81+92+94+95+96+100+105+107+111+113+121+122+125+126+133+153

Alignment: Manual
Sector 1 is composed of 73 positions:
3+5+6+7+9+11+13+14+15+18+21+22+23+24+25+27+28+30+31+32+35+37+38+39+40+42+43+44+45+46+47+49+50+51+52+
53+54+55+56+57+59+60+61+63+64+71+81+85+90+92+93+94+95+96+100+103+104+105+107+111+113+115+121+122+123+
125+126+133+147+149+151+156+158
```

As is evident from the position lists above, and as shown below, the structural pattern of the two sectors and their associated decomposition into independent components is highly similar when compared between the two alignments. The main difference is that the sector (and independent components) for the manual alignment systematically includes a few more residue postions.

```
In [26]: i = Image(filename='figs/DHFR_decompv2.png'); i
```

Out[26]:

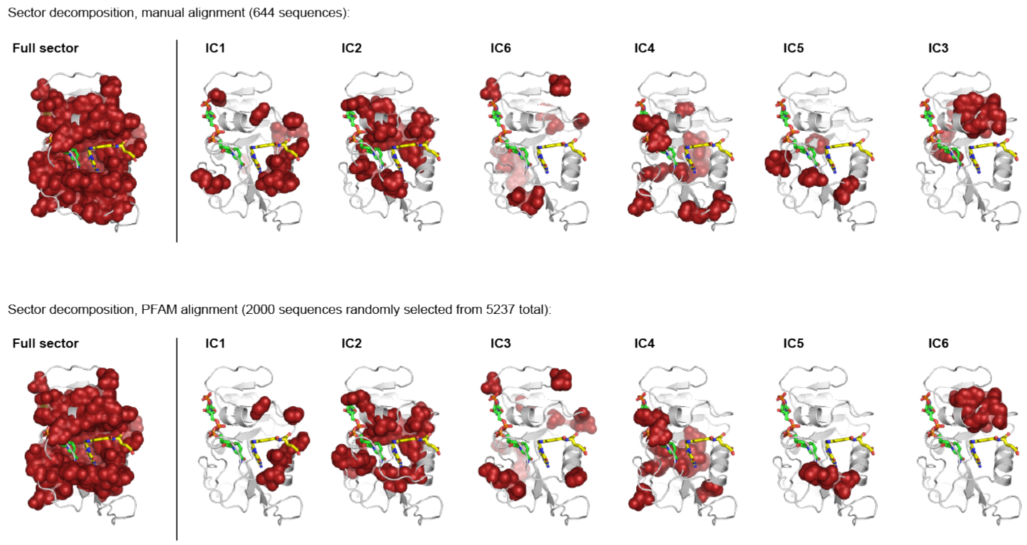

0.1.3 III. The phylogenetic basis of the sector hierarchy

How does the clear phylogenetic heterogeneity in the MSA influence the sector definitions? Since the sector definitions for the two alignments above are roughly equivalent, we only consider the larger (PFAM) alignment here. To address this, we take advantage of mathematical methods for mapping between the space of positional and sequence correlations, as described in *Rivoire et al.* Using this mapping, we plot the top k_{pos} ICs as 2-D scatter plots with the corresponding sequence space divergence.

```
In [27]: plt.rcParams['figure.figsize'] = 14, 10
         pairs= [[0,1],[2,3],[4,5]]
         for n,[k1,k2] in enumerate(pairs):
             plt.subplot(2,3,n+1)
             sca.figUnits(Dsect[0]['Vpica'][:,k1], Dsect[0]['Vpica'][:,k2],\
                          Dsect[0]['ics'], dotsize = 6)
             plt.xlabel(r'$V^p_{%i}$' % (k1+1), fontsize=16)
             plt.ylabel(r'$V^p_{%i}$' % (k2+1), fontsize=16)
             plt.subplot(2,3,n+4)
             sca.figUnits(Dsect[0]['Upica'][:,k1], Dsect[0]['Upica'][:,k2],\
                          phylo[0], dotsize = 6)
             plt.xlabel(r'$U^p_{%i}$' % (k1+1), fontsize=16)
             plt.ylabel(r'$U^p_{%i}$' % (k2+1), fontsize=16)
         plt.tight_layout()
```

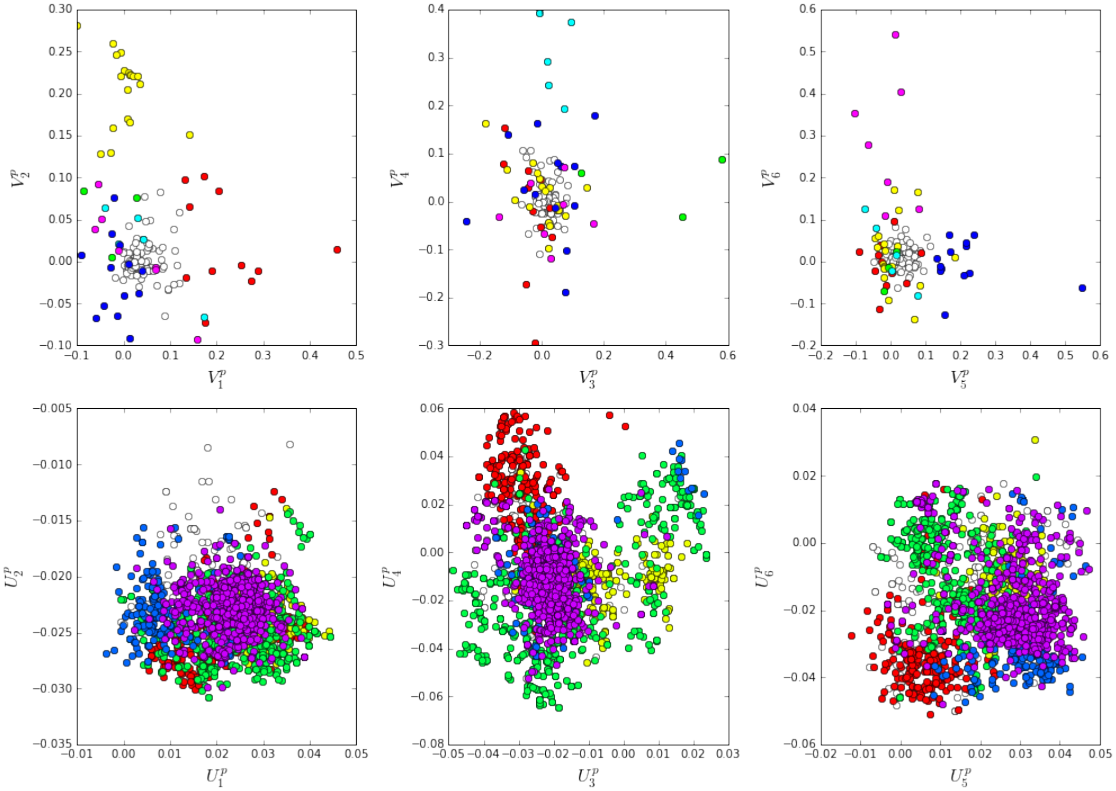


```
In [28]: plt.rcParams['figure.figsize'] = 20,8
         col = list()
         axis_lims = ([-0.06, 0.08],[-0.03, -0.01],[-0.05,0.03],[-0.01 ,0.05],\
```

```
                [-0.02 ,0.05],[-0.05 ,0.03]])
for k in phylo[0]:
    col = col + [colorsys.hsv_to_rgb(k.col,1,1)]
for k in range(Dsect[0]['kpos']):
    forhist = list()
    for group in phylo[0]:
        forhist.append([Dsect[0]['Upica'][i,k] for i in group.items])
    plt.subplot(2,Dsect[0]['kpos'],k+1)
    plt.hist(forhist, histtype='barstacked',color=col)
    plt.axis([axis_lims[k][0],axis_lims[k][1],0,600])
    plt.xlabel(r'$U^p_{%i}$' % (k+1), fontsize=16)
```

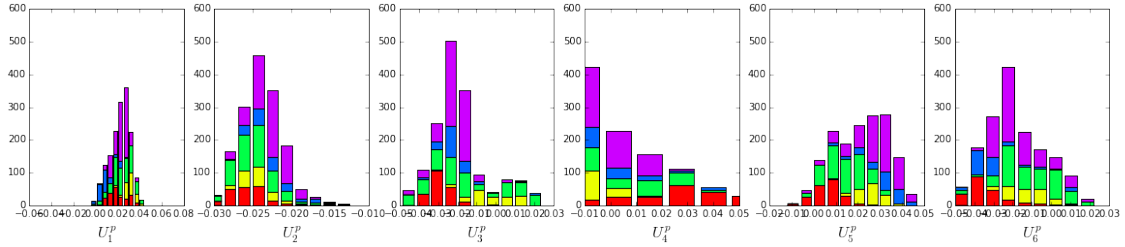

We see some association of phylogeny with sector positions at the phylum level: for example the positions along V^p_3 are associated with the divergence of some bacteriodetes *(yellow)* and firmicutes *(green)* along U^p_3. Further, the positions along ICs V^p_1,V^p_5 and V^p_6 seem to seperate the eukaryotes *(red)* from the prokaryotes.

So in conclusion, the DHFR family appears to have a single sector that can be decomposed into roughly six groups due to sequence divergence. Notably, the sector definition (and decomposition into ICs) is very similar for the two different sequence alignments.

SCA_betalactamase

January 3, 2016

0.1 SCA6.0 - The Beta-lactamase enzyme family

This script describes the basic flow of the analytical steps in SCA6.0, using the β-lactamase enzyme family as an example (PFAM PF13354). The alignment contains some subfamily structure (clades of related sequences) as evidenced in Section 1. We identify two sectors: a core sector surrounding the active site that is shared across all sequences, and a more peripheral sector containing groups of residues that diverge in particular subfamilies. For this tutorial, the core scripts should be run as follows:

```
>> ./annotate_pfMSA.py Inputs/PF13354_full.txt Inputs/PF13354_full.an
>> ./scaProcessMSA.py Inputs/PF13354_full.an -s 1FQG -c A -f 'Escherichia coli'-t -n
>> ./scaCore.py Outputs/PF13354_full.db
>> ./scaSectorID.py Outputs/PF13354_full.db
```

Note that we supply annotated alignments for all tutorial scripts *(the annotate_pfMSA step is slow, and should only be run once)*.

O.Rivoire, K.Reynolds and R.Ranganathan 9/2014

In [1]:
```
%matplotlib inline
from __future__ import division

import os
import time
import matplotlib.pyplot as plt
import numpy as np
import copy
import scipy.cluster.hierarchy as sch
from scipy.stats import scoreatpercentile
import scaTools as sca
import colorsys
import mpld3
import cPickle as pickle
from optparse import OptionParser

if not os.path.exists('Outputs/'): os.makedirs('Outputs/')
```

Read in the results of the above three scripts (scaProcessMSA, scaCore and scaSectorID), stored as three dictionaries in the database PF13354_full.db. To see what variables are stored in each dictionary, use:

```
>>> print dict.keys()
```

In [2]:
```
db = pickle.load(open('Outputs/PF13354_full.db','rb'))
Dseq = db['sequence']
Dsca = db['sca']
Dsect = db['sector']
```

0.1.1 I. Statistical Structure of the Multiple Sequence Alignment (MSA)

Plot a histogram of all pairwise sequence identities *(left panel)* and a global view of the sequence similarity matrix (defined by $S \equiv \frac{1}{L}XX^\top$) *(right panel)*. The data show that the alignment is described by a nearly bimodal distribution of sequence identities with peaks near 25% and 45%. From the matrix at right, it is clear that the alignment is composed of several distinct sequence families.

```
In [3]: # List all elements above the diagonal (i<j):
        listS = [Dsca['simMat'][i,j] for i in range(Dsca['simMat'].shape[0]) \
                 for j in range(i+1, Dsca['simMat'].shape[1])]
        #Cluster the sequence similarity matrix
        Z = sch.linkage(Dsca['simMat'],method = 'complete', metric = 'cityblock')
        R = sch.dendrogram(Z,no_plot = True)
        ind = map(int, R['ivl'])
        #Plotting
        plt.rcParams['figure.figsize'] = 9, 4
        plt.subplot(121)
        plt.hist(listS, Dseq['Npos']/2)
        plt.xlabel('Pairwise sequence identities', fontsize=14)
        plt.ylabel('Number', fontsize=14)
        plt.subplot(122)
        plt.imshow(Dsca['simMat'][np.ix_(ind,ind)], vmin=0, vmax=1); plt.colorbar();
```

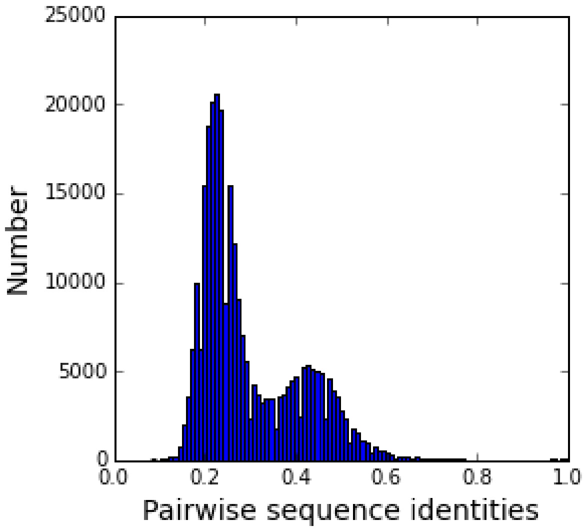

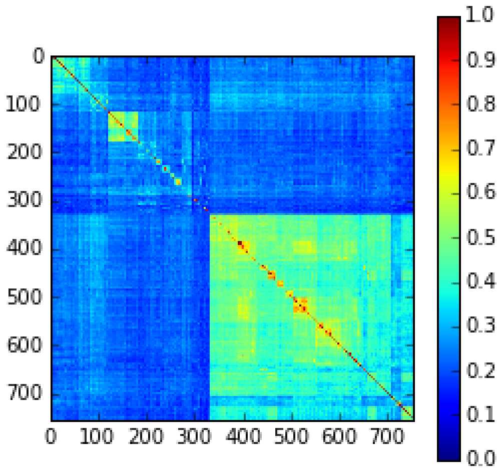

To examine the role of sequence and position weighting on the structure of the sequence space, we compute correlation matrices between all pairs of sequences, either with or without sequence and position weights and project the corresponding sequence space (by eigenvalue decomposition) down to a small set of top modes that contain the statistically dominant relationships between sequences. Since eigenvalue decomposition does not necessarily provide the best representation of sequence groups (for reasons described in "xx"), we also apply independent components analysis (or ICA) to the top few eigenmodes; this manipulation provides a representation in which the top groupings of sequences in the alignment (if such exists) should separate along the so-called independent components (or ICs). Below we plot the following eigenmodes *(top row)* and independent components *(bottom row)*:

- $U^{(0)}$ and $U'^{(0)}$, the top eigenmodes and ICs without any weights;
- $U^{(1)}$ and $U'^{(1)}$ the top eigenmodes and ICs with sequences weights;
- $U^{(2)}$ and $U'^{(2)}$ the top eigenmodes and ICs with both sequences and positional weights.

The sequences are colored by weight, with red indicating the most strongly downweighted sequences. In contrast to the g-protein example, we see that application of the sequence and position weights makes the sequence space apparently more uniform (removes some of the family or clade-like structure).

In [4]:
```
Useq = Dsca['Useq']
Uica = Dsca['Uica']
plt.rcParams['figure.figsize'] = 9, 8
ica = ["","","","’","’","’"]
for k,U in enumerate(Useq+Uica):
    plt.subplot(2,3,k+1)
    sca.figWeights(U[:,0], U[:,1], Dseq['seqw'][0])
    plt.xlabel(r'${U%s}^{(%i)}_1$'%(ica[k],k%3), fontsize=16)
    plt.ylabel(r'${U%s}^{(%i)}_2$'%(ica[k],k%3), fontsize=16)
plt.tight_layout()
```

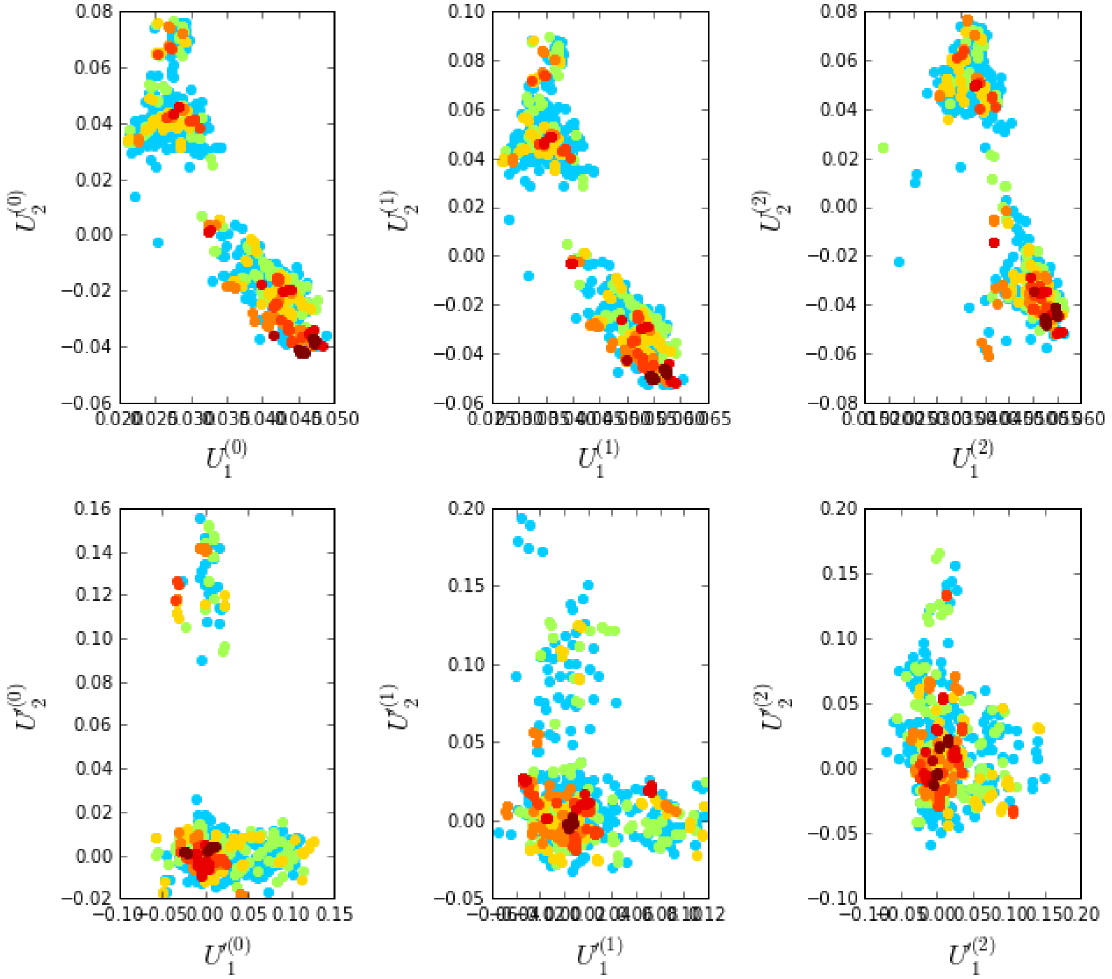

To examine the relationship between divergence in *sequence similarity* and *phylogeny* in the sequence-weighted alignment, we plot the top independent components of the sequence correlation matrix (after sequence weighting), colored by phylogenetic group. We start by constructing a dictionary of phylogenetic annotations and checking the representation of sequences in the top taxonomic levels. The annotations are parsed from the sequence headers.

```
In [5]: #construct a dictionary of phylogenetic groups
        annot = dict()
        for i, h in enumerate(Dseq['hd']):
            hs = h.split('|')
            annot[hs[0]] = sca.Annot(hs[1], hs[2], hs[3].replace('.',''))

        # Most frequent taxonomic groups:
        atleast = 10
        for level in range(4):
            descr_list = [a.taxo.split(',')[level] for a in annot.values() \
                          if len(a.taxo.split(',')) > level]
            descr_dict = {k:descr_list.count(k) for k in descr_list \
                          if descr_list.count(k)>=atleast}
            print '\n Level %i:' % level
            print descr_dict
```

```
Level 0:
{'Bacteria': 745}

 Level 1:
{'environmental samples': 18, 'Firmicutes': 100, 'Bacteroidetes': 49, 'Actinobacteria': 133,
'Cyanobacteria': 62, 'Proteobacteria': 353, 'Acidobacteria': 10}

 Level 2:
{'Lactobacillales': 11, 'Betaproteobacteria': 66, 'Bacteroidia': 25, 'Flavobacteriia': 11,
 'Gammaproteobacteria': 176, 'Chroococcales': 34, 'Oscillatoriales': 11,
 'Actinobacteridae': 128, 'Bacillales': 47, 'Clostridia': 33, 'Alphaproteobacteria': 103,
 'Nostocales': 11}

 Level 3:
{'Burkholderiales': 64, 'Flavobacteriales': 11, 'Sphingomonadales': 30, 'Rhizobiales': 39,
'Vibrionales': 24, 'Rhodospirillales': 15, 'Clostridiales': 28, 'Actinomycetales': 128,
'Thiotrichales': 13, 'Enterobacteriales': 79, 'Xanthomonadales': 17, 'Nostocaceae': 11,
'Bacteroidales': 25, 'Synechococcus': 14, 'Caulobacterales': 10, 'Bacillaceae': 29, '
Pseudomonadales': 25}
```

Based on this, we select taxonomic groups and colors for representation. Here, we just start by choosing the broadly well-represented groups. To see a complete color-coding legend, use:

```
>>> sca.figColors()
```

```
In [6]: phylo = list();
        fam_names = ['Firmicutes', 'Actinobacteria', 'Bacteroidetes', \
                     'Cyanobacteria', 'Proteobacteria']
        col = (0, 0.18, 0.38, 0.5, 0.6)
        #Firmicutes = red, Actinobacteria = yellow, Bacteroidetes = cyan,
        #Cyanobacteria = green, Proteobacteria = blue
        for i,k in enumerate(fam_names):
            sf = sca.Unit()
            sf.name = fam_names[i].lower()
            sf.col = col[i]
            sf.items = [j for j,q in enumerate(Dseq['hd'])  if sf.name in q.lower()]
            phylo.append(sf)
```

Plot the top six independent components of the sequence correlation matrix (with sequence weights); color-coded by phylogenetic annotation. The sequences clearly seperate into groups related by phylogeny;

the Proteobacteria *(blue)* seperate out on U_1, the Firmicutes *(red)* seperate out on U_2, the Cyanobacteria *(green)* seperate out on U_3, and the Bacteroidetes *(cyan)* seperate out on U_5.

In [16]:
```
plt.rcParams['figure.figsize'] = 9, 3.5
U = Dsca['Uica'][1]
pairs = [[2*i,2*i+1] for i in range(3)]
print pairs
for k,[k1,k2] in enumerate(pairs):
    plt.subplot(1,3,k+1)
    sca.figUnits(U[:,k1], U[:,k2], phylo)
    #sca.figUnits(U[:,k1], U[:,k2], subfam)
    plt.xlabel(r"${U'}^{(2)}_{%i}$"%(k1+1), fontsize=16)
    plt.ylabel(r"${U'}^{(2)}_{%i}$"%(k2+1), fontsize=16)
plt.tight_layout()
```

```
[[0, 1], [2, 3], [4, 5]]
```

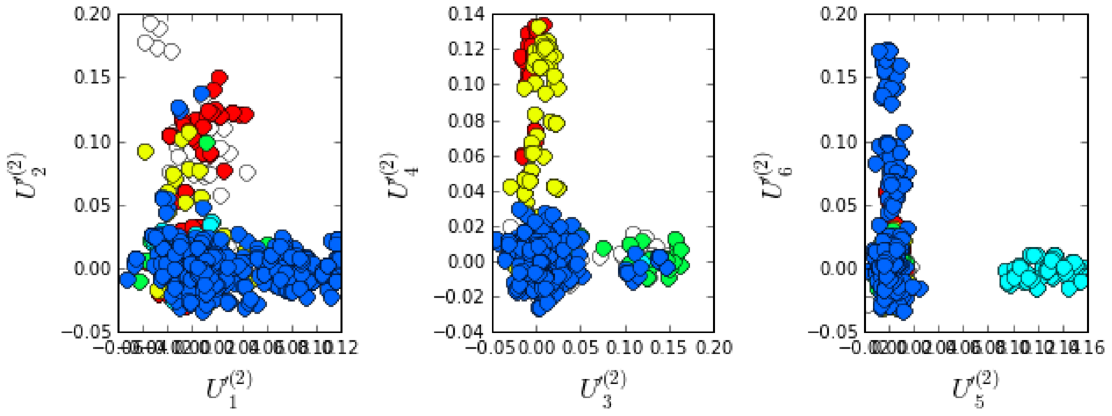

0.1.2 II. SCA conservation and coevolution

Plot the eigenspectrum of the SCA positional coevolution matrix ($\tilde{C_{ij}}$) *(black bars)* and 10 trials of matrix randomization for comparison *(red line)*. This graph is used to choose the number of significant eigenmodes.

In [17]:
```
plt.rcParams['figure.figsize'] = 9, 3.5
hist0, bins = np.histogram(Dsca['Lrand'].flatten(), bins=Dseq['Npos'], \
                           range=(0,Dsect['Lsca'].max()))
hist1, bins = np.histogram(Dsect['Lsca'], bins=Dseq['Npos'], \
                           range=(0,Dsect['Lsca'].max()))
plt.bar(bins[:-1], hist1, np.diff(bins),color='k')
plt.plot(bins[:-1], hist0/Dsca['Ntrials'], 'r', linewidth=3)
plt.tick_params(labelsize=11)
plt.xlabel('Eigenvalues', fontsize=18); plt.ylabel('Numbers', fontsize=18);
print 'Number of eigenmodes to keep is %i' %(Dsect['kpos'])
```

```
Number of eigenmodes to keep is 6
```

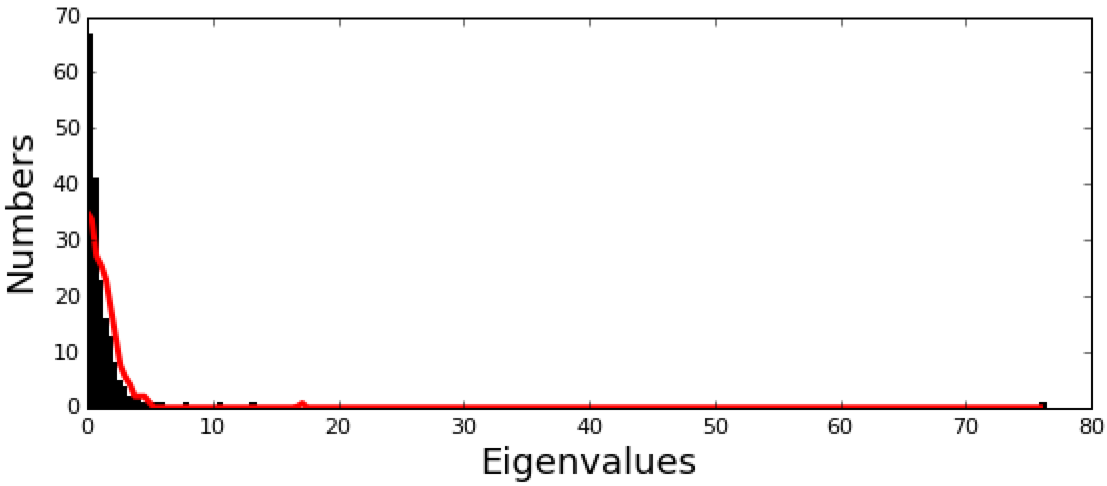

To define the positions with significant contributions each of the independent components (ICs), we make a empirical fit for each IC to the t-distribution and select positions with greater than a specified cutoff on the CDF. We choose $p = 0.95$ as our cutoff. Note that since some positions might contribute significantly to more than one IC (and indication of non-independence of ICs), we apply a simple algorithm to assign such positions to one IC. Specifically, we assign positions to the IC with which it has the greatest degree of co-evolution.

The data indicate generally good fits for the top six ICs, and we return the positions contributing to each IC in a format suitable for cut and paste into PyMol.

```
In [9]: plt.rcParams['figure.figsize'] = 10,5

        Vpica = Dsect['Vpica']
        for k in range(Dsect['kpos']):
            iqr = scoreatpercentile(Vpica[:,k],75) - scoreatpercentile(Vpica[:,k],25)
            binwidth=2*iqr*(len(Vpica)**(-0.33))
            nbins=round((max(Vpica[:,k])-min(Vpica[:,k]))/binwidth)
            plt.subplot(1,Dsect['kpos'],k)
            h_params = plt.hist(Vpica[:,k], nbins)
            x_dist = np.linspace(min(h_params[1]), max(h_params[1]), num=100)
            plt.plot(x_dist,Dsect['scaled_pd'][k],'r',linewidth = 2)
            plt.xlabel(r'$V^p_{%i}$'%(k+1), fontsize=14)
            plt.ylabel('Number', fontsize=14)

        for n,ipos in enumerate(Dsect['ics']):
            sort_ipos = sorted(ipos.items)
            ats_ipos = ([Dseq['ats'][s] for s in sort_ipos])
            ic_pymol = ('+'.join(ats_ipos))
            print('IC %i is composed of %i positions:' % (n+1,len(ats_ipos)))
            print(ic_pymol + "\n")
```

```
IC 1 is composed of 21 positions:
65+66+71+117+123+125+136+157+164+169+170+178+179+180+210+229+233+247+250+251+255

IC 2 is composed of 14 positions:
70+73+91+130+131+132+134+143+156+182+234+235+236+245
```

```
IC 3 is composed of 18 positions:
72+102+105+106+107+126+144+145+166+183+185+199+207+215+216+226+238+244

IC 4 is composed of 11 positions:
85+87+97+129+200+203+211+221+225+231+240

IC 5 is composed of 15 positions:
68+69+119+122+139+149+151+153+161+162+163+181+186+193+220

IC 6 is composed of 2 positions:
241+256

/Users/kreynolds/anaconda/lib/python2.7/site-packages/matplotlib/axes/_subplots.py:69:
 MatplotlibDeprecationWarning: The use of 0 (which ends up being the _last_ sub-plot)
 is deprecated in 1.4 and will raise an error in 1.5
  mplDeprecation)
```

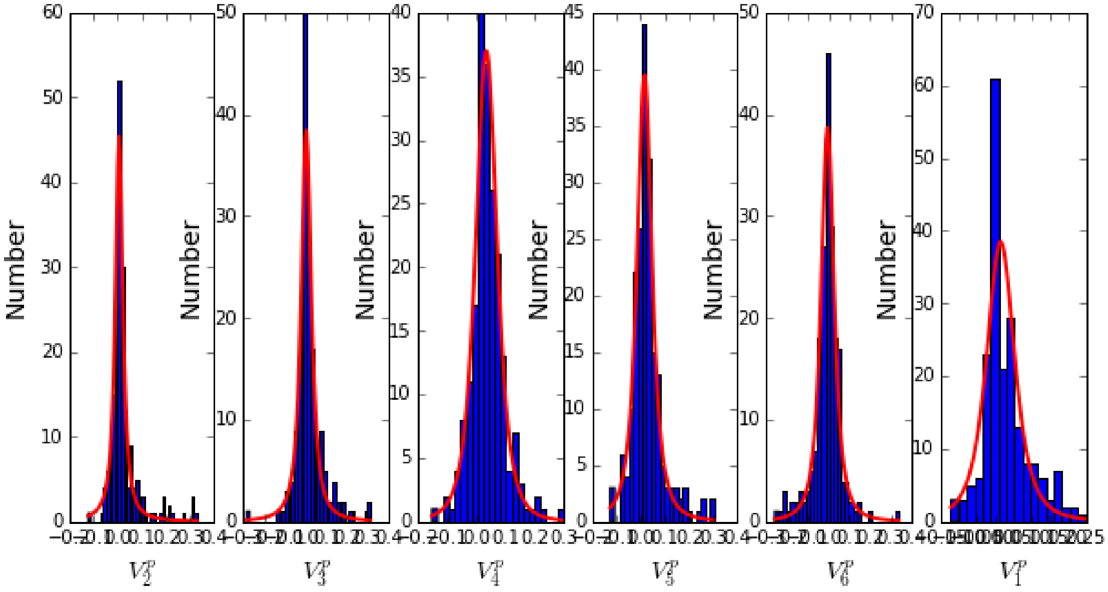

To define protein sectors, we examine the structure of the SCA positional correlation matrix with positions contributing to the top independent components (ICs) ordered by weight *(left panel)*. This provides a basis to determine/interpret which ICs are truly statistically independent (defining an independent sector) and which represent hierarchical breakdowns of one sector.

IC 2 appears more distinct and is considered an independent sector *(sector 1)*. ICs 1,3,5,and 6 are strongly co-evolving, and should be combined into one sector. IC 4 also appears to be related to [1,3,5,6] and the combination of 1,3,4,5,6 makes up sector two. The sectors (2 in total) are defined accordingly, and in the *right panel*, these independent components have been re-ordered accordingly to visualize this decomposition.

```
In [10]: #plot the SCA positional correlation matrix, ordered by contribution to the top ICs
         plt.rcParams['figure.figsize'] = 10, 10
         plt.subplot(121)
         plt.imshow(Dsca['Csca'][np.ix_(Dsect['sortedpos'], Dsect['sortedpos'])], \
                    vmin=0, vmax=2,interpolation='none',aspect='equal',\
                    extent=[0,sum(Dsect['icsize']),0,sum(Dsect['icsize'])])
         line_index=0
```

```
for i in range(Dsect['kpos']):
    plt.plot([line_index+Dsect['icsize'][i],line_index+Dsect['icsize'][i]],\
             [0,sum(Dsect['icsize'])],'w', linewidth = 2)
    plt.plot([0,sum(Dsect['icsize'])],[sum(Dsect['icsize'])-line_index,\
                          sum(Dsect['icsize'])-line_index],'w', linewidth = 2)
    line_index += Dsect['icsize'][i]

#define the new sector groupings - 2 total
sec_groups = ([1],[0,2,4,5,3])
sectors = list()
for n,k in enumerate(sec_groups):
    s = sca.Unit()
    all_items = list()
    for i in k: all_items = all_items+Dsect['ics'][i].items
    s.items = all_items
    s.col = (1/len(sec_groups))*n
    sectors.append(s)

#plot the re-ordered matrix
plt.subplot(122)
line_index=0
sortpos = list()
for s in sectors:
    sortpos.extend(s.items)
plt.imshow(Dsca['Csca'][np.ix_(sortpos, sortpos)], vmin=0, vmax=2,\
           interpolation='none',aspect='equal',\
           extent=[0,len(sortpos),0,len(sortpos)])
for s in sectors:
    plt.plot([line_index+len(s.items),line_index+len(s.items)],\
             [0,len(sortpos)],'w', linewidth = 2)
    plt.plot([0,sum(Dsect['icsize'])],[len(sortpos)-line_index, \
                              len(sortpos)-line_index],'w', linewidth = 2)
    line_index += len(s.items)
plt.tight_layout()
```

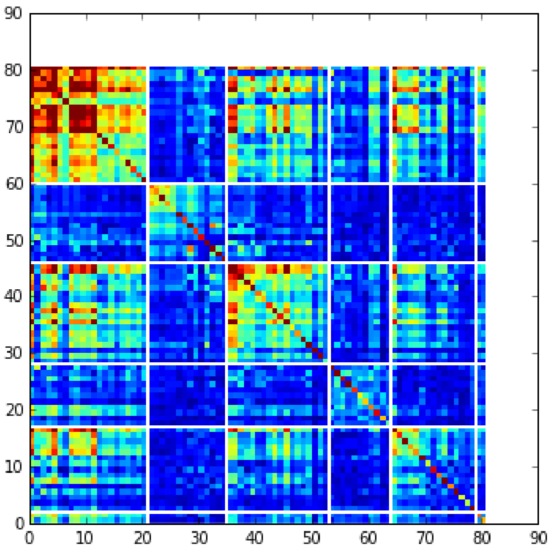

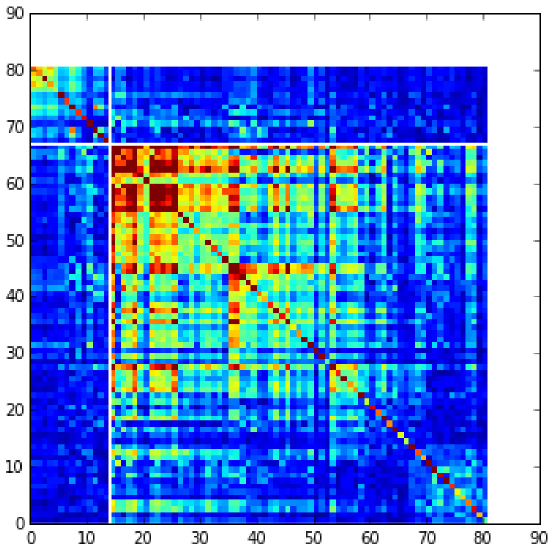

Print the sector positions, in a format suitable for pyMol, and create a pyMol session with the sectors (and decomposition into independent components) as seperate objects. Structurally, sectors 1+3 form physically contiguous units, and 2 is less so... this is consistent with the idea that sector 2/IC4 might be associated with sector 1/ICs1+3+5+6

```
In [11]: for i,k in enumerate(sectors):
             sort_ipos = sorted(k.items)
             ats_ipos = ([Dseq['ats'][s] for s in sort_ipos])
             ic_pymol = ('+'.join(ats_ipos))
             print('Sector %i is composed of %i positions:' % (i+1,len(ats_ipos)))
             print(ic_pymol + "\n")
         sca.writePymol('1FQG', sectors, Dsect['ics'], Dseq['ats'], \
                        'Outputs/PF13354.pml','A', '../Inputs/', 0)
```

```
Sector 1 is composed of 14 positions:
70+73+91+130+131+132+134+143+156+182+234+235+236+245

Sector 2 is composed of 67 positions:
65+66+68+69+71+72+85+87+97+102+105+106+107+117+119+122+123+125+126+129+136+139+144+145+149+151+153+
157+161+162+163+164+166+169+170+178+179+180+181+183+185+186+193+199+200+203+207+210+211+215+216+
220+221+225+226+229+231+233+238+240+241+244+247+250+251+255+256
```

0.1.3 III. The phylogenetic basis of the sector hierarchy

How does the clear phylogenetic heterogeneity in the MSA influence the sector definitions? To address this, we take advantage of mathematical methods for mapping between the space of positional and sequence correlations, as described in *Rivoire et al.* Using this mapping, we plot the top k_{pos} ICs as 2-D scatter plots with the corresponding sequence space divergence. The colors for the sequence space are according to the phylogenetic classifications we chose above.

```
In [12]: plt.rcParams['figure.figsize'] = 15,8
         pairs= [[0,1],[2,3],[4,5]]
         for n,[k1,k2] in enumerate(pairs):
             plt.subplot(2,3,n+1)
             sca.figUnits(Dsect['Vpica'][:,k1], Dsect['Vpica'][:,k2], sectors, dotsize = 6)
             plt.xlabel(r'$V^p_{%i}$' % (k1+1), fontsize=16)
             plt.ylabel(r'$V^p_{%i}$' % (k2+1), fontsize=16)
             plt.subplot(2,3,n+4)
             sca.figUnits(Dsect['Upica'][:,k1], Dsect['Upica'][:,k2], phylo, dotsize = 6)
             plt.xlabel(r'$U^p_{%i}$' % (k1+1), fontsize=16)
             plt.ylabel(r'$U^p_{%i}$' % (k2+1), fontsize=16)
         plt.tight_layout()
```

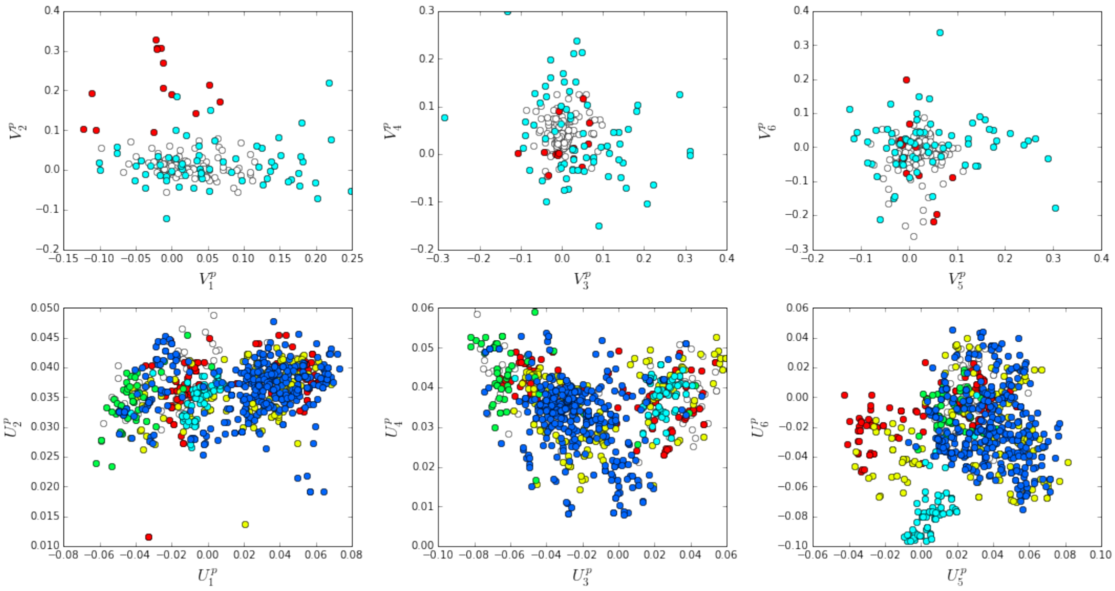

The interpretation for the two sectors:

Sector 1 is defined along (V_2^p). The sequences along the corresponding component (U_2^p) are homogeneously distributed with respect to phylogeny, consistent with the notion that this sector is a property of the entire alignment. Notably, this sector forms the catalytic core of the Beta-lactamase.

Sector 2 is composed of ICs 1,3,4 and 5 - and each of these is associated with some phylogenetic divergence. V_1^p splits the cyanobacteria *(green)* from the proteobacteria *(blue)*, V_3^p seperates the proteobacteria *(blue)* from other sequence families, V_5^p seperates out a subset of the firmicutes *(red)*, and V_6^p is associated with a divergence in the bacteriodetes *(cyan)*. Sector 2 forms a physically contiguous unit that resembles a shell around the active site. The decomposition described above suggests that some functional divergence in beta-lactamse dynamics or regulatory mechanism across phylogenetic lines may underlie the breakdown of this sector.

For clarity, we also plot the same data as a stacked bar chart below.

```
In [13]: plt.rcParams['figure.figsize'] = 20, 5

         col = list()
         for k in phylo:
             col = col + [colorsys.hsv_to_rgb(k.col,1,1)]
         for k in range(Dsect['kpos']):
             forhist = list()
             for group in phylo:
                 forhist.append([Dsect['Upica'][i,k] for i in group.items])
             plt.subplot(2,Dsect['kpos'],k+1)
             plt.hist(forhist, histtype='barstacked',color=col)
```

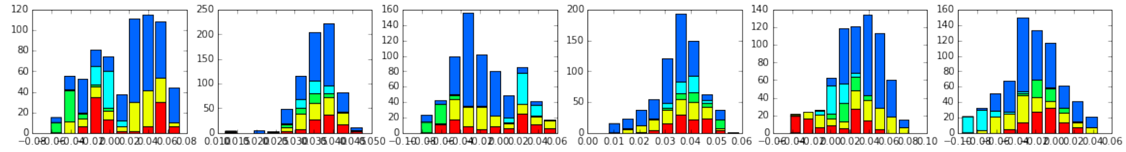

This concludes the script.
