# Supplementary figures and images for: Evolution-Based Functional Decomposition of Proteins
Source: PLoS Comput Biol. 2016 Jun 2;12(6):e1004817. doi: 10.1371/journal.pcbi.1004817 (PMC4890866; doi:10.1371/journal.pcbi.1004817)

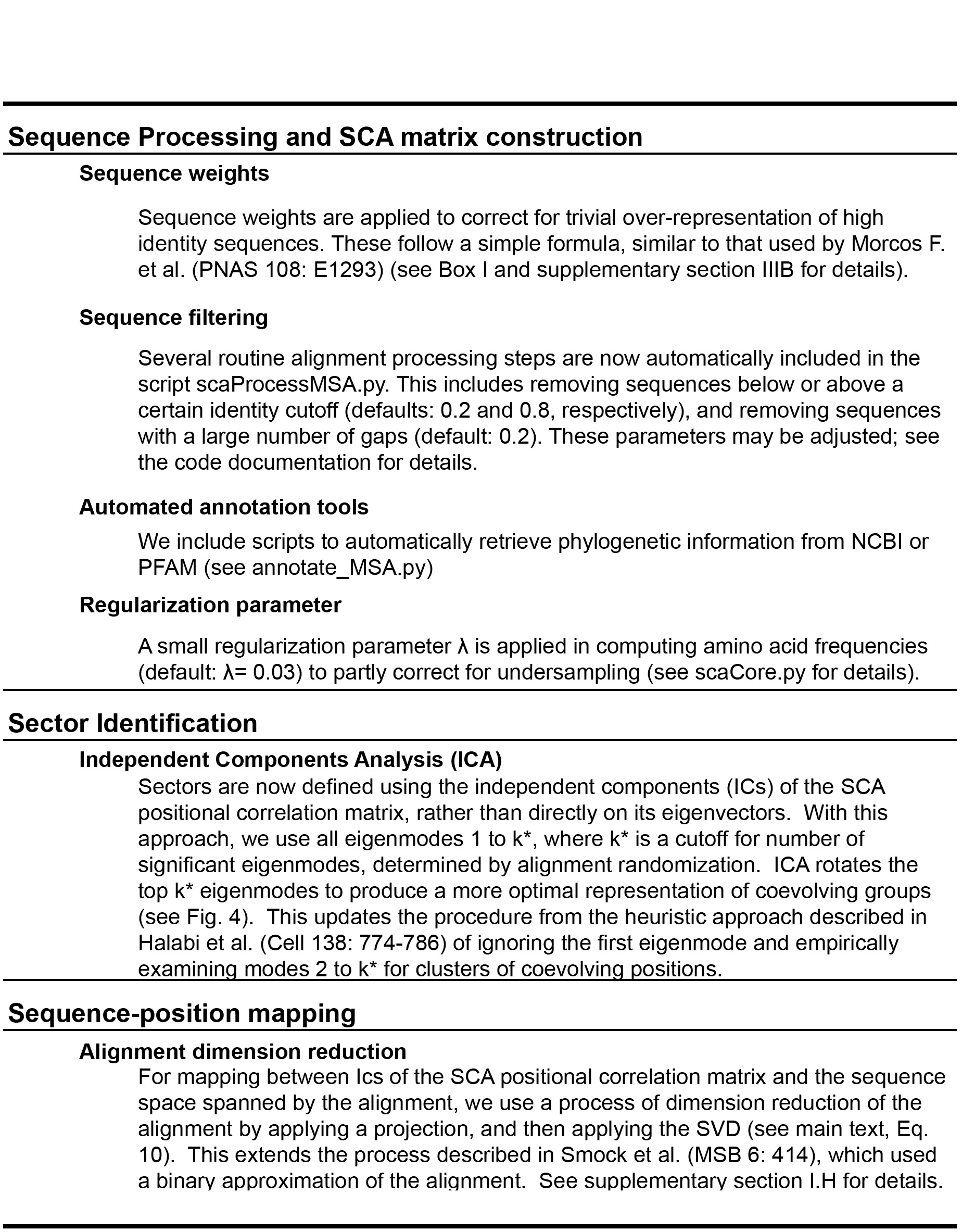

Supplement: S1 Table — (JPG) [file pcbi.1004817.s004.jpg]

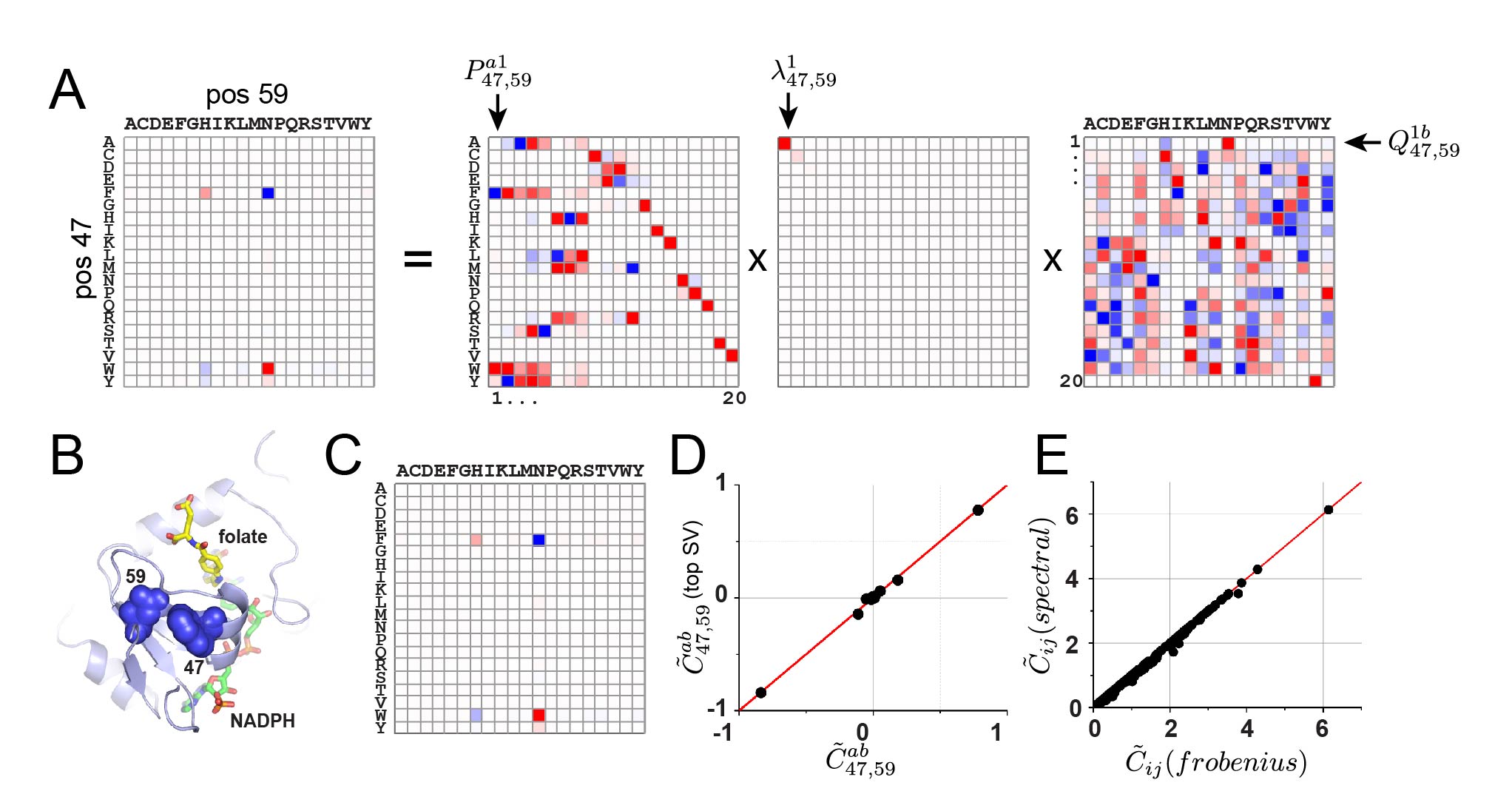

Supplement: S1 Fig — A, The amino acid correlation matrix for positions 47 and 59 (C˜47,59ab) in the dihydrofolate reductase (DHFR) alignment and the corresponding singular value decomposition. The decomposition shows the obvious dominance of the first singular value (the “spectral norm”). B, Two spatially proximal positions in DHFR chosen for illustrating properties of the SCA correlation tensor C˜ijab. C, The C˜47,59ab matrix reconstructed from just the top singular value (C˜47,59ab=P47,59a1λ47,591Q47,591b), and D, a scatterplot comparing the original and reconstructed matrices. The data demonstrate the sufficiency of the spectral norm in this case. E, the spectral norm for all pairs of positions i, j plotted against the Frobenius norm defined by (∑c(λijc)2)1/2, a measure of the magnitude of C˜ijab where all the singular values are retained. The data demonstrate the general sufficiency of the spectral norm. (JPG) [file pcbi.1004817.s005.jpg]
