# Supplementary material for: Evolution-Based Functional Decomposition of Proteins
Source: PLoS Comput Biol. 2016 Jun 2;12(6):e1004817. doi: 10.1371/journal.pcbi.1004817 (PMC4890866; doi:10.1371/journal.pcbi.1004817)
Supplement: S2 Fig — A, The histogram of eigenvalues (the “eigenspectrum”) of C˜ij for the G protein family (black bars) and for the average of N = 10 trials of random shuffling of amino acids at each position in the alignment, independently (red line) (reproduced from Fig 4A, main text). The randomization process exactly preserves the frequencies of amino acids at each position (the “first-order” statistics), but eliminates all correlations except those due to finite sampling. Since the first eigenvalue is strongly dependent on the first-order statistics, it is ignored in determining k*. The cutoff for significant eigenvalues is λ2rand+2σ, the second random eigenvalue plus two standard deviations computed over N randomization trials. Panels B-C show the robustness of the cutoff for different values of N, and E-F shows robustness over different independent trials of sub-sampling the alignment sequences to preserve the same number of effective sequences (here, Meff = 3366 out of a total of 16294 after alignment pre-processing steps). See main text and Box 1 for alignment pre-processing and calculation of number of effective sequences. The analysis shows that k* is highly robust to both number of randomization trials and to the sampling of sequences in the MSA. (PDF) [file pcbi.1004817.s006.pdf]

S2 Figure. Robustness of k^* - the number of significant eigenmodes of $\tilde{C}_{ij}$

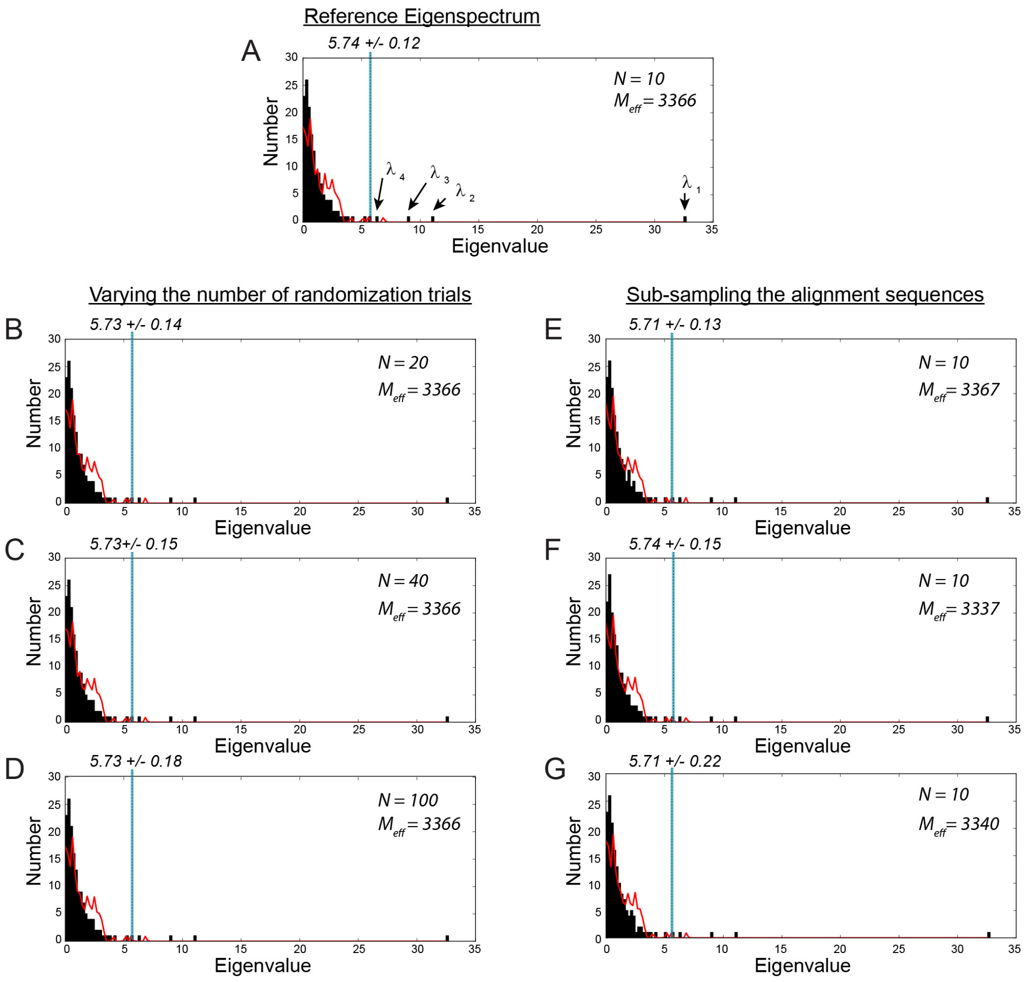

FIG. 2 **Robustness of k^* - the number of significant eigenmodes of $\tilde{C}_{ij}$ - to randomization trials and sampling of sequences.** **A**, The histogram of eigenvalues (the "eigenspectrum") of $\tilde{C}_{ij}$ for the G protein family (black bars) and for the average of $N = 10$ trials of random shuffling of amino acids at each position in the alignment, independently (red line) (reproduced from Fig. 4A, main text). The randomization process exactly preserves the frequencies of amino acids at each position (the "first-order" statistics), but eliminates all correlations except those due to finite sampling. Since the first eigenvalue is strongly dependent on the first-order statistics, it is ignored in determining k^*. The cutoff for significant eigenvalues is $\lambda_2^{rand} + 2\sigma$, the second random eigenvalue plus two standard deviations computed over N randomization trials. Panels **B-C** show the robustness of the cutoff for different values of N, and **E-F** shows robustness over different independent trials of sub-sampling the alignment sequences to preserve the same number of effective sequences (here, $M_{eff} = 3366$ out of a total of 16294 after alignment pre-processing steps). See main text and Box 1 for alignment pre-processing and calculation of number of effective sequences. The analysis shows that k^* is highly robust to both number of randomization trials and to the sampling of sequences in the MSA.
