# Supplementary material for: Evolution-Based Functional Decomposition of Proteins
Source: PLoS Comput Biol. 2016 Jun 2;12(6):e1004817. doi: 10.1371/journal.pcbi.1004817 (PMC4890866; doi:10.1371/journal.pcbi.1004817)
Supplement: S3 Fig — A-C, As described in S1 Text, section H, S1 Fig, one property of the SCA correlation tensor C˜ijab is compressibility, such that the information in each 20 × 20 amino acid coevolution matrix for each pair of positions (i, j) can be represented by a scalar value λij1, the top singular value (B). Per the SVD (A), the top singular value is associated with the top left and right singular vectors Pija1 and Q1bij, which contain the weights for the contributions of amino acids at positions i and j, respectively. Since coevolution is a symmetric property of amino acids at two positions (C˜ijab=C˜jiba, we can further simplify the SVD further as in C. D-E, Besides compressibility, another empirical property of C˜ijab is that for any given position i, the top singular vector Pi,ja1 is essentially invariant over all j; that is the amino acids by which position i coevolves with other positions j is nearly the same. For example, for three positions within the core of the G protein (D, positions 82, 125, and 130), the amino acids by which other positions i coevolve with these positions varies, but the amino acids by which these positions coevolve with other positions j is nearly the same (E. Thus, it is possible to define a projection for each position (S1 Text. Eq 19) by which the alignment tensor xsia can be reduced to an alignment matrix xsi (S1 Text, Eq 20). (PDF) [file pcbi.1004817.s007.pdf]

S3 Figure. The pattern of amino acid contributions to positional coevolution

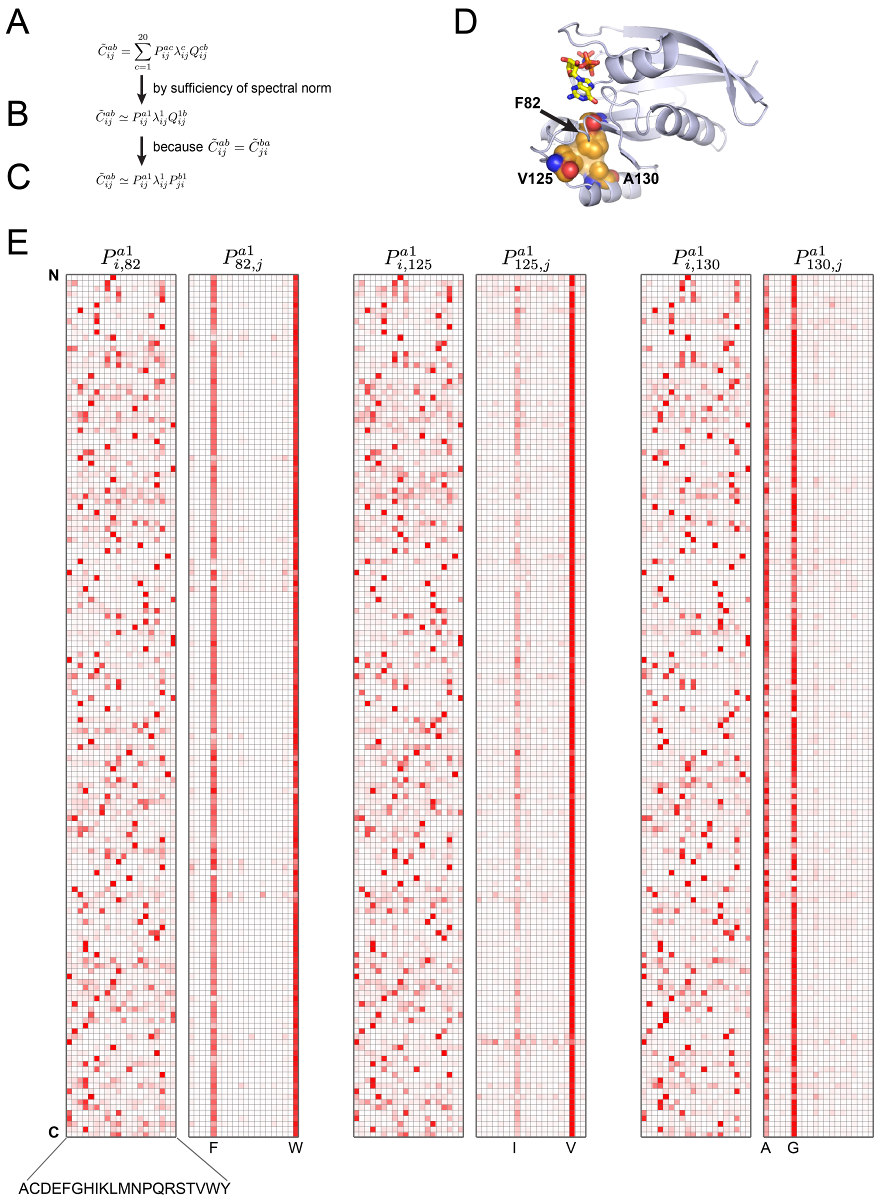

FIG. 3 **The pattern of amino acid contributions to positional coevolution A-C**, As described in S1 Text, section H, S1 Figure, one property of the SCA correlation tensor $\tilde{C}_{ij}^{ab}$ is compressibility, such that the information in each 20×20 amino acid coevolution matrix for each pair of positions (i, j) can be represented by a scalar value λ_{ij}^{1}, the top singular value (**B**). Per the SVD (**A**), the top singular value is associated with the top left and right singular vectors P_{ij}^{a1} and $Q1b_{ij}$, which contain the weights for the contributions of amino acids at positions i and j, respectively. Since coevolution is a symmetric property of amino acids at two positions ($\tilde{C}_{ij}^{ab} = \tilde{C}_{ji}^{ba}$, we can further simplify the SVD further as in **C**. **D-E**, Besides compressibility, another empirical property of $\tilde{C}_{ij}^{ab}$ is that for any given position i, the top singular vector $P_{i,j}^{a1}$ is essentially invariant over all j; that is the amino acids by which position i coevolves with other positions j is nearly the same. For example, for three positions within the core of the G protein (**D**, positions 82, 125, and 130), the amino acids by which other positions i coevolve with these positions varies, but the amino acids by which these positions coevolve with other positions j is nearly the same (**E**. Thus, it is possible to define a projection for each position (S1 Text. Eq. 19) by which the alignment tensor x_{si}^{a} can be reduced to an alignment matrix x_{si} (S1 Text, Eq. 20).
