# Supplementary material for: Evolution-Based Functional Decomposition of Proteins
Source: PLoS Comput Biol. 2016 Jun 2;12(6):e1004817. doi: 10.1371/journal.pcbi.1004817 (PMC4890866; doi:10.1371/journal.pcbi.1004817)
Supplement: S4 Fig — A-F show the positions comprising the six ICs of the SCA coevolution matrix, respectively, as colored spheres on the structure of E. coli DHFR (PDB 1RX2), and G-L show stacked histograms of the corresponding sequence space colored colored by phylogenetic annotation. The data show that ICs 1, 5, and 6 roughly separate eukaryotic and prokaryotic sequences and that the remainder are more homogeneous with regard to phylogenetic divergence. The significance of these apparent heterogeneities will require further investigation, but prior work demonstrates functional and mechanistic differences between the eukaryotic and prokaryotic members of this protein family [65]. (PDF) [file pcbi.1004817.s008.pdf]

S4 Figure. Sequence-position mapping for the DHFR protein family

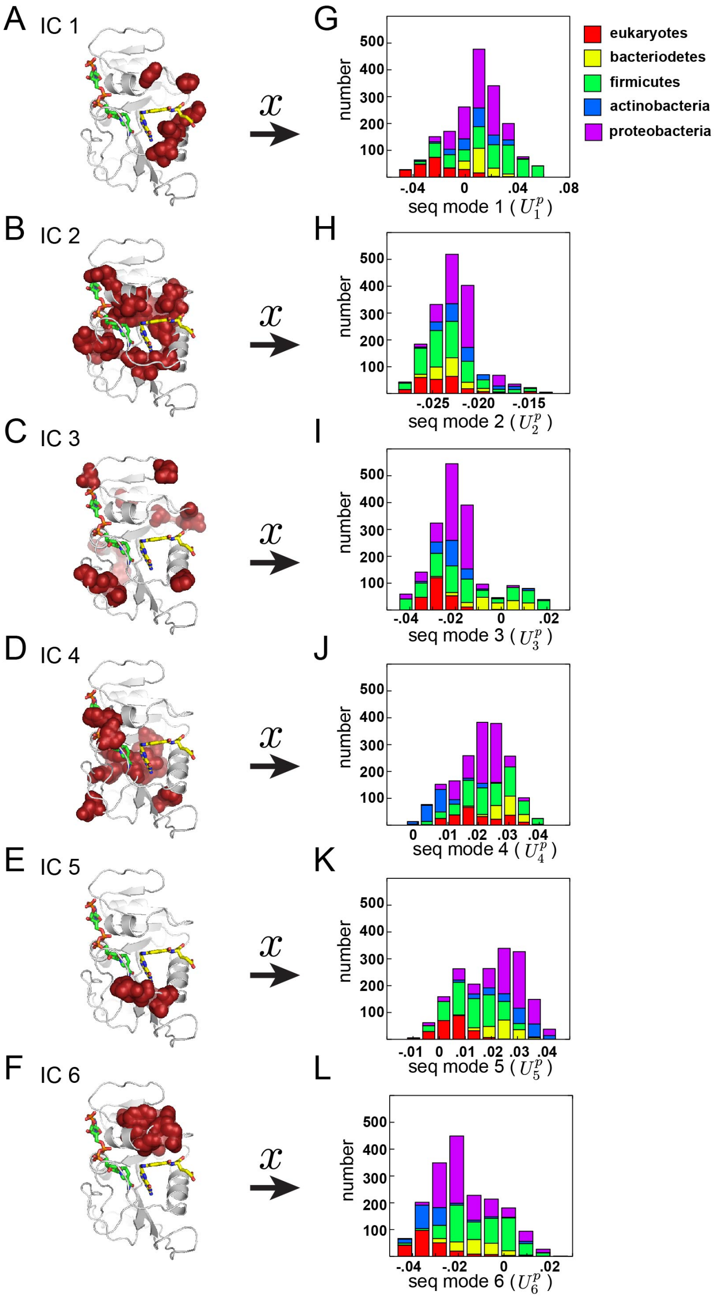

FIG. 4 **Sequence-position mapping for the DHFR protein family. A-F** show the positions comprising the six ICs of the SCA coevolution matrix (Fig. S3A), respectively, as colored spheres on the structure of E. coli DHFR (PDB 1RX2), and **G-L** show stacked histograms of the corresponding sequence space colored colored by phylogenetic annotation. The data show that ICs 1, 5, and 6 roughly separate eukaryotic and prokaryotic sequences and that the remainder are more homogeneous with regard to phylogenetic divergence. The significance of these apparent heterogeneities will require further investigation, but prior work demonstrates functional and mechanistic differences between the eukaryotic and prokaryotic members of this protein family (**?**).
