# Supplementary material for: Evolution-Based Functional Decomposition of Proteins
Source: PLoS Comput Biol. 2016 Jun 2;12(6):e1004817. doi: 10.1371/journal.pcbi.1004817 (PMC4890866; doi:10.1371/journal.pcbi.1004817)
Supplement: S5 Fig — The independent components (ICs) corresponding to the four top eigenmodes of the C˜ij matrix. The solid red line is a fit to the t-distribution, with the cutoff indicated representing the top 95% of the cumulative density function. The ICs are generally well-fit by this empirical distribution, and serves as a basis for systematic definition of coevolving positions. (PDF) [file pcbi.1004817.s009.pdf]

S5 Figure. Independent components of the G protein family

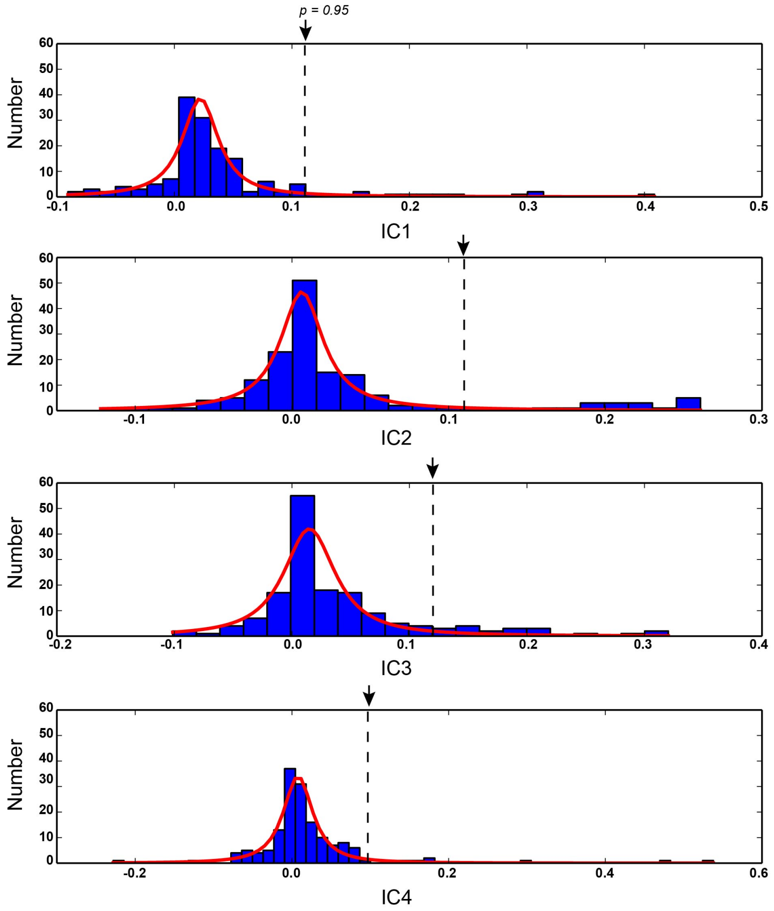

FIG. 5 **The independent components (ICs) corresponding to the four top eigenmodes of the $\tilde{C}_{ij}$ matrix for the G protein family**. The solid red line is a fit to the t-distribution, with the cutoff indicated representing the top 95% of the cumulative density function. The ICs are generally well-fit by this empirical distribution, and serves as a basis for systematic definition of coevolving positions.
