# Supplementary material for: Evolution-Based Functional Decomposition of Proteins
Source: PLoS Comput Biol. 2016 Jun 2;12(6):e1004817. doi: 10.1371/journal.pcbi.1004817 (PMC4890866; doi:10.1371/journal.pcbi.1004817)
Supplement: S6 Fig — Scatterplots of the top four independent components (ICs) for the full alignment of G proteins (Meff = 3366) against those for four trials of sub-sampling the alignment to ≈15% of sequences. The analysis shows that the composition of ICs (and therefore sector definitions and sequence projections) are highly robust to the number of effective sequences in the alignment. (PDF) [file pcbi.1004817.s010.pdf]

S6 Figure. Robustness of the independent components

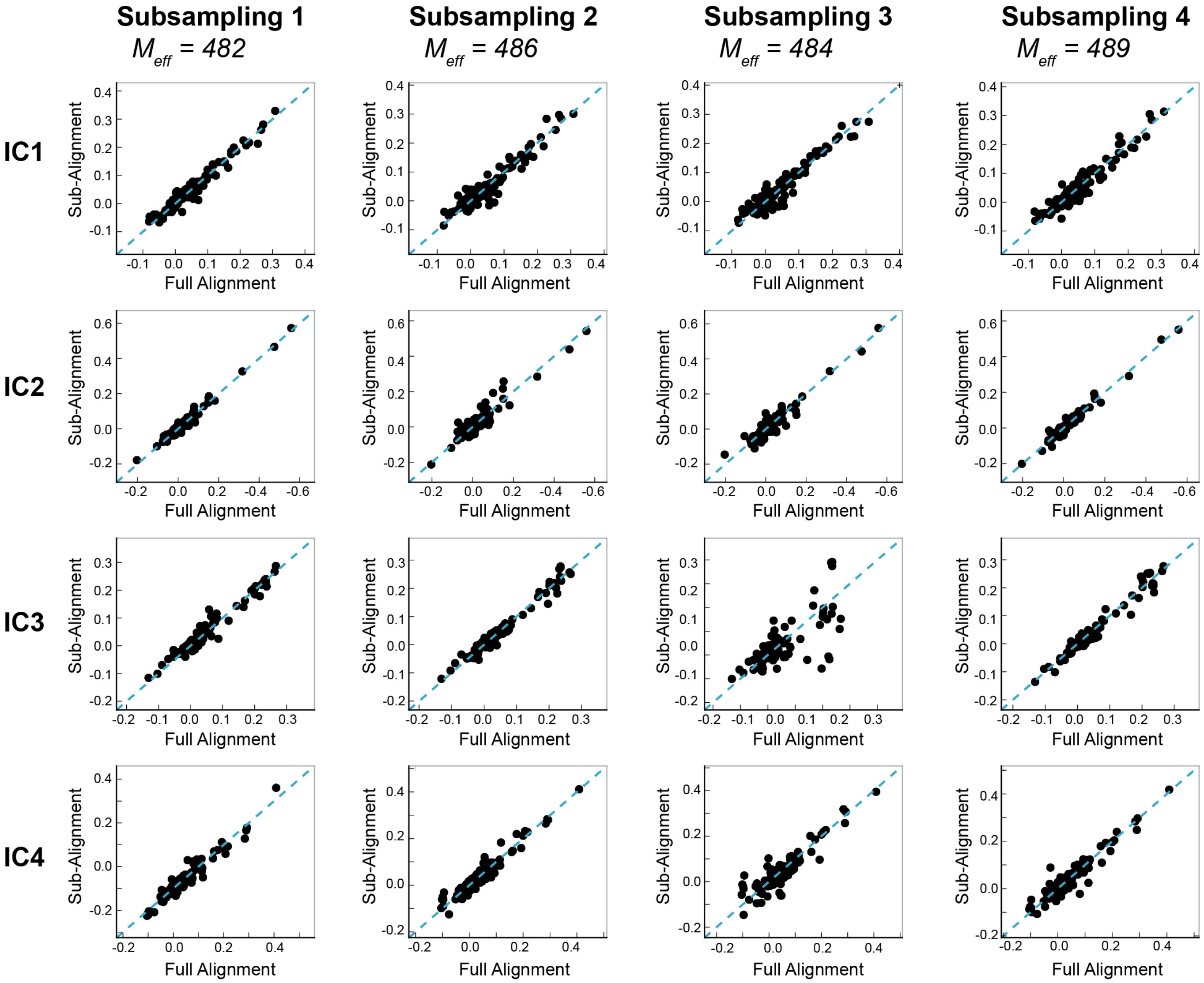

FIG. 6 **The robustness of the independent components (ICs) of the $\tilde{C}_{ij}$ matrix to alignment sub-sampling.** Scatterplots of the top four independent components (ICs) for the full alignment of G proteins ($M_{eff} = 3366$) against those for four trials of sub-sampling the alignment to $\approx 15\%$ of sequences. The analysis shows that the composition of ICs (and therefore sector definitions and sequence projections) are highly robust to the number of effective sequences in the alignment.
