# Supplementary material for: Evolution-Based Functional Decomposition of Proteins
Source: PLoS Comput Biol. 2016 Jun 2;12(6):e1004817. doi: 10.1371/journal.pcbi.1004817 (PMC4890866; doi:10.1371/journal.pcbi.1004817)
Supplement: S7 Fig — A shows the IC-based sub-matrix of the C˜ij matrix for the S1A family and B-G shows the positions corresponding to each IC on a representative structure of a member of the protein family (rat trypsin, PDB 3TGI). Each IC shows a hierarchical pattern of correlation between constituent positions, with little compelling evidence for strong inter-IC correlations. Consistent with this, each IC corresponds to a distinct and largely contiguous network of amino acid contacts in the protein structure (B). ICs 1–3 correspond to sectors defined in Halabi et al. [10]. (PDF) [file pcbi.1004817.s011.pdf]

S7 Figure. Sectors in the S1A protein family

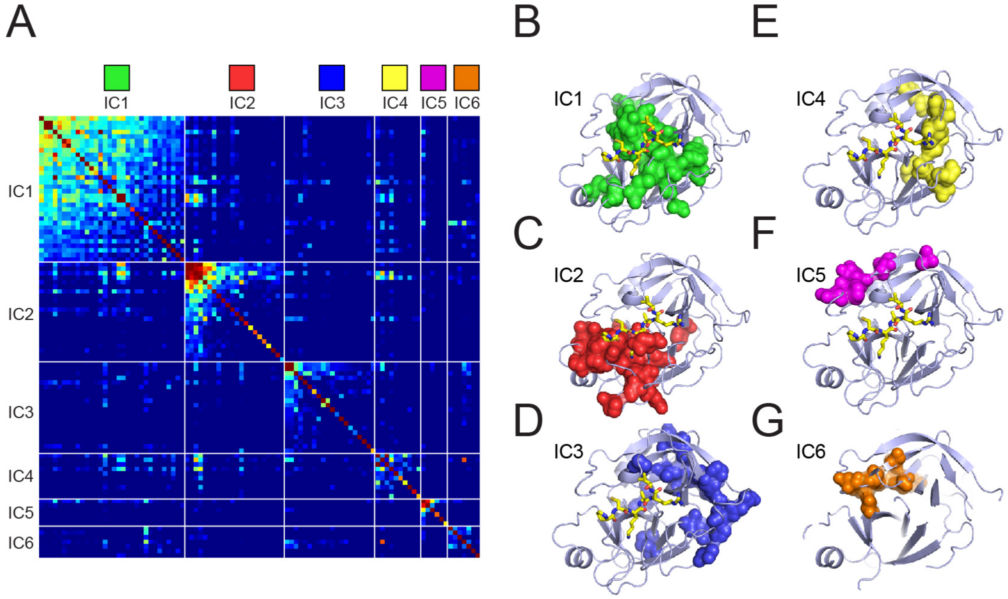

FIG. 7 **Sectors in the S1A protein family. A** shows the IC-based sub-matrix of the $\tilde{C}_{ij}$ matrix for the S1A family and **B-G** shows the positions corresponding to each IC on a representative structure of a member of the protein family (rat trypsin, PDB 3TGI). Each IC shows a hierarchical pattern of correlation between constituent positions, with little compelling evidence for strong inter-IC correlations. Consistent with this, each IC corresponds to a distinct and largely contiguous network of amino acid contacts in the protein structure (**B**). ICs 1-3 correspond to sectors defined in Halabi et al. Cell (2009) 138: 774-86.
