# Supplementary material for: Evolution-Based Functional Decomposition of Proteins
Source: PLoS Comput Biol. 2016 Jun 2;12(6):e1004817. doi: 10.1371/journal.pcbi.1004817 (PMC4890866; doi:10.1371/journal.pcbi.1004817)
Supplement: S8 Fig — Panels A and C show the IC-based sub-matrix of the Cij˜ matrix for the DHFR and the β-lactamase protein families. The cartoons at right indicate the sector analyses. DHFR displays considerable transitive external correlations between ICs, suggesting a single sector. The β-lactamase family displays two sectors, one comprising IC2 and the other comprising ICs1, and 3–6. Panels B and D show the positions contributing to each IC mapped to the protein structure; in each case the sectors form physically contiguous structural units. (PDF) [file pcbi.1004817.s012.pdf]

S8 Figure. Sectors in the DHFR and β-lactamase protein families

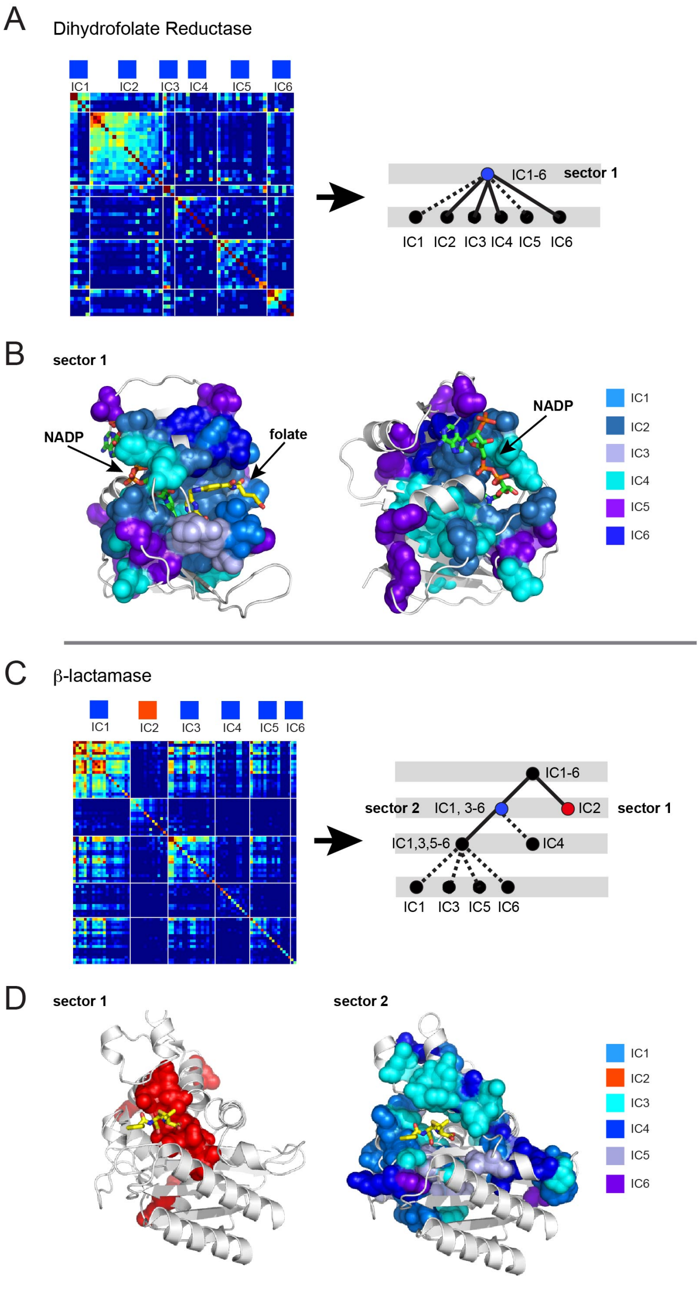

FIG. 8 **Sector identification for the DHFR and β-lactamase protein families.** Panels **A** and **C** show the IC-based sub-matrix of the $\tilde{C}_{ij}$ matrix for the DHFR and the β-lactamase protein families. The cartoons at right indicate the sector analyses. DHFR displays considerable transitive external correlations between ICs, suggesting a single sector. The β-lactamase family displays two sectors, one comprising IC2 and the other comprising ICs1, and 3-6. Panels B and D show the positions contributing to each IC mapped to the protein structure; in each case the sectors form physically contiguous structural units.
